# Plasma Lipid Profiling Shows Similar Associations with Prediabetes and Type 2 Diabetes

**DOI:** 10.1371/journal.pone.0074341

**Published:** 2013-09-27

**Authors:** Peter J. Meikle, Gerard Wong, Christopher K. Barlow, Jacquelyn M. Weir, Melissa A. Greeve, Gemma L. MacIntosh, Laura Almasy, Anthony G. Comuzzie, Michael C. Mahaney, Adam Kowalczyk, Izhac Haviv, Narelle Grantham, Dianna J. Magliano, Jeremy B. M. Jowett, Paul Zimmet, Joanne E. Curran, John Blangero, Jonathan Shaw

**Affiliations:** 1 Baker IDI Heart and Diabetes Institute, Melbourne, Victoria, Australia; 2 Department of Genetics, Texas Biomedical Research Institute, San Antonio, Texas, United States of America; 3 National ICT Australia (NICTA), University of Melbourne, Melbourne, Victoria, Australia; Fundação Oswaldo Cruz, Brazil

## Abstract

The relationship between lipid metabolism with prediabetes (impaired fasting glucose and impaired glucose tolerance) and type 2 diabetes mellitus is poorly defined. We hypothesized that a lipidomic analysis of plasma lipids might improve the understanding of this relationship. We performed lipidomic analysis measuring 259 individual lipid species, including sphingolipids, phospholipids, glycerolipids and cholesterol esters, on fasting plasma from 117 type 2 diabetes, 64 prediabetes and 170 normal glucose tolerant participants in the Australian Diabetes, Obesity and Lifestyle Study (AusDiab) then validated our findings on 1076 individuals from the San Antonio Family Heart Study (SAFHS). Logistic regression analysis of identified associations with type 2 diabetes (135 lipids) and prediabetes (134 lipids), after adjusting for multiple covariates. In addition to the expected associations with diacylglycerol, triacylglycerol and cholesterol esters, type 2 diabetes and prediabetes were positively associated with ceramide, and its precursor dihydroceramide, along with phosphatidylethanolamine, phosphatidylglycerol and phosphatidylinositol. Significant negative associations were observed with the ether-linked phospholipids alkylphosphatidylcholine and alkenylphosphatidylcholine. Most of the significant associations in the AusDiab cohort (90%) were subsequently validated in the SAFHS cohort. The aberration of the plasma lipidome associated with type 2 diabetes is clearly present in prediabetes, prior to the onset of type 2 diabetes. Lipid classes and species associated with type 2 diabetes provide support for a number of existing paradigms of dyslipidemia and suggest new avenues of investigation.

## Introduction

In Australia and globally the obesity epidemic is associated with an increase in the prevalence of type 2 diabetes mellitus (T2D). If the current trend continues in Australia, the prevalence of T2D is projected to rise from 7.6% in 2000 to 11.4% by 2025 [1]. More than a third of individuals will develop T2D within their lifetime and there will be an additional 1 million Australians with T2D by the year 2025 [1]. Dyslipidemia (raised plasma triglycerides and decreased HDL-cholesterol) is independently associated with T2D. However, triglycerides represent a large number of individual molecular species while HDL and other lipoproteins consist of many different lipid classes containing multiple molecular species within each class. The relationships between the individual molecular species of lipid and T2D have not been fully investigated.

Over the past decade, the “omics” revolution has expanded to embrace “lipidomics” as a major contributor to our understanding of biological processes in health and disease. Recent studies have identified lipids associated with T2D [2] and coronary artery disease [3] as well as changes in lipid metabolism in response to therapeutics, including statins [4] and metformin [5]. Other studies have demonstrated a link between increased lipotoxicity, including increased synthesis of fatty acids, sphingolipids and phospholipids, and the development of diabetic nephropathy [6], [7], [8] and that progression to different stages of diabetic nephropathy in T2D patients is associated with differential alterations in non-esterified and esterified fatty acids [6], [8]. Lipidomics is also starting to identify potential biomarkers for risk assessment in T2D and cardiovascular disease [3], [8].

Current technology provides the ability to measure many hundreds of lipid species from a few µL of blood. Given the intimate links between carbohydrate and lipid metabolism, it seems likely that exploration of the associations between diabetes and a wide range of lipid species will provide insights into the pathophysiology of T2D. In order to investigate these associations we performed plasma lipid profiling on participants from the Australian Diabetes, Obesity and Lifestyle Study (AusDiab) study (normal glucose tolerance, prediabetes and newly diagnosed T2D; total n = 351), then validated these findings on an independent population cohort (n = 1076) from the San Antonio Family Heart Study (SAFHS).

## Materials and Methods

### Ethics Statement

This study was approved by the Alfred Hospital, Ethics Committee, Project No: 104/10.

### Participants

AusDiab was established to measure the prevalence of T2D and risk factors for T2D and cardiovascular disease (CVD) in a national population-based cohort. Baseline testing (in 1999–2000) involved 11,247 adults aged ≥25 years residing in 42 randomly selected areas of the six states of Australia and the Northern Territory [9]. Plasma samples were collected and stored at −75°C. Demographic information, smoking history, alcohol intake, dietary intake, history of CVD and T2D were collected by questionnaire, and blood pressure and anthropometrics were measured. A two-hour oral glucose tolerance test (OGTT), fasting plasma lipids, insulin and HbA1c were determined. Written informed consent was obtained from all participants. The study group selected from the AusDiab baseline cohort consisted of 117 participants (58 men and 59 women), non-smoking, with newly diagnosed T2D (FPG ≥7.0 mM and/or 2h post load glucose ≥11.1 mM) and BMI between 20 and 35 kg/m^2^. These were matched for age and sex with 234 non-smoking, T2D-free controls. The control group contained 64 participants with either impaired glucose tolerance (2h post-load glucose 7.8–11.0 mM, n = 50) or impaired fasting glucose (FPG 6.1–6.9 mM, n = 19), collectively referred to as prediabetes. Five participants with IGT also had IFG. The participant characteristics are shown in Table 1.

**Table 1:** Baseline characteristics of the AusDiab study cohort.

Characteristic^a^	NGT	Prediabetes^b^	T2D	NGT vs	NGT vs	Prediabetes
	(n = 170)	(n = 64)	(n = 117)	Prediabetes	T2D	vs T2D
Sex (% male)^c^	47.7 (81/170)	53.1 (34/64)	51.3 (60/117)	0.455	0.545	0.812
Age (years)^d^	60 (49–72)	69 (58–74)	62 (52–73)	**0.004**	0.603	0.083
Waist^d^	90.3 (83.3–98.2)	93.6 (88.1–100.3)	96.5 (89.0–103.9)	0.094	<**0.001**	0.183
BMI^d^	26.0 (23.6–27.9)	26.4 (24.5–29.0)	27.9 (25.5–30.7)	0.637	<**0.001**	**0.018**
Central obesity (%)^c^	29.4 (50/170)	42.2 (27/64)	57.3 (67/117)	0.067	<**0.001**	0.052
SBP (mm Hg)^d^	133 (121–146)	138 (127–155)	143 (131–154)	**0.031**	<**0.001**	0.969
FPG (mM)^d^	5.3 (5.1–5.6)	5.7 (5.4–6.2)	6.9 (5.7–7.4)	<**0.001**	<**0.001**	<**0.001**
2h-PLG (mM)^d^	5.8 (4.8–6.6)	8.6 (7.0–9.6)	11.8 (10.7–13.3)	<**0.001**	<**0.001**	<**0.001**
HbA1c (mM)^d^	5.11 (4.97–5.26)	5.33 (5.13–5.47)	5.55 (5.31–6.07)	<**0.001**	<**0.001**	<**0.001**
Insulin (pmol/L)^d^	83.7 (68.8–101.4)	84.4 (68.4–118.5)	125.0 (79.5–164.9)	0.674	<**0.001**	<**0.001**
Chol (mM)^d^	5.7 (5.1–6.4)	6.0 (5.3–6.8)	5.8 (5.3–6.6)	0.059	0.127	0.880
HDL (mM)^d^	1.42 (1.20–1.67)	1.33 (1.09–1.59)	1.22 (1.04–1.53)	0.139	**0.001**	0.678
LDL (mM)^d^	3.67 (3.00–4.23)	3.72 (3.02–4.62)	3.70 (3.15–4.25)	0.687	0.995	0.869
Triglyceride (mM)^d^	1.20 (0.90–1.55)	1.84 (1.25–2.40)	1.94 (1.29–2.89)	<**0.001**	<**0.001**	0.645

a BMI, body mass index; SBP, systolic blood pressure; FPG, fasting plasma glucose; 2h-PLG, 2 hour-post load glucose; HbA1c, glycated haemoglobin; Chol, total cholesterol; HDL, high density cholesterol; LDL, low density cholesterol.

b Prediabetes group consists of 24 IFG and 45 IGT.

c Values expressed as % (number/total), p-values (Chi-squared test).

d Data are expressed as median (interquartile range), p-values (Mann-Whitney U test with Dunn-Sidak correction).

The SAFHS commenced in 1991 and was designed to investigate the genetics of cardiovascular disease and its risk factors in Mexican Americans. The SAFHS included 1,431 individuals in 42 extended families [10]. Questionnaires and anthropometric measurements were performed. Plasma cholesterol, HDL-C, triglycerides, glucose and insulin were also measured. Plasma samples were collected and stored at −75°C. In this population, we were able to access samples from the whole study, and analysed samples from 1,076 participants for which plasma and complete data were available (126 prediabetes, 142 T2D of whom 69 were not on any medication). The participant characteristics are shown in Table 2.

**Table 2:** Baseline characteristics of the SAHFS study cohort.

Characteristic^a^	NGT^b^	Prediabetes^c^	T2D^d^	NGT vs	NGT vs	Prediabetes
	(n = 808)	(n = 126)	(n = 142)	Prediabetes	T2D	vs T2D
Sex (% male)^e^	40.6 (328/808)	32.5% (41/126)	36.6 (52/142)	0.085	0.372	0.484
Age (years)^f^	31 (23–44)	46 (36–54)	51 (44–63)	<**0.001**	<**0.001**	**0.001**
Waist^f^	90.0 (79.7–100.0)	103.0 (92.4–18.5)	102.8 (96.2–116.5)	<**0.001**	<**0.001**	0.705
BMI^f^	27.5 (24.0–31.5)	31.5 (27.8–36.6)	30.9 (28.5–36.0)	<**0.001**	<**0.001**	1.000
Central obesity (%)^e^	37.9 (306/808)	77.8 (98/126)	73.9 (105/142)	<**0.001**	<**0.001**	0.465
SBP (mm Hg)^f^	114 (106–123)	121 (112–138)	131 (118–147)	<**0.001**	<**0.001**	**0.001**
FPG (mM)^f^	4.7 (4.4–5.0)	5.30 (4.9–5.7)	8.8 (7.1–12.5)	<**0.001**	<**0.001**	<**0.001**
2h-PLG (mM)^f^	5.1 (4.3–6.0)	8.5 (7.9–9.3)	17.8 (14.0–20.7)	<**0.001**	<**0.001**	<**0.001**
Insulin (pmol/L)^f^	66.9 (43.6–103.3)	107.5 (70.8–164.9)	122.2 (77.8–221.2)	<**0.001**	<**0.001**	0.530
Chol (mM)^f^	4.7 (4.1–5.4)	5.1 (4.4–5.7)	5.2 (4.6–6.1)	**0.001**	<**0.001**	0.503
HDL (mM)^f^	1.24 (1.09–1.50)	1.24 (1.03–1.42)	1.20 (1.03–1.45)	0.493	0.067	0.876
LDL (mM)^f^	2.83 (2.29–3.37)	2.99 (2.46–3.50)	3.06 (2.53–3.71)	0.191	**0.002**	0.606
Triglyceride (mM)^f^	1.22 (0.87–1.66)	1.84 (1.26–2.42)	1.94 (1.46–2.55)	<**0.001**	<**0.001**	0.213

a BMI, body mass index; SBP, systolic blood pressure; FPG, fasting plasma glucose; 2h-PLG, 2 hour-post load glucose; HbA1c, glycated haemoglobin; Chol, total cholesterol; HDL, high density lipoprotein cholesterol; LDL, low density lipoprotein cholesterol.

b NGT group contains 12 individuals on lipid lowering medication.

c Prediabetes group consists of 14 IFG and 112 IGT, 4 individuals were on diabetes or lipid lowering medication.

d Type 2 diabetes group consists of 69 untreated and 73 treated (diabetes or lipid lowering medication).

e Values expressed as % (number/total), p-values (Chi-squared test).

f Expressed as median (interquartile range), p-values (Mann-Whitney U test with Dunn-Sidak correction).

### Sample preparation and lipid extraction

The order of the plasma samples was randomised prior to lipid extraction and analysis for each cohort. Quality control plasma samples were included at a ratio of 1∶11 (AusDiab) and 1∶18 (SAFHS). Total lipid extraction from a 10 µL aliquot of plasma was performed by a single phase chloroform:methanol (2∶1) extraction [3].

### High performance liquid chromatography-mass spectrometry analysis

Lipid analysis was performed by liquid chromatography, electrospray ionisation-tandem mass spectrometry using a Agilent 1200 liquid chromatography system combined with an Applied Biosystems API 4000 Q/TRAP mass spectrometer with a turbo-ionspray source (350°C) and Analyst 1.5 data system [3]. We have previously reported the use of precursor ion and neutral loss scans on control plasma extracts to identify the predominant lipid species of the following lipid classes: dihydroceramide (dhCer), ceramide (Cer), monohexosylceramide (MHC), dihexosylceramide (DHC), trihexosylcermide (THC), G_M3_ ganglioside (GM3), sphingomyelin (SM), phosphatidylcholine (PC), alkylphosphatidylcholine (PC(O)), alkenylphosphatidylcholine (plasmalogen, PC(P)), lysophosphatidylcholine (LPC), lysoalkylphosphatidylcholine (lysoplatelet activating factor, LPC(O)), phosphatidylethanolamine (PE), phosphatidylinositol (PI), phosphatidylserine (PS), phosphatidylglycerol (PG), cholesterol ester (CE), free cholesterol (COH), diacylglycerol (DG) and triaclyglycerol (TG) [3], [11], [12]. The abbreviations shown above are only used when referring to individual lipid species as in LPC 22∶6 which defines a lysophosphatidylcholine with a fatty acid containing 22 carbons and six double bonds. For a number of the lipids which contain two fatty acid chains the mass spectrometry based measurements here do not directly determine the constituent fatty acids but rather the sum of the number of carbons and the sum of the number of double bonds across both fatty acids. Accordingly, we denote these species as the combined length and number of double bonds, e.g. PC 36∶4. It is worth noting however that based on previous work by us and others the identity of at least the major fatty acids making up such a species in plasma may be reasonably inferred.

Multiple Reaction Monitoring (MRM) experiments were established for the major species of each lipid class identified in plasma. A total of 65 diacylglycerol and triacylglycerol species and 194 other lipid species were analysed in two separate experiments. Relative lipid amounts were calculated by relating the peak area of each species to the peak area of the corresponding stable isotope or non-physiological internal standard. Total lipid classes were calculated from the sum of the individual lipid species within each class [3].

### Statistical analysis

Percentage coefficient of variation for each lipid species across each analytical run (AusDiab or SAFHS) was calculated as the standard deviation divided by the mean of the quality control samples. Binary logistic regression adjusted for age, sex, waist circumference and systolic blood pressure (SBP) was used to determine the strength of association of each lipid species and each lipid class with T2D and prediabetes. Linear regression adjusted for age, sex, waist circumference and SBP was used to describe the linear relationship between lipids and FPG or 2h-PLG. Samples with missing covariate values were excluded from the analysis. The odds ratio obtained for each lipid or lipid class represents the number of times an individual with a lipid measurement in the 75^th^ percentile is more likely to have T2D or prediabetes than an individual with a lipid measurement in the 25^th^ percentile. The beta-coefficients obtained represent the change in outcome measure (FPG or 2h-PLG) associated with an interquartile range increase in the lipid measurement. All p-values obtained were corrected for multiple comparisons using the Benjamini-Hochberg approach [13]. A corrected p-value of <0.05 was considered to be statistically significant. Linear and logistic regression were initially performed on subjects from the AusDiab cohort and subsequently repeated on the SAFHS cohort. Pearson's linear correlation was used to determine the agreement of odds ratios and beta-coefficients between the two cohorts.

## Results

Preliminary analyses showed that the lipid profiles characterising T2D and prediabetes were similar to each other, and that restricting analyses to comparisons of those with and without diabetes might mask important findings. Results are therefore presented separately for the three groups (NGT, prediabetes and T2D).

### Study cohort

In the AusDiab cohort the median age of the prediabetes group was greater than the NGT group and the T2D group (Table 1). There was no difference in waist circumference between the T2D and prediabetes groups but mean waist circumference was significantly higher in the T2D group compared to the NGT group. Both T2D and prediabetes showed a higher SBP, total cholesterol and triglyceride concentrations relative to the NGT group. FPG, 2h-PLG, HbA1c and fasting insulin levels were also significantly different between groups (Table 1).

The SAFHS cohort had a prevalence of T2D of 13.2% (6.4% not on any medication) and a prevalence of prediabetes (IFG or IGT) of 11.7%. Comparing SAFHS to AusDiab, SAFHS had fewer men (39.1% vs 49.9%), and were significantly younger (median age of 35.7 vs 61.7 years), and SBP was correspondingly lower (median of 117 vs 140 mmHg). Measures of plasma lipids were also substantially lower in the SAFHS cohort (Table 2).

### Lipid measurements

Determination of relative lipid levels was performed using scheduled multiple-reaction monitoring (MRM) in positive ion mode [3], [11], [12], [14], [15], [16] (Table S1 in File S1). The median percentage coefficient of variation (% CV) for the individual lipid species within the quality control samples of the AusDiab analysis was 10.3% with 90% of lipids below 17.0%. In the SAFHS analysis the median % CV was 14.3% with 90% of lipids below 24.7%.

### Association of lipids with type 2 diabetes and prediabetes

When the relative amounts of the lipid species within each lipid class were summed (total lipid classes), we found that, compared to the NGT group, and after adjusting for age, sex, waist circumference and SBP, each of the following lipid classes were significantly associated with the presence of T2D and with prediabetes: dihydroceramide, ceramide, alkylphosphatidylcholine, phosphatidylethanolamine, phosphatidylinositol, phosphatidylglycerol, cholesterol ester, diacylglycerol and triacylglycerol, (all p<0.05, Table 3). Alkenylphosphatidylcholine showed a similar effect size to alkylphosphatidylcholine (OR = 0.66 and 0.63 respectively); however, this was not significant. Free cholesterol was significantly associated with prediabetes but not T2D.

**Table 3:** Logistic regression of lipid classes against diabetes and prediabetes in the AusDiab cohort.

Lipid Class	Diabetes vs. NGT^a^			Prediabetes vs. NGT^b^		
	Odds Ratio^c^	p-value^d^	% difference^e^	Odds Ratio^d^	p-value^e^	% difference^b^
Dihydroceramide	1.90 (1.37–2.64)	**6.64E-04**	25.9	2.03 (1.42–2.91)	**1.07E-03**	24.5
Ceramide	1.70 (1.13–2.55)	**3.18E-02**	11.8	1.85 (1.16–2.95)	**2.43E-02**	13.9
Monohexosylceramide	1.04 (0.74–1.47)	9.20E-01	−0.3	1.33 (0.89–2.00)	2.57E-01	8.1
Dihexosylceramide	0.69 (0.48–1.00)	1.08E-01	−7.2	0.84 (0.56–1.26)	5.47E-01	−0.9
Trihexosylceramide	0.90 (0.60–1.34)	8.15E-01	−6.1	0.60 (0.37–0.99)	8.64E-02	−4.1
GM3 ganglioside	0.96 (0.65–1.42)	9.20E-01	−2.2	1.12 (0.71–1.76)	6.88E-01	5.5
Sphingomyelin	0.99 (0.65–1.49)	9.78E-01	0.9	1.18 (0.74–1.87)	6.35E-01	2.8
Phosphatidylcholine	1.12 (0.81–1.55)	7.10E-01	2.4	1.20 (0.84–1.70)	4.65E-01	3.0
Alkylphosphatidylcholine	0.67 (0.49–0.93)	**3.94E-02**	−3.8	0.62 (0.41–0.92)	**4.18E-02**	−3.2
Alkenylphosphatidylcholine	0.67 (0.45–1.01)	1.08E-01	−8.0	0.65 (0.39–1.06)	1.41E-01	−4.4
Lysophosphatidylcholine	1.00 (0.72–1.40)	9.78E-01	−2.6	1.52 (1.03–2.26)	7.46E-02	4.8
Lysoalkylphosphatidylcholine	0.74 (0.52–1.07)	1.81E-01	−8.3	1.00 (0.68–1.47)	9.94E-01	−0.8
Phosphatidylethanolamine	2.29 (1.51–3.48)	**6.64E-04**	31.3	2.01 (1.25–3.21)	**1.37E-02**	19.6
Phosphatidylinositol	1.67 (1.12–2.47)	**3.18E-02**	10.8	2.08 (1.26–3.43)	**1.37E-02**	11.1
Phosphatidylserine	1.21 (0.92–1.60)	2.78E-01	11.3	1.08 (0.83–1.42)	6.87E-01	4.1
Phosphatidylglycerol	1.85 (1.29–2.64)	**3.17E-03**	30.7	2.03 (1.34–3.08)	**5.72E-03**	25.7
Free cholesterol	1.07 (0.80–1.44)	8.17E-01	2.8	1.72 (1.17–2.53)	**1.65E-02**	17.0
Cholesterol ester	2.39 (1.67–3.41)	**2.89E-05**	32.7	1.85 (1.22–2.80)	**1.37E-02**	21.1
Diacylglycerol	2.98 (1.92–4.62)	**2.89E-05**	59.9	2.78 (1.81–4.28)	**7.39E-05**	49.4
Triacylglycerol	2.47 (1.68–3.62)	**4.46E-05**	53.0	2.79 (1.80–4.32)	**7.39E-05**	47.6

a Logistic regression of T2D (n = 116) against NGT (n = 168) on lipid classes adjusted for age, sex, waist circumference and SBP.

b Logistic regression of prediabetes (n = 64) against NGT (n = 168) on lipid classes adjusted for age, sex, waist circumference and SBP.

c Odds ratio (95% confidence intervals) based on an interquartile range increase in predictor lipid class measurement.

d Benjamini-Hochberg corrected p-value. Bold type indicates corrected p<0.05.

e % difference in means between groups.

Compared to NGT, 135 of the 259 lipid species were significantly associated with T2D, and 134 lipid species were significantly associated with prediabetes, after adjustment for age, sex, waist circumference and SBP (p<0.05, Tables S2 and S3 in File S1). Within each lipid class we observed considerable variation of the odds ratio of individual lipid species for each of the outcomes. Odds ratios of the cholesterol ester species for T2D ranged from 0.91 (95% CI: 0.69–1.21, p = 0.631) for CE 20∶1 to 2.88 (95% CI: 1.89–4.39, p = .94×10^−5^) for CE 16∶1. While sphingomyelin and phosphatidylcholine classes were not significantly associated with T2D, individual species containing odd chain (C15∶0 and C17∶0 fatty acids) showed contrasting associations with those containing even chain fatty acids. Five of the odd chain species showed significant negative associations with T2D (odds ratios 0.45–0.58). In contrast two of the even chain species showed significant positive associations (odds ratios of 1.68 and 1.84) (Table S3 in File S1). Odd chain fatty acids are primarily derived from ruminant fat with the primary source being ruminant fat, including dairy food; linear regression analysis of the 20 odd chain species in this study against full fat dairy intake, adjusted for age, sex, waist circumference and SBP, showed 12 to be significantly associated (after correction for multiple comparisons, p values ranging from 0.015 to 4.89×10^−5^) including SM 39∶1, PC 33∶2 and PC 35∶2 which were negatively associated with T2D.

### Association of lipids with fasting plasma glucose and 2h-post load glucose

**Figure 1:**
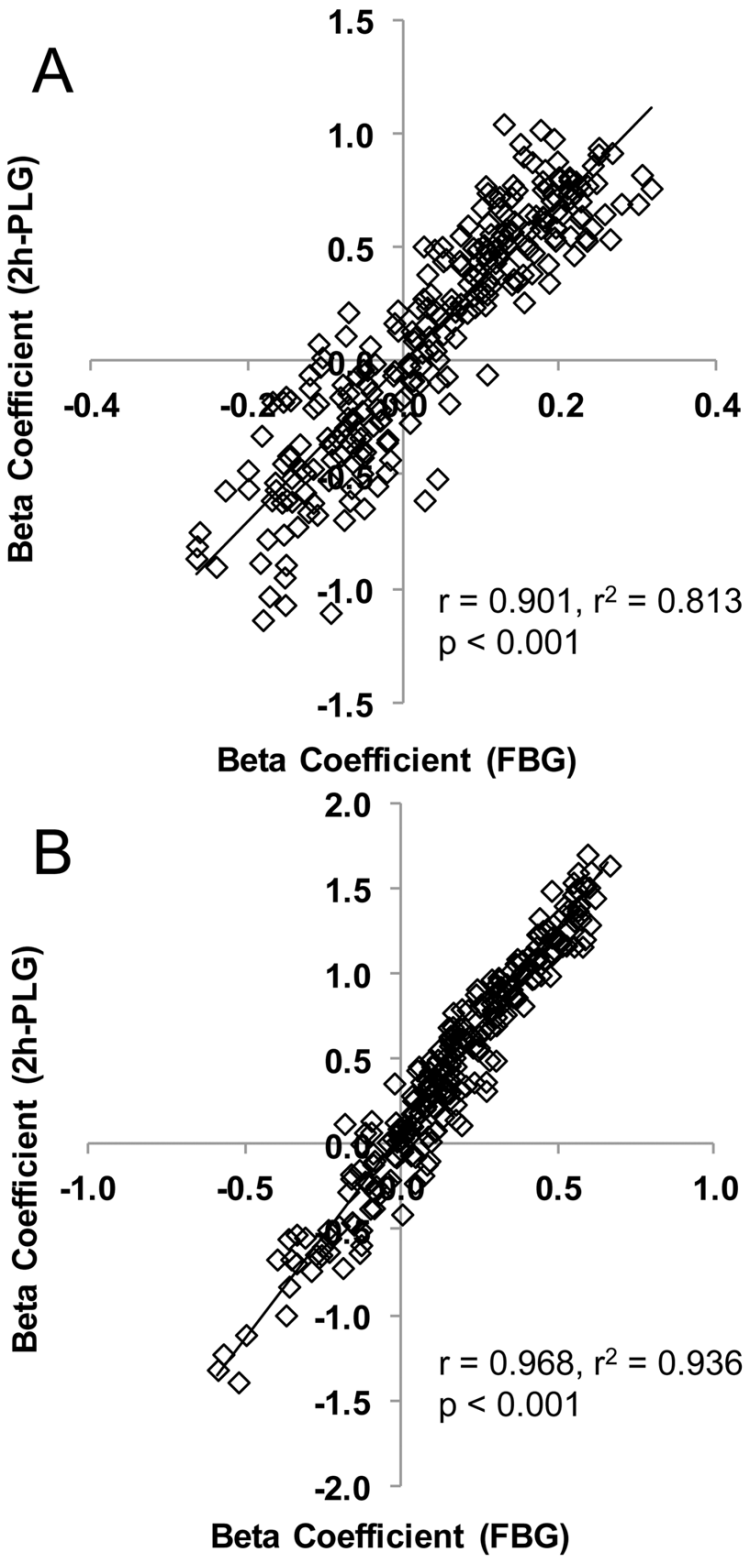
The relationship between beta-coefficients (FPG) and beta-coefficients (2h-PLG) in AusDiab and SAFHS. Linear regression of FPG and 2h-PLG on lipid species adjusted for age, sex, waist circumference and SBP were performed. Each data point represents a pair of the beta coefficients (FPG and 2h-PLG) for a single lipid species. Panel A – AusDiab; panel B – SAFHS.

As diabetes and prediabetes are based on arbitrary glucose cut-off points we also performed linear regression of lipids against FPG and 2h-post load glucose (2h-PLG), adjusting for age, sex, waist circumference and SBP. Dihydroceramide, phosphatidylethanolamine, phosphatidylglycerol, cholesterol ester, diacylglycerol and triacylglycerol lipid classes were all associated with FPG, while dihexosylceramide, lysoalkylphosphatidylcholine were negatively associated (Table 4). The same classes were also associated with 2h-PLG, or showed the same association. Interestingly the ether-linked lipids alkylphosphatidylcholine and alkenylphosphatidylcholine were also negatively associated with 2h-PLG (Table 4). There were 54 individual lipid species that were significantly associated with FPG, while 115 were significantly associated with 2h-PLG (Table S6 in File S1). Overall, the beta coefficients for FPG were highly correlated with the beta coefficients for 2h-PLG (r = 0.901, Figure 1A).

**Table 4:** Linear regression of lipid classes in the AusDiab and SAFHS cohorts.

	FPG^a^ (AusDiab)		2h-PLG^b^ (AusDiab)		FPG^a^ (SAFHS)		2h-PLG^b^ (SAFHS)	
Lipid Class	Beta Coeff.^c^	p-value^d^	Beta Coeff.^c^	p-value^d^	Beta Coeff.^c^	p-value^d^	Beta Coeff.^c^	p-value^d^
Dihydroceramide	0.19 (0.07–0.30)	**1.11E-02**	0.44 (0.07–0.82)	5.27E-02	0.57 (0.40–0.73)	**5.11E-10**	1.20 (0.86–1.53)	**2.09E-11**
Ceramide	0.06 (−0.09–0.22)	5.39E-01	0.31 (−0.18–0.80)	3.20E-01	0.54 (0.36–0.72)	**1.89E-08**	1.33 (0.98–1.68)	**1.82E-12**
Monohexosylceramide	−0.17 (−0.32–−0.02)	7.41E-02	−0.20 (−0.67–0.28)	4.55E-01	−0.09 (−0.27–0.09)	4.06E-01	−0.12 (−0.48–0.23)	5.70E-01
Dihexosylceramide	−0.22 (−0.37–−0.08)	**1.11E-02**	−0.58 (−1.03–−0.12)	**4.09E-02**	−0.26 (−0.44–−0.08)	**9.34E-03**	−0.57 (−0.93–−0.20)	**4.25E-03**
Trihexosylceramide	−0.11 (−0.28–0.06)	3.56E-01	−0.18 (−0.72–0.36)	5.41E-01	−0.19 (−0.36–−0.01)	5.67E-02	−0.43 (−0.78–−0.09)	**2.10E-02**
GM3 ganglioside	−0.06 (−0.23–0.11)	5.42E-01	−0.25 (−0.78–0.27)	4.02E-01	0.22 (0.04–0.41)	**2.94E-02**	0.46 (0.10–0.83)	**1.96E-02**
Sphingomyelin	−0.08 (−0.25–0.09)	4.86E-01	−0.33 (−0.86–0.20)	3.20E-01	−0.07 (−0.27–0.12)	5.24E-01	−0.12 (−0.51–0.27)	5.91E-01
Phosphatidylcholine	0.03 (−0.10–0.17)	6.75E-01	−0.27 (−0.69–0.15)	3.20E-01	0.16 (−0.03–0.35)	1.39E-01	0.64 (0.26–1.03)	**2.10E-03**
Alkylphosphatidylcholine	−0.08 (−0.22–0.05)	3.56E-01	−0.53 (−0.95–−0.11)	**4.09E-02**	−0.01 (−0.20–0.17)	9.37E-01	−0.30 (−0.67–0.07)	1.40E-01
Alkenylphosphatidylcholine	−0.11 (−0.28–0.06)	3.56E-01	−0.66 (−1.20–−0.13)	**4.09E-02**	−0.18 (−0.35–0.00)	6.75E-02	−0.69 (−1.03–−0.35)	**1.96E-04**
Lysophosphatidylcholine	−0.14 (−0.28–0.00)	1.24E-01	−0.34 (−0.78–0.10)	2.67E-01	0.02 (−0.16–0.19)	9.37E-01	−0.08 (−0.43–0.27)	6.53E-01
Lysoalkylphosphatidylcholine	−0.23 (−0.38–−0.09)	**1.11E-02**	−0.27 (−0.74–0.19)	3.47E-01	−0.36 (−0.54–−0.18)	**2.45E-04**	−0.79 (−1.15–−0.43)	**6.35E-05**
Phosphatidylethanolamine	0.24 (0.09–0.40)	**1.11E-02**	0.76 (0.28–1.24)	**1.16E-02**	0.62 (0.44–0.80)	**2.07E-10**	1.68 (1.33–2.03)	**5.42E-19**
Phosphatidylinositol	0.04 (−0.13–0.21)	6.75E-01	0.29 (−0.23–0.81)	3.57E-01	0.18 (−0.01–0.37)	9.50E-02	0.65 (0.27–1.03)	**1.63E-03**
Phosphatidylserine	0.05 (−0.06–0.16)	4.86E-01	0.29 (−0.06–0.64)	2.38E-01	0.00 (−0.14–0.15)	9.50E-01	0.09 (−0.21–0.39)	5.91E-01
Phosphatidylglycerol	0.20 (0.06–0.34)	**1.72E-02**	0.58 (0.14–1.01)	**4.09E-02**	0.43 (0.26–0.59)	**1.65E-06**	1.22 (0.89–1.54)	**2.58E-12**
Free cholesterol	−0.05 (−0.20–0.09)	5.42E-01	−0.22 (−0.66–0.23)	4.02E-01	0.13 (−0.05–0.31)	1.94E-01	0.32 (−0.04–0.68)	1.02E-01
Cholesterol ester	0.24 (0.11–0.37)	**5.71E-03**	0.82 (0.41–1.23)	**9.91E-04**	0.50 (0.33–0.67)	**8.99E-08**	1.22 (0.88–1.57)	**2.09E-11**
Diacylglycerol	0.19 (0.05–0.33)	**1.94E-02**	1.02 (0.60–1.44)	**6.06E-05**	0.57 (0.42–0.72)	**6.40E-12**	1.44 (1.14–1.74)	**4.51E-19**
Triacylglycerol	0.20 (0.07–0.32)	**1.11E-02**	0.73 (0.35–1.12)	**1.74E-03**	0.44 (0.27–0.60)	**8.18E-07**	1.26 (0.93–1.58)	**4.06E-13**

a Linear regression of FPG on lipid classes adjusted for age, sex, waist circumference and SBP.

b Linear regression of 2h-PLG on lipid classes adjusted for age, sex, waist circumference and SBP.

c Beta coefficient (95% confidence intervals) based on an interquartile range increase in predictor lipid class measurement.

d Benjamini-Hochberg corrected p-value. Bold type indicates corrected p<0.05.

### The relationship between type 2 diabetes and prediabetes

**Figure 2:**
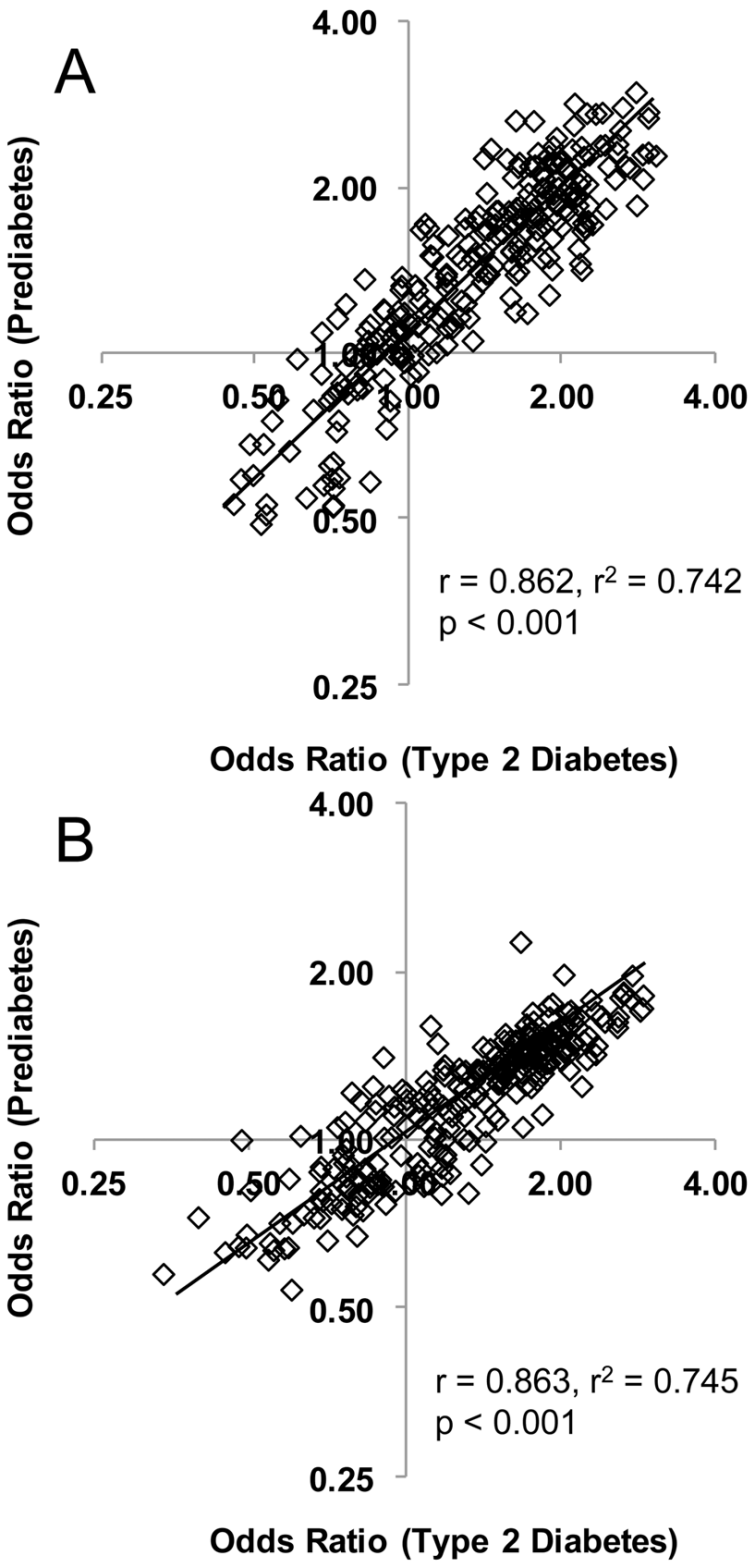
The relationship between odds ratios (T2D) and odds ratios (prediabetes) in AusDiab and SAFHS. Logistic regression of T2D and prediabetes on lipid species adjusted for age, sex, waist circumference and SBP were performed. Each data point represents a pair of the odds ratios (T2D and prediabetes) for a single lipid species. Panel A – AusDiab; panel B – SAFHS.

The lipid profile associated with prediabetes was strikingly similar to the lipid profile for T2D. All nine of the lipid classes associated with T2D (relative to NGT) were also associated with prediabetes (Table 3). Of the 135 lipid species associated with T2D, 111 were significantly associated with prediabetes and the remaining 24 showed non-significant associations in the same direction (Table S3 in File S1). There were no significant associations of lipid species or classes with T2D relative to prediabetes from adjusted (age, sex, waist circumference and SBP) binary logistic regression. The similarity in lipid profiles between T2D and prediabetes is further evidenced by a linear correlation coefficient of r = 0.862 between their odds ratios in the AusDiab cohort (Figure 2A).

### Validation of the AusDiab findings in the SAFHS

**Figure 3:**
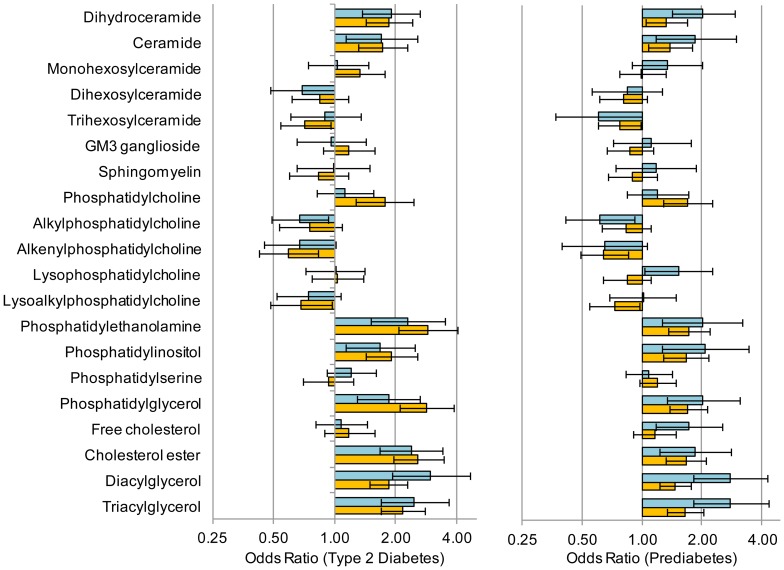
Associations of lipid classes with T2D and prediabetes in the AusDiab and SAHFS cohorts. Logistic regression of T2D and prediabetes on lipid species adjusted for age, sex, waist circumference and SBP were performed. Bars show the odds ratio for each lipid class, whiskers are the 95% confidence intervals. Panel A – T2D vs. NGT; panel B – prediabetes vs. NGT. Dark bars – AusDiab; light bars – SAFHS.

**Figure 4:**
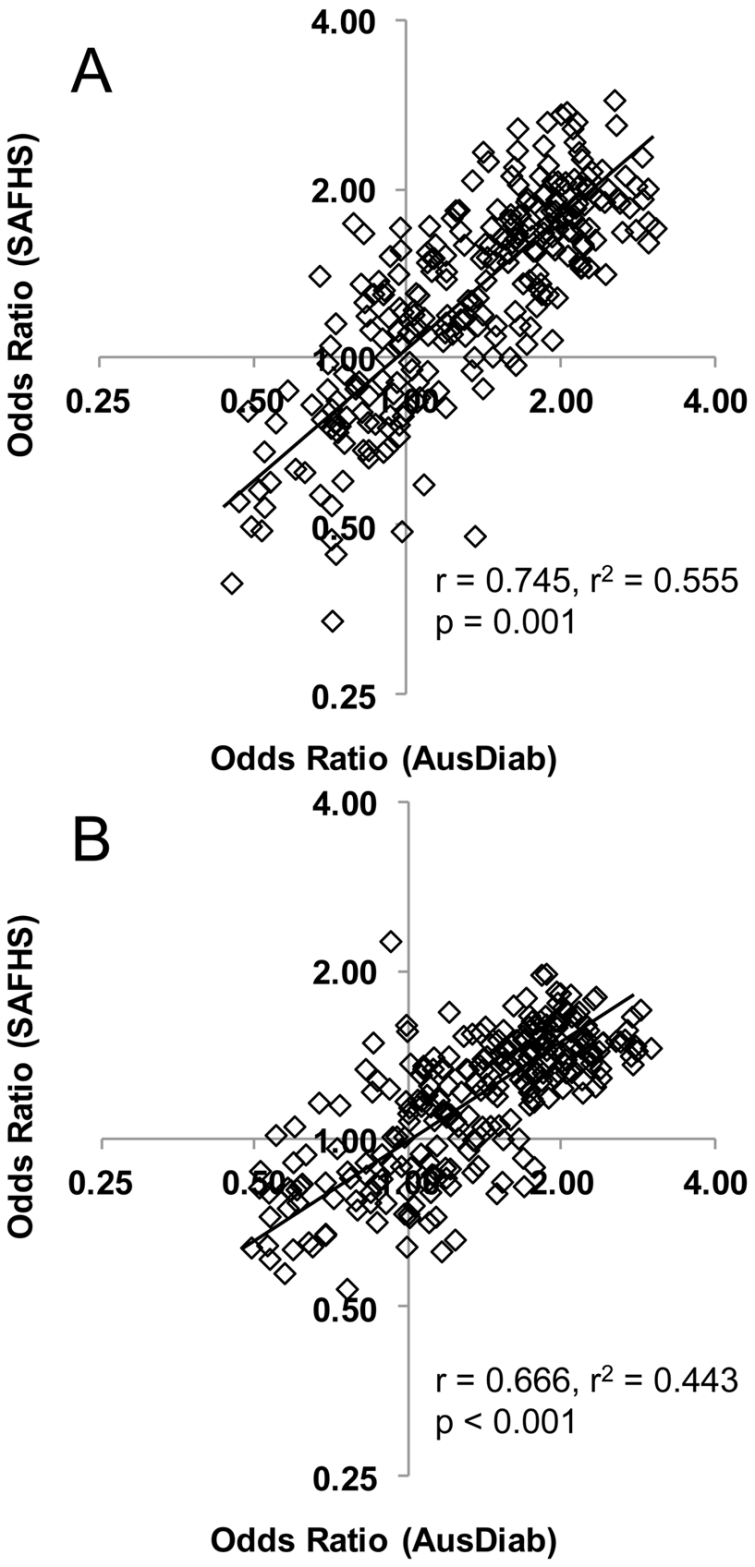
The relationship between AusDiab and SAFHS in the odds ratios (T2D) and odds ratios (prediabetes). Logistic regression of T2D and prediabetes on lipid species adjusted for age, sex, waist circumference and SBP were performed in the AusDiab and SAFHS cohorts. Each data point represents a pair of the odds ratios (AusDiab and SAFHS) for a single lipid species. Panel A – Odds ratios for T2D; panel B – Odds ratios for prediabetes.

Lipid profiling was performed on the SAFHS cohort and the same logistic regression analyses performed utilising the T2D (n = 69), prediabetes (n = 122) and the NGT (n = 796) who were not on any diabetes or lipid lowering medication. Eight out of the nine lipid classes that were significantly associated with T2D and prediabetes in AusDiab were also significantly associated with each outcome in SAFHS (Figure 3). Alkylphosphatidylcholine which showed a significant negative association with both T2D and prediabetes in the AusDiab cohort showed a non-significant negative association in the SAFHS cohort. Of the 135 individual lipid species that were significantly associated with T2D in the AusDiab cohort, 121 (90%) were also significantly associated (in the same direction) with T2D in the SAFHS cohort (Tables S2 and S5 in File S1). Similarly, the associations with prediabetes were confirmed in 104 of 134 lipid species (78%) (Tables S2 and S5 in File S1). Of the 14 species which did not show a significant association with T2D in the SAFHS two were odd chain lipids and seven were diacylglycerol and triacylglycerol species, notably containing one or more linoleic acid (C18∶2). No lipids showed contrasting significant associations between the two studies. A plot of the odds ratios for T2D in the AusDiab cohort against the SAFHS cohort showed a strong linear correlation (r = 0.775), while the correlation between the odds ratios for prediabetes between AusDiab and SAFHS was r = 0.666 (Figure 4). Linear regression against FPG and 2h-PLG also showed all of the same associations with lipid classes as seen in the AusDiab cohort (Table 4). The additional power of the larger SAFHS cohort also revealed associations of FPG with ceramide and G_M3_ ganglioside and of 2h-PLG with ceramide, phosphatidylcholine and phosphatidylinositol, while 2h-PLG was also negatively associated with trihexosylceramide and lysoalkylphosphatidylcholine but not with alkylphosphatidylcholine (Table 4). Of the 54 species associated with FPG in the AusDiab, 47 were also associated in the SAFHS. Similarly, of the 115 species associated with 2-PLG, 97 showed the same association in the SAFHS (Table S6 in File S1). As in the AusDiab cohort the beta coefficients for FPG and 2h-PLG in the SAFHS were highly correlated (r = 0.968, Figure 1B).

## Discussion

Our lipidomic analysis of a large case control cohort from the AusDiab study has identified a range of novel associations at both the lipid class and lipid species level. The plasma lipid profiles demonstrate that the differences in plasma lipids between T2D and NGT are clearly established in the prediabetes state.

Many lipidomic studies have been limited by small sample sizes resulting in a lack of statistical power and/or the inability to provide an independent validation of the findings. In this study we were able to utilise 351 participants of the AusDiab study to provide sufficient power to show significant associations with both prediabetes and T2D independent of each other and other covariates. Importantly, 90% of the lipids that were significantly associated with T2D in the AusDiab cohort were also significantly associated, and showed the same direction of association, with T2D in the larger population-based SAFHS cohort.

The initial cross-sectional case-control design from the AusDiab cohort in this study matched 117 cases (T2D) with 234 controls (NGT, IFG and IGT) for age, sex and waist circumference. Preliminary analyses identified that the plasma lipid profiles of the prediabetes individuals (IFG and IGT) were more similar to the T2D group than the NGT group. Stratification of the cohort into NGT and prediabetes groups resulted in significant differences in age and waist circumference requiring adjustment in the subsequent logistic regression analyses. Further to this, the SAFHS validation cohort (n = 1076) was limited by a number of factors; the SAFHS cohort had fewer males (39% vs. 50%), was substantially younger (median age of 35 vs. 62) and had lower blood pressure (median SBP of 117 vs. 137) compared to the AusDiab cohort. The SAFHS was a population study (as opposed to the AusDiab case/control study) and so had smaller proportions of T2D and prediabetes relative to NGT (n = 69, 122 and 796 respectively) not on diabetes lipid lowering medication. Nonetheless, most of the observed associations in the AusDiab cohort were confirmed in the SAFHS cohort in adjusted logistic regression. However, we note that the odds ratios were indicative of a greater effect size in the AusDiab cohort compared to the SAFHS cohort. This may relate to differences in the population structure (in terms of covariates) as outlined above but may also relate to the differences in the size and relative proportions of T2D and prediabetes (IGT and IFG) groups in relation to NGT group in each cohort. In contrast to the effect size, there were more lipids significant associated with T2D in the larger SAFHS cohort than the AusDiab cohort (135 vs. 166) with generally lower p-values. It is therefore likely that larger cohorts may identify additional lipids as significant, although these will likely have smaller effect sizes.

The prediabetes group consisted of both IFG and IGT which have been reported to have different etiologies with respect to basal insulin secretion and resistance of glucose production to suppression by insulin [17]. However, despite the different etiologies in these conditions we observed very similar associations between plasma lipids and FPG and plasma lipids and 2h-PLG (Figure 1) suggesting that the aberrant lipid metabolism associated with the prediabetes state is similar in both etiologies.

**Figure 5:**
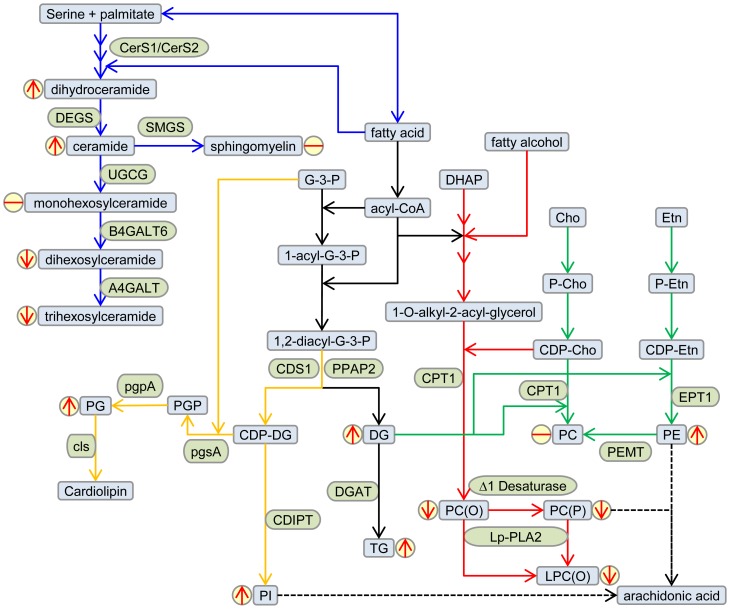
Metabolic pathways altered in type 2 diabetes. Partial lipid metabolic pathways show the major lipids associated with T2D (blue boxes) and the enzymes involved (green boxes). The sphingolipid (blue arrows), cardiolipin (orange arrows), triacylglycerol (black arrows), plasmalogen (red arrows) and phosphatidylcholine/phosphatidylethanolamine (green arrows) biosynthetic pathways are shown. The direction of association between lipids and T2D is indicated by the red arrows in yellow circles. Only partial pathways have been shown for clarity. **Metabolite abbreviations:** Cho, choline; DG, diacylglycerol; DHAP, dihydroxyacetonephosphate; Etn, ethanolamine; LPC(O), lysoalkylphosphatidylcholine; PC, phosphatidylcholine; PE, phosphatidylethanolamine; PC(O), alkylphosphatidylcholine; PC(P), alenylphosphatidylcholine; PG, phosphatidylglycerol; PGP, phosphatidylglycerolphosphate; PI, phosphatidylinositol; TG, triacylglycerol. **Enzyme abbreviations:** A4GALT, lactosylceramide 4-alpha-galactosyltransferase; B4GALT6, beta-1,4-galactosyltransferase 6; CDIPT, CDP-diacylglycerol-inositol 3-phosphatidyltransferase; CDS1, phosphatidate cytidylyltransferase; CerS, ceramide synthasecls, cardiolipin synthase; CPT1, diacylglycerol cholinephosphotransferase; DEGS, sphingolipid delta-4 desaturase; DGAT, diacylglycerol O-acyltransferase; EPT1, PEMT, phosphatidylethanolamine N-methyltransferase; ethanolaminephosphotransferase, pgpA, phosphatidylglycerophosphatase A; pgsA, CDP-diacylglycerol – glycerol-3-phosphate 3-phosphatidyltransferase; PPAP2, phosphatidate phosphatase; SMGS, sphingomyelin synthase; UGCG, ceramide glucosyltransferase.

The positive association of both ceramide and its biosynthetic precursor dihydroceramide with T2D and prediabetes (Figure 3) firmly places the ceramide biosynthetic pathway as a key metabolic process in T2D. This was validated in the SAFHS cohort, although the association of dihydroceramide with prediabetes was not significant after correcting for multiple comparisons (Table S4 in File S1). The apparent upregulation of *de novo* ceramide synthesis did not flow through to other ceramide metabolites such as sphingomyelin and the glysosphingolipids, in fact dihexosylceramide showed a negative association with T2D in the AusDiab cohort while we also observed a significant negative association with trihexosylceramide in the SAFHS cohort (Figure 5, Table 3, Table S4 in File S1).

When we examined the association with the individual ceramide species and their precursors dihydroceramide, we observed that, despite being one of the least abundant dihydroceramide species, dhCer 18∶0 showed the strongest association with both T2D (IQR odds ratio = 2.95, 95% CI: 1.93–4.50, p = 5.23×10^−5^) and prediabetes (IQR odds ratio  = 2.32, 95% CI: 1.57–3.42, p = 3.75×10^−4^) (Table S3 in File S1), and the corresponding ceramide species also showed the strongest associations. Ceramide synthase 1 (CerS 1) is reported to be specific for the synthesis of dhCer 18∶0/Cer 18∶0 [18] and its expression in muscle has been shown to be inversely associated with alterations in glucose tolerance in a mouse model of insulin resistance [19]. CerS 4 also shows specificity for C18∶0 and C20∶0 acyl chains but is primarily expressed in kidney, heart, spleen, and lung with only weak expression in liver and muscle [18]. CerS 2 is the predominant form in liver and shows specificity for the longer chain acyl species C20∶0, C22∶0, C24∶0 and C24∶1. Interestingly Cer 16∶0 did not show a significant association with either T2D or prediabetes and dhCer 16∶0 was only significantly associated with T2D. CerS 5 and CerS 6 are reported to have specificity for the C16∶0 acyl chain and are expressed primarily in lung and intestine/kidney respectively [20], [21]. Taken together, these results suggest an up regulation of ceramide synthesis, most likely involving CerS 1 in muscle and CerS 2 in liver. This may be further exacerbated by a down regulation of the glycosyltransferases responsible for the conversion of ceramide to dihexosylceramide and trihexosylceramide.

Although there was no significant difference between the clinical measure of total cholesterol in the NGT, T2D and prediabetes groups, the total cholesterol ester and most cholesterol ester species (determined by mass spectrometry) were strongly associated with T2D and prediabetes after adjusting for age, sex, waist circumference and SBP. This likely relates to the higher triglyceridemia and increased VLDL production associated with prediabetes and T2D [22]. Phosphatidylethanolamine, phosphatidylinositol and phosphatidylglycerol, but not phosphatidylcholine, also showed positive associations with T2D and prediabetes (Figure 5). While these associations may also relate to increased lipoprotein production, the respective roles of these lipids as a source of arachidonic acid for the production of proinflammatory eicosinoids (phosphatidylethanolamine and phosphatidylinositol) and as a substrate for the production for the mitochondrial specific lipid cardiolipin (phosphatidylglycerol) provide future avenues for investigation. A recent study has also identified phosphatidylethanolamine as a positive modulator of the membrane disruption induced by IAPP (islet amyloid polypeptide protein), an amyloidogenic protein involved in type II diabetes [23].

Of interest was the negative association of some phosphatidylcholine and sphingomyelin species containing odd chain fatty acids (C15∶0 and C17∶0) with prediabetes and T2D (Table S3 in File S1). These odd chain fatty acids are products of ruminant digestion and in human diets are derived primarily from dairy fats; a number of studies have validated the association between odd chain fatty acids and dairy intake [24], [25]. This would support a protective role for dairy against T2D and is in agreement with a mounting body of epidemiological data [26], [27], [28] as well as specific measurement of fatty acid composition that have demonstrated negative associations between C15∶0 and C17∶0 with incident T2D [29], [30] and a recent study of Mozaffarian *et al*. who reported a negative association between the dairy derived fatty acid, *trans-*palmitoleate, and incidence of T2D [31].

Negative associations with prediabetes and T2D were also observed for the ether and vinyl ether-linked phosphatidylcholine species alkylphosphatidylcholine and alkenylphosphatidylcholine. These associations were species specific (Table S3 in File S1) suggesting a complex relationship with different stages of disease. The vinyl ether linkage of the plasmalogens is particularly susceptible to oxidation by reactive oxygen species [32], [33] and as such they are proposed to play an antioxidant role in membranes and lipoproteins [34], [35], While the ether linkage of the alkylphosphatidylcholine species is not susceptible to oxidation the high proportion of polyunsaturated fatty acids in these lipids render them also susceptible to oxidative modification. Alternatively, decreased levels of these lipids in plasma may relate to a decreased biosynthesis which is thought to be controlled by the formation and availability of the long chain fatty alcohol used to produce the 1-O-alky-dihydroxyacetonephosphate in the peroxisome, an early process in plasmalogens biosynthesis [35]. Likely, it is a combination of effects controlling plasma levels and further work will be required to fully understand the drivers and consequences of alkyl- and alkenylphosphatidylcholine metabolism in T2D.

All three species of lysoalkylphosphatidylcholine [LPC(O-22∶0), LPC(O-24∶1) and LPC(O-24∶2)] were also negatively associated with T2D (Figure 5, Table S3 in File S1). This lipid, also known as lysoplatelet activating factor, is formed by the action of platelet activating factor acetylhydrolase (PAF-AH) also known as lipoprotein phospholipase A2 (Lp-PLA2) on platelet activating factor (PAF). PAF is a proinflammatory signalling lipid that is controlled by the concerted action of the biosynthetic enzyme lysophosphatidylcholine acyltransferase 2 (LPCAT2) and the catabolic action of PAF-AH/Lp-PLA2. While PAF-AH/Lp-PLA2 has been reported to be elevated in T2D [36], [37] its distribution between LDL and HDL is altered in T2D [38] and may be a critical factor in controlling the level of PAF/lyso-PAF as well as other oxidised species of phospholipid. Fan *et al*. investigated the HDL associated PAF-AH/Lp-PLA2 in polycystic ovary syndrome patients and observed lower PAF-AH/Lp-PLA2 in affected patients, which was associated with insulin resistance [39]. Thus PAF metabolism and/or metabolism of oxidised phosphatidylcholine species may be important processes in the progression to T2D.

Many of the alterations in lipid metabolic pathways identified in this study are linked to free fatty acids (Figure 5) which highlights the central role of elevated free fatty acids in the dysregulation of lipid homeostasis associated with T2D. The driving force of the elevated free fatty acids combined with differential regulation of metabolic pathways as well as spatial effects resulting from tissue and or plasma compartmentalisation, leads to a complex metabolic phenotype. Lipidomic studies can provide new insight for existing paradigms of lipid dysregulation, and identify new avenues to investigate the complex relationship between lipid metabolism, plasma lipids and T2D.

## Supporting Information

File S1 **Contains: Table S1.** Conditions for precursor ion scan and MRM acquisition methods for lipid identification and analysis. **Table S2**. Lipid species associated with diabetes and prediabetes in the AusDiab and SAFHS cohorts. **Table S3**. Logistic regression of lipids against diabetes and prediabetes in the AusDiab cohort. **Table S4**. Logistic regression of lipid classes in the SAFHS cohort. **Table S5**. Logistic regression of lipids against diabetes and prediabetes in the SAFHS cohort. **Table S6**. Linear regression of lipid species in the AusDiab and SAFHS cohorts.
(DOCX)
