# Supplementary material for: Plasma Lipid Profiling Shows Similar Associations with Prediabetes and Type 2 Diabetes
Source: PLoS One. 2013 Sep 27;8(9):e74341. doi: 10.1371/journal.pone.0074341 (PMC3785490; doi:10.1371/journal.pone.0074341)
Supplement: File S1 — Contains: Table S1. Conditions for precursor ion scan and MRM acquisition methods for lipid identification and analysis. Table S2. Lipid species associated with diabetes and prediabetes in the AusDiab and SAFHS cohorts. Table S3. Logistic regression of lipids against diabetes and prediabetes in the AusDiab cohort. Table S4. Logistic regression of lipid classes in the SAFHS cohort. Table S5. Logistic regression of lipids against diabetes and prediabetes in the SAFHS cohort. Table S6. Linear regression of lipid species in the AusDiab and SAFHS cohorts. (DOCX) [file pone.0074341.s001.docx]

**Online Supplemental Material**

**Plasma Lipid Profiling Shows Similar Associations with Prediabetes and Type 2 Diabetes**

Peter J Meikle^a^, Gerard Wong^a^, Christopher K Barlow^a^, Jacquelyn M Weir^a^, Melissa A. Greeve^a^, Gemma L MacIntosh^a^, Laura Almasy^b^, Anthony G Comuzzie^b^, Michael C Mahaney^b^, Adam Kowalczyk^c^, Izhac Haviv^a^, Narelle Grantham^a^, Dianna J Magliano^a^, Jeremy B M Jowett^a^, Paul Zimmet^a^, Joanne E Curran^b^, John Blangero^b^, Jonathan Shaw^a^.

^a^ Baker IDI Heart and Diabetes Institute, Melbourne, Victoria, Australia. ^b^ Department of Genetics, Texas Biomedical Research Institute, San Antonio, Texas, USA. ^c^ National ICT Australia (NICTA), University of Melbourne, Melbourne, Victoria, Australia.

**Address for correspondence:** Peter J Meikle, Baker IDI Heart and Diabetes Institute, 75 Commercial Road, Melbourne, Victoria 3004, Australia. Tel: (613) 8532 1770, Fax: (613) 8532 1100, Email: peter.meikle@bakeridi.edu.au

**Table S1. Conditions for precursor ion scan and MRM acquisition methods for lipid identification and analysis.**

| **Lipid class** | **No. Of species** | **Internal standard** | **pmol/ 15µL** | **Parent ion** | **Experiment** | **DP**^a^ | **EP**^b^ | **CEn**^c^ | **CXP**^d^ |
| --- | --- | --- | --- | --- | --- | --- | --- | --- | --- |
| Dihydroceramide (dhCer) | 6 | dhCer 8:0 | 100 | [M+H]^+^ | PIS^e^, 284.3 *m/z* | 90 | 10 | 39 | 18 |
| Ceramide (Cer) | 6 | Cer 17:0 | 100 | [M+H]^+^ | PIS, 264.3 *m/z* | 50 | 10 | 35 | 12 |
| Monohexosylceramide (MHC) | 6 | MHC 16:0 *d_3_* | 50 | [M+H]^+^ | PIS, 264.3 *m/z* | 77 | 10 | 50 | 12 |
| Dihexosylceramide (DHC) | 6 | DHC 16:0 *d_3_* | 50 | [M+H]^+^ | PIS, 264.3 *m/z* | 100 | 10 | 65 | 12 |
| Trihexosylcermide (THC) | 6 | THC 17:0 | 50 | [M+H]^+^ | PIS, 264.3 *m/z* | 130 | 10 | 73 | 12 |
| G_M3_ ganglioside (GM3) | 6 | THC 17:0 | 50 | [M+H]^+^ | PIS, 264.3 *m/z* | 155 | 10 | 105 | 16 |
| Sphingomyelin (SM) | 13 | SM 12:0 | 200 | [M+H]^+^ | PIS, 184.1 *m/z* | 65 | 10 | 35 | 12 |
| Phosphatidylcholine (PC) | 26 | PC 13:0/13:0 | 100 | [M+H]^+^ | PIS, 184.1 *m/z* | 100 | 10 | 45 | 11 |
| Alkylphosphatidylcholine (PC(O)) | 16 | PC 13:0/13:0 | 100 | [M+H]^+^ | PIS, 184.1 *m/z* | 100 | 10 | 45 | 11 |
| Alkenylphosphatidylcholine (PC(P)) | 8 | PC 13:0/13:0 | 100 | [M+H]^+^ | PIS, 184.1 *m/z* | 100 | 10 | 45 | 11 |
| Lysophosphatidylcholine (LPC) | 16 | LPC 13:0 | 100 | [M+H]^+^ | PIS, 184.1 *m/z* | 90 | 10 | 38 | 12 |
| Lysoalkylphosphatidylcholine (LPC(O)) | 6 | LPC 13:0 | 100 | [M+H]^+^ | PIS, 285.2 *m/z* | 90 | 10 | 42 | 5 |
| Phosphatidylethanolamine (PE) | 18 | PE 17:0/17:0 | 100 | [M+H]^+^ | NL^f^, 141 Da | 80 | 10 | 31 | 12 |
| Phosphatidylinositol (PI) | 17 | PE 17:0/17:0 | 100 | [M^+^ NH_4_]^+^ | PIS, 184.1 *m/z* | 51 | 10 | 43 | 14 |
| Phosphatidylserine (PS) | 11 | PS 17:0/17:0 | 100 | [M+H]^+^ | NL, 185Da | 86 | 10 | 29 | 12 |
| Phosphatidylglycerol (PG) | 4 | PG 17:0/17:0 | 100 | [M^+^ NH_4_]^+^ | NL, 189 Da | 60 | 10 | 25 | 12 |
| Free cholesterol (COH) | 1 | COH *d_7_* | 1000 | [M^+^ NH_4_]^+^ | PIS, 369.3 *m/z* | 55 | 10 | 17 | 12 |
| Cholesterol ester (CE) | 31 | CE 18:0 *d_6_* | 1000 | [M^+^ NH_4_]^+^ | PIS, 369.3 *m/z* | 30 | 10 | 20 | 12 |
| Diacylglycerol (DG) | 27 | DG 15:0/15:0 | 200 | [M^+^ NH_4_]^+^ | NL, fatty acid | 55 | 10 | 30 | 22 |
| Triaclyglycerol (TG) | 44 | TG 17:0/17:0/17:0 | 100 | [M^+^ NH_4_]^+^ | NL, fatty acid | 95 | 10 | 30 | 12 |

^a^ DP, declustering potential; ^b^ EP, entrance potential; ^c^ CEn, collision energy; ^d^ CXP, exit potential; ^e^ PIS, precursor ion scan; ^f^ NL, neutral loss scan.

**Table S2. Lipid species associated with diabetes and prediabetes in the AusDiab and SAFHS cohorts.**

|  |  | | **# of individual species** | | |  |
| --- | --- | --- | --- | --- | --- | --- |
| **Lipid class** | **total^a^** | **Diabetes vs. NGT^b^** | | | **Prediabetes vs. NGT^c^** | |
|  |  | **AusDiab** | | **SAFHS** | **AusDiab** | **SAFHS** |
| Dihydroceramide | 6 | 5 | | 5 | 5 | 2 |
| Ceramide | 6 | 4 | | 6 | 5 | 5 |
| Monohexosylceramide | 6 | 0 | | 2 | 0 | 0 |
| Dihexosylceramide | 6 | 1 | | 2 | 0 | 0 |
| Trihexosylceramide | 6 | 0 | | 1 | 1 | 0 |
| GM3 ganglioside | 6 | 0 | | 2 | 0 | 0 |
| Sphingomyelin | 12 | 2 | | 3 | 1 | 1 |
| Phosphatidylcholine | 38 | 5 | | 18 | 6 | 17 |
| Alkylphosphatidylcholine | 15 | 3 | | 6 | 9 | 3 |
| Alkenylphosphatidylcholine | 5 | 1 | | 3 | 2 | 3 |
| Lysophosphatidylcholine | 16 | 1 | | 6 | 5 | 6 |
| Lysoalkylphosphatidylcholine | 3 | 3 | | 2 | 0 | 1 |
| Phosphatidylethanolamine | 14 | 14 | | 14 | 7 | 13 |
| Phosphatidylinositol | 17 | 9 | | 14 | 10 | 15 |
| Phosphatidylserine | 7 | 1 | | 0 | 0 | 0 |
| Phosphatidylglycerol | 4 | 2 | | 4 | 3 | 4 |
| Free cholesterol | 1 | 0 | | 0 | 1 | 0 |
| Cholesterol ester | 26 | 20 | | 20 | 14 | 17 |
| Diacylglycerol | 22 | 22 | | 21 | 22 | 21 |
| Triacylglycerol | 43 | 42 | | 37 | 43 | 38 |
| **total** | **259** | **135** | | **166** | **134** | **146** |

^a^ The number of lipid species in each class.

^b^ The number of lipid species in each class with p < 0.05 (Benjamini-Hochberg corrected) based on logistic regression of T2D against NGT adjusted for age, sex, waist circumference and SBP.

^c^ The number of lipid species in each class with p < 0.05 (Benjamini-Hochberg corrected) based on logistic regression of prediabetes against NGT adjusted for age, sex, waist circumference and SBP.

**Table S3. Logistic regression of lipids against diabetes and prediabetes in the AusDiab cohort.**

|  |  | **Diabetes vs. NGT^a^** | | |  | **Prediabetes vs. NGT^b^** | | |
| --- | --- | --- | --- | --- | --- | --- | --- | --- |
| **Lipid Species** |  | **Odds Ratio^c^** | **p-value^d^** | **%**  **difference^e^** |  | **Odds Ratio^c^** | **p-value^d^** | **%**  **difference^e^** |
| dhCer 16:0 |  | 1.62 (1.15-2.28) | **1.36E-02** | 13.7 |  | 1.39 (0.95-2.03) | 1.43E-01 | 10.5 |
| dhCer 18:0 |  | 2.95 (1.93-4.50) | **5.23E-05** | 35.4 |  | 2.32 (1.57-3.42) | **3.75E-04** | 29.6 |
| dhCer 20:0 |  | 1.50 (1.05-2.14) | 5.45E-02 | 15.6 |  | 1.82 (1.21-2.73) | **1.25E-02** | 16.6 |
| dhCer 22:0 |  | 2.03 (1.41-2.94) | **9.32E-04** | 29.5 |  | 2.18 (1.48-3.21) | **7.09E-04** | 26.9 |
| dhCer 24:0 |  | 1.63 (1.19-2.23) | **6.68E-03** | 27.4 |  | 1.72 (1.24-2.38) | **4.91E-03** | 25.6 |
| dhCer 24:1 |  | 1.84 (1.31-2.59) | **1.90E-03** | 23.8 |  | 2.08 (1.42-3.06) | **1.29E-03** | 26.6 |
| Cer 16:0 |  | 1.34 (0.93-1.92) | 1.89E-01 | 6.1 |  | 1.29 (0.84-1.99) | 3.43E-01 | 7.9 |
| Cer 18:0 |  | 2.14 (1.46-3.15) | **6.85E-04** | 23.9 |  | 1.86 (1.24-2.80) | **1.04E-02** | 18.7 |
| Cer 20:0 |  | 1.78 (1.23-2.59) | **6.83E-03** | 16.0 |  | 1.68 (1.13-2.48) | **2.47E-02** | 15.1 |
| Cer 22:0 |  | 1.94 (1.32-2.84) | **2.74E-03** | 18.1 |  | 1.74 (1.13-2.69) | **2.97E-02** | 14.8 |
| Cer 24:0 |  | 1.43 (0.98-2.09) | 1.16E-01 | 10.0 |  | 1.68 (1.08-2.60) | **4.56E-02** | 13.8 |
| Cer 24:1 |  | 1.53 (1.08-2.15) | **3.32E-02** | 11.6 |  | 1.66 (1.07-2.56) | **4.89E-02** | 14.6 |
| MHC 16:0 |  | 0.96 (0.67-1.38) | 8.73E-01 | -0.2 |  | 1.31 (0.88-1.95) | 2.81E-01 | 8.9 |
| MHC 18:0 |  | 1.03 (0.73-1.45) | 9.00E-01 | 0.7 |  | 1.33 (0.89-1.99) | 2.48E-01 | 9.4 |
| MHC 20:0 |  | 1.19 (0.83-1.70) | 4.40E-01 | 2.0 |  | 1.35 (0.89-2.03) | 2.39E-01 | 8.3 |
| MHC 22:0 |  | 1.05 (0.74-1.49) | 8.59E-01 | 0.4 |  | 1.25 (0.84-1.84) | 3.71E-01 | 7.4 |
| MHC 24:0 |  | 1.11 (0.78-1.58) | 6.43E-01 | 1.2 |  | 1.58 (0.99-2.52) | 9.92E-02 | 9.8 |
| MHC 24:1 |  | 0.96 (0.69-1.33) | 8.72E-01 | -3.5 |  | 1.11 (0.80-1.54) | 6.52E-01 | 5.7 |
| DHC 16:0 |  | 0.71 (0.49-1.01) | 1.04E-01 | -7.3 |  | 0.84 (0.56-1.25) | 5.05E-01 | -1.3 |
| DHC 18:0 |  | 0.67 (0.46-0.98) | 7.19E-02 | -5.8 |  | 0.92 (0.62-1.36) | 7.58E-01 | 2.6 |
| DHC 20:0 |  | 0.82 (0.58-1.17) | 3.70E-01 | -4.1 |  | 1.10 (0.72-1.68) | 7.46E-01 | 6.7 |
| DHC 22:0 |  | 0.86 (0.61-1.20) | 4.69E-01 | -3.9 |  | 1.06 (0.73-1.53) | 8.38E-01 | 3.8 |
| DHC 24:0 |  | 1.09 (0.78-1.52) | 6.99E-01 | 0.3 |  | 1.00 (0.68-1.46) | 9.95E-01 | 2.2 |
| DHC 24:1 |  | 0.65 (0.45-0.94) | **4.58E-02** | -10.0 |  | 0.79 (0.53-1.19) | 3.64E-01 | -2.4 |
| THC 16:0 |  | 0.89 (0.60-1.33) | 6.80E-01 | -5.6 |  | 0.71 (0.44-1.16) | 2.52E-01 | -2.0 |
| THC 18:0 |  | 0.99 (0.69-1.42) | 9.55E-01 | -2.3 |  | 0.77 (0.50-1.18) | 3.21E-01 | -1.6 |
| THC 20:0 |  | 0.84 (0.57-1.23) | 4.61E-01 | -8.6 |  | 0.68 (0.43-1.08) | 1.67E-01 | -7.9 |
| THC 22:0 |  | 0.84 (0.58-1.20) | 4.21E-01 | -9.3 |  | 0.51 (0.32-0.81) | **1.51E-02** | -11.1 |
| THC 24:0 |  | 1.05 (0.71-1.53) | 8.72E-01 | -4.9 |  | 0.61 (0.38-0.98) | 7.51E-02 | -7.1 |
| THC 24:1 |  | 0.91 (0.62-1.34) | 7.22E-01 | -7.3 |  | 0.58 (0.35-0.96) | 6.63E-02 | -5.4 |
| GM3 16:0 |  | 0.79 (0.53-1.19) | 3.60E-01 | -4.1 |  | 0.88 (0.55-1.43) | 7.16E-01 | 2.5 |
| GM3 18:0 |  | 0.81 (0.58-1.14) | 3.32E-01 | -5.1 |  | 1.02 (0.69-1.50) | 9.59E-01 | 4.3 |
| GM3 20:0 |  | 1.11 (0.77-1.62) | 6.76E-01 | 0.5 |  | 1.50 (0.95-2.36) | 1.38E-01 | 9.2 |
| GM3 22:0 |  | 1.27 (0.88-1.82) | 2.90E-01 | 4.6 |  | 1.25 (0.83-1.88) | 4.01E-01 | 8.1 |
| GM3 24:0 |  | 0.89 (0.62-1.29) | 6.42E-01 | -4.7 |  | 1.20 (0.79-1.83) | 5.05E-01 | 7.7 |
| GM3 24:1 |  | 1.09 (0.77-1.56) | 7.04E-01 | -1.8 |  | 1.08 (0.71-1.64) | 7.90E-01 | 5.6 |
| SM 32:1 |  | 0.96 (0.72-1.27) | 8.51E-01 | 2.1 |  | 1.17 (0.80-1.71) | 5.52E-01 | 8.5 |
| SM 33:1 |  | 0.79 (0.57-1.10) | 2.55E-01 | -3.9 |  | 0.86 (0.56-1.31) | 6.01E-01 | 0.8 |
| SM 34:1 |  | 0.96 (0.68-1.36) | 8.72E-01 | -0.9 |  | 1.09 (0.72-1.66) | 7.58E-01 | 3.1 |
| SM 34:2 |  | 0.75 (0.50-1.13) | 2.55E-01 | -2.4 |  | 0.95 (0.57-1.56) | 8.84E-01 | 2.7 |
| SM 36:1 |  | 1.27 (0.86-1.87) | 3.32E-01 | 5.5 |  | 1.48 (0.96-2.29) | 1.30E-01 | 7.4 |
| SM 36:2 |  | 0.96 (0.66-1.41) | 8.73E-01 | 1.7 |  | 1.01 (0.62-1.63) | 9.94E-01 | 2.7 |
| SM 37:2 |  | 0.45 (0.30-0.69) | **1.30E-03** | -14.6 |  | 0.53 (0.35-0.81) | **1.21E-02** | -10.4 |
| SM 38:1 |  | 0.86 (0.61-1.22) | 5.07E-01 | -1.0 |  | 1.00 (0.68-1.48) | 9.99E-01 | 1.3 |
| SM 38:2 |  | 1.35 (0.91-2.02) | 2.19E-01 | 8.9 |  | 1.53 (1.02-2.30) | 7.97E-02 | 11.5 |
| SM 39:1 |  | 0.49 (0.33-0.72) | **1.31E-03** | -10.7 |  | 0.68 (0.47-1.00) | 9.36E-02 | -3.7 |
| SM 41:1 |  | 1.03 (0.69-1.52) | 9.12E-01 | 1.8 |  | 1.05 (0.66-1.68) | 8.84E-01 | 1.7 |
| SM 42:1 |  | 1.37 (0.96-1.95) | 1.43E-01 | 5.9 |  | 1.41 (0.94-2.12) | 1.60E-01 | 6.7 |
| PC 28:0 |  | 0.98 (0.74-1.29) | 9.00E-01 | 3.1 |  | 1.19 (0.93-1.51) | 2.48E-01 | 36.7 |
| PC 29:0 |  | 0.72 (0.50-1.04) | 1.40E-01 | -6.0 |  | 0.85 (0.55-1.30) | 5.74E-01 | 1.7 |
| PC 31:0 |  | 0.92 (0.66-1.29) | 7.17E-01 | 0.9 |  | 1.07 (0.72-1.57) | 8.15E-01 | 4.1 |
| PC 31:1 |  | 0.74 (0.51-1.06) | 1.62E-01 | -4.0 |  | 0.88 (0.57-1.37) | 6.88E-01 | 1.4 |
| PC 32:0 |  | 1.26 (0.91-1.74) | 2.52E-01 | 3.2 |  | 1.17 (0.79-1.71) | 5.54E-01 | 3.6 |
| PC 32:1 |  | 1.84 (1.25-2.71) | **5.86E-03** | 16.7 |  | 1.72 (1.15-2.57) | **2.27E-02** | 13.3 |
| PC 32:2 |  | 0.97 (0.67-1.39) | 8.90E-01 | 2.8 |  | 1.38 (0.89-2.14) | 2.37E-01 | 7.8 |
| PC 33:0 |  | 0.73 (0.52-1.02) | 1.12E-01 | -4.7 |  | 0.76 (0.49-1.17) | 3.05E-01 | -1.4 |
| PC 33:1 |  | 0.84 (0.60-1.18) | 4.17E-01 | -1.0 |  | 1.00 (0.65-1.55) | 9.99E-01 | 2.0 |
| PC 33:2 |  | 0.58 (0.38-0.88) | **2.39E-02** | -7.4 |  | 0.67 (0.42-1.06) | 1.41E-01 | -4.1 |
| PC 34:0 |  | 0.89 (0.66-1.21) | 5.81E-01 | -2.0 |  | 1.19 (0.83-1.71) | 4.51E-01 | 5.2 |
| PC 34:1 |  | 1.22 (0.87-1.70) | 3.45E-01 | 3.4 |  | 1.12 (0.80-1.56) | 6.32E-01 | 2.8 |
| PC 34:2 |  | 0.81 (0.57-1.14) | 3.26E-01 | -0.9 |  | 0.87 (0.59-1.27) | 5.74E-01 | -0.2 |
| PC 34:3 |  | 1.10 (0.74-1.64) | 7.18E-01 | 4.0 |  | 1.51 (0.97-2.34) | 1.16E-01 | 9.1 |
| PC 34:5 |  | 1.18 (0.88-1.60) | 3.68E-01 | 11.4 |  | 1.40 (1.06-1.84) | **3.73E-02** | 26.7 |
| PC 35:0 |  | 0.98 (0.80-1.20) | 8.73E-01 | -0.7 |  | 0.96 (0.72-1.30) | 8.75E-01 | 1.3 |
| PC 35:1 |  | 0.70 (0.48-1.00) | 9.52E-02 | -4.8 |  | 0.82 (0.51-1.32) | 5.35E-01 | -0.6 |
| PC 35:2 |  | 0.47 (0.31-0.70) | **1.08E-03** | -11.0 |  | 0.59 (0.39-0.89) | **3.08E-02** | -5.2 |
| PC 35:3 |  | 0.76 (0.52-1.10) | 2.28E-01 | -4.0 |  | 0.85 (0.50-1.44) | 6.49E-01 | -0.2 |
| PC 35:4 |  | 0.92 (0.70-1.21) | 6.42E-01 | -2.3 |  | 0.82 (0.53-1.27) | 4.96E-01 | -2.7 |
| PC 36:1 |  | 0.78 (0.54-1.13) | 2.76E-01 | -1.3 |  | 0.99 (0.67-1.48) | 9.90E-01 | 0.7 |
| PC 36:2 |  | 0.83 (0.59-1.17) | 3.77E-01 | -0.9 |  | 1.06 (0.69-1.62) | 8.72E-01 | 2.1 |
| PC 36:3 |  | 0.96 (0.67-1.36) | 8.65E-01 | 1.7 |  | 0.99 (0.66-1.48) | 9.83E-01 | 1.1 |
| PC 36:4 |  | 0.80 (0.58-1.11) | 2.67E-01 | #N/A |  | 0.98 (0.65-1.46) | 9.59E-01 | #N/A |
| PC 36:4b |  | 1.13 (0.79-1.63) | 6.16E-01 | 4.4 |  | 1.01 (0.69-1.47) | 9.94E-01 | 0.8 |
| PC 36:5 |  | 1.39 (1.04-1.86) | 5.23E-02 | 12.2 |  | 1.35 (1.02-1.80) | 7.49E-02 | 13.6 |
| PC 37:4 |  | 0.52 (0.35-0.78) | **4.08E-03** | -9.8 |  | 0.51 (0.32-0.81) | **1.37E-02** | -7.3 |
| PC 37:5 |  | 0.97 (0.72-1.31) | 8.73E-01 | -3.1 |  | 0.98 (0.64-1.51) | 9.80E-01 | 0.3 |
| PC 37:6 |  | 1.04 (0.79-1.38) | 8.53E-01 | -2.1 |  | 1.12 (0.78-1.60) | 6.48E-01 | 2.5 |
| PC 38:2 |  | 0.95 (0.67-1.35) | 8.55E-01 | 0.5 |  | 1.20 (0.81-1.77) | 4.97E-01 | 3.5 |
| PC 38:3 |  | 0.82 (0.58-1.16) | 3.60E-01 | -0.5 |  | 0.92 (0.63-1.33) | 7.46E-01 | 0.5 |
| PC 38:4 |  | 1.19 (0.83-1.70) | 4.40E-01 | 4.9 |  | 1.11 (0.75-1.65) | 7.00E-01 | 3.4 |
| PC 38:5 |  | 1.09 (0.79-1.51) | 6.87E-01 | 1.9 |  | 1.20 (0.86-1.69) | 3.88E-01 | 4.1 |
| PC 38:6a |  | 0.88 (0.63-1.22) | 5.38E-01 | -3.9 |  | 1.07 (0.75-1.52) | 8.03E-01 | -0.2 |
| PC 38:6b |  | 1.46 (1.05-2.04) | 5.15E-02 | 5.7 |  | 1.44 (0.96-2.18) | 1.38E-01 | 5.6 |
| PC 40:5 |  | 1.46 (1.04-2.04) | 5.65E-02 | 10.9 |  | 1.77 (1.15-2.74) | **2.47E-02** | 12.8 |
| PC 40:6 |  | 1.68 (1.19-2.36) | **8.47E-03** | 13.2 |  | 1.65 (1.13-2.41) | **2.42E-02** | 13.5 |
| PC 40:7 |  | 1.07 (0.75-1.54) | 7.75E-01 | -3.9 |  | 1.18 (0.77-1.82) | 5.73E-01 | 2.2 |
| PC(O-30:0) |  | 0.72 (0.51-1.02) | 1.11E-01 | -8.2 |  | 0.72 (0.47-1.12) | 2.25E-01 | -5.2 |
| PC(O-32:0) |  | 0.84 (0.61-1.14) | 3.62E-01 | -3.0 |  | 0.59 (0.38-0.90) | **3.25E-02** | -5.2 |
| PC(O-32:1) |  | 0.97 (0.69-1.38) | 9.00E-01 | -3.5 |  | 0.99 (0.63-1.56) | 9.89E-01 | -1.6 |
| PC(O-34:0) |  | 0.73 (0.52-1.02) | 1.16E-01 | -4.7 |  | 0.60 (0.38-0.93) | **4.74E-02** | -4.7 |
| PC(O-34:1) |  | 0.71 (0.48-1.04) | 1.34E-01 | -5.7 |  | 0.53 (0.33-0.84) | **2.00E-02** | -5.4 |
| PC(O-34:2) |  | 0.49 (0.33-0.75) | **3.21E-03** | -13.6 |  | 0.60 (0.38-0.96) | 6.74E-02 | -8.9 |
| PC(O-36:0) |  | 0.90 (0.65-1.26) | 6.43E-01 | -1.3 |  | 0.73 (0.47-1.13) | 2.39E-01 | -2.6 |
| PC(O-36:1) |  | 0.71 (0.50-1.02) | 1.11E-01 | -8.2 |  | 0.53 (0.33-0.85) | **2.39E-02** | -8.0 |
| PC(O-36:2) |  | 0.51 (0.35-0.76) | **3.05E-03** | -12.6 |  | 0.49 (0.30-0.79) | **1.15E-02** | -9.0 |
| PC(O-36:3) |  | 0.63 (0.44-0.89) | **2.07E-02** | -6.8 |  | 0.55 (0.35-0.85) | **2.19E-02** | -7.4 |
| PC(O-36:4) |  | 0.70 (0.50-0.97) | 6.57E-02 | -4.4 |  | 0.63 (0.42-0.92) | **4.02E-02** | -5.6 |
| PC(O-36:5) |  | 1.17 (0.89-1.55) | 3.62E-01 | 2.7 |  | 1.20 (0.88-1.65) | 3.48E-01 | 4.9 |
| PC(O-38:4) |  | 0.71 (0.50-0.99) | 8.58E-02 | -4.4 |  | 0.59 (0.39-0.90) | **3.57E-02** | -5.5 |
| PC(O-38:5) |  | 0.68 (0.47-0.97) | 6.78E-02 | -5.4 |  | 0.58 (0.37-0.90) | **3.63E-02** | -5.0 |
| PC(O-40:7) |  | 1.02 (0.72-1.44) | 9.32E-01 | -3.9 |  | 0.99 (0.64-1.53) | 9.94E-01 | -0.4 |
| PC(P-32:0) |  | 0.71 (0.50-1.00) | 9.26E-02 | -6.5 |  | 0.63 (0.43-0.94) | 5.27E-02 | -4.6 |
| PC(P-34:1) |  | 0.71 (0.48-1.06) | 1.55E-01 | -9.5 |  | 0.57 (0.36-0.91) | **3.92E-02** | -6.9 |
| PC(P-36:2) |  | 0.52 (0.36-0.76) | **2.74E-03** | -16.6 |  | 0.53 (0.34-0.84) | **2.01E-02** | -10.4 |
| PC(P-36:5) |  | 1.00 (0.75-1.33) | 9.90E-01 | -0.5 |  | 1.13 (0.78-1.64) | 6.28E-01 | 3.0 |
| PC(P-38:5) |  | 0.89 (0.63-1.27) | 6.31E-01 | -4.1 |  | 0.90 (0.56-1.43) | 7.46E-01 | -1.6 |
| LPC 14:0 |  | 1.09 (0.78-1.52) | 6.99E-01 | 4.1 |  | 1.69 (1.19-2.41) | **1.21E-02** | 15.5 |
| LPC 15:0 |  | 0.67 (0.48-0.95) | 5.12E-02 | -9.1 |  | 1.09 (0.74-1.61) | 7.46E-01 | 1.9 |
| LPC 16:0 |  | 1.19 (0.89-1.59) | 3.32E-01 | 1.8 |  | 1.64 (1.13-2.39) | **2.47E-02** | 7.4 |
| LPC 16:1 |  | 1.29 (0.93-1.78) | 1.96E-01 | 5.2 |  | 1.76 (1.20-2.57) | **1.21E-02** | 11.8 |
| LPC 18:0 |  | 1.05 (0.77-1.44) | 8.21E-01 | -1.5 |  | 1.68 (1.15-2.45) | **2.16E-02** | 8.8 |
| LPC 18:1 |  | 0.75 (0.51-1.10) | 2.18E-01 | -8.6 |  | 1.23 (0.81-1.86) | 4.45E-01 | 2.0 |
| LPC 18:2 |  | 0.54 (0.35-0.83) | **1.31E-02** | -14.0 |  | 0.76 (0.47-1.22) | 3.53E-01 | -5.8 |
| LPC 20:0 |  | 0.84 (0.59-1.19) | 4.11E-01 | -9.9 |  | 1.13 (0.76-1.67) | 6.58E-01 | 0.4 |
| LPC 20:1 |  | 0.72 (0.51-1.02) | 1.16E-01 | -12.6 |  | 1.16 (0.77-1.73) | 5.93E-01 | 0.8 |
| LPC 20:2 |  | 0.82 (0.57-1.17) | 3.68E-01 | -8.3 |  | 1.37 (0.88-2.13) | 2.52E-01 | 4.0 |
| LPC 20:3 |  | 1.04 (0.75-1.43) | 8.72E-01 | 2.2 |  | 1.30 (0.89-1.91) | 2.59E-01 | 5.1 |
| LPC 20:4 |  | 0.90 (0.64-1.27) | 6.42E-01 | -4.3 |  | 1.10 (0.75-1.61) | 7.19E-01 | 1.1 |
| LPC 20:5 |  | 1.19 (0.93-1.51) | 2.55E-01 | 4.1 |  | 1.31 (1.02-1.68) | 6.93E-02 | 14.9 |
| LPC 22:0 |  | 0.75 (0.53-1.04) | 1.40E-01 | -12.5 |  | 0.86 (0.62-1.21) | 5.12E-01 | -3.6 |
| LPC 22:6 |  | 1.40 (1.04-1.89) | 5.65E-02 | 2.2 |  | 1.67 (1.14-2.46) | **2.38E-02** | 10.4 |
| LPC 24:0 |  | 0.92 (0.68-1.24) | 6.88E-01 | -6.3 |  | 1.00 (0.74-1.35) | 9.95E-01 | -0.2 |
| LPC(O-22:0) |  | 0.60 (0.42-0.86) | **1.33E-02** | -11.6 |  | 0.98 (0.65-1.47) | 9.59E-01 | -0.5 |
| LPC(O-24:1) |  | 0.55 (0.38-0.80) | **5.34E-03** | -14.1 |  | 0.82 (0.56-1.21) | 4.45E-01 | -3.0 |
| LPC(O-24:2) |  | 0.52 (0.34-0.78) | **5.75E-03** | -21.5 |  | 0.69 (0.46-1.03) | 1.20E-01 | -11.0 |
| PE 32:1 |  | 1.66 (1.21-2.27) | **5.45E-03** | 53.9 |  | 1.50 (1.07-2.12) | **4.41E-02** | 28.0 |
| PE 34:1 |  | 1.88 (1.34-2.64) | **1.31E-03** | 39.8 |  | 1.50 (1.04-2.15) | 5.82E-02 | 19.8 |
| PE 34:2 |  | 1.64 (1.14-2.34) | **1.61E-02** | 28.6 |  | 1.59 (1.03-2.45) | 7.13E-02 | 18.7 |
| PE 36:1 |  | 2.14 (1.46-3.13) | **6.47E-04** | 41.0 |  | 1.95 (1.28-2.96) | **7.21E-03** | 24.2 |
| PE 36:2 |  | 2.12 (1.44-3.11) | **8.28E-04** | 35.2 |  | 1.93 (1.25-2.96) | **1.01E-02** | 23.5 |
| PE 36:3 |  | 1.59 (1.12-2.24) | **2.01E-02** | 22.4 |  | 1.54 (0.98-2.41) | 1.09E-01 | 13.7 |
| PE 36:4 |  | 1.64 (1.16-2.31) | **1.26E-02** | 24.7 |  | 1.41 (0.93-2.14) | 1.69E-01 | 11.8 |
| PE 36:5 |  | 1.70 (1.17-2.48) | **1.28E-02** | 25.5 |  | 1.53 (1.08-2.17) | **3.78E-02** | 22.5 |
| PE 38:3 |  | 2.05 (1.38-3.04) | **1.81E-03** | 34.2 |  | 1.82 (1.16-2.86) | **2.47E-02** | 19.3 |
| PE 38:4 |  | 1.99 (1.32-3.00) | **3.38E-03** | 28.7 |  | 1.70 (1.06-2.71) | 5.63E-02 | 16.9 |
| PE 38:5 |  | 1.64 (1.15-2.34) | **1.50E-02** | 21.6 |  | 1.55 (1.04-2.29) | 6.12E-02 | 16.0 |
| PE 38:6 |  | 2.21 (1.47-3.32) | **8.28E-04** | 31.8 |  | 1.70 (1.15-2.50) | **2.19E-02** | 20.2 |
| PE 40:6 |  | 2.66 (1.75-4.04) | **1.10E-04** | 48.5 |  | 2.19 (1.44-3.33) | **1.51E-03** | 33.4 |
| PE 40:7 |  | 1.82 (1.25-2.64) | **5.34E-03** | 24.7 |  | 1.49 (1.05-2.11) | 5.33E-02 | 19.0 |
| PI 32:0 |  | 1.59 (1.17-2.16) | **8.78E-03** | 37.9 |  | 1.53 (1.12-2.09) | **2.16E-02** | 19.1 |
| PI 32:1 |  | 2.26 (1.53-3.33) | **3.86E-04** | 67.0 |  | 1.86 (1.29-2.67) | **3.68E-03** | 39.8 |
| PI 34:0 |  | 1.75 (1.25-2.44) | **3.51E-03** | 28.5 |  | 1.76 (1.19-2.61) | **1.41E-02** | 15.9 |
| PI 34:1 |  | 1.73 (1.25-2.40) | **3.40E-03** | 27.5 |  | 1.81 (1.21-2.69) | **1.21E-02** | 15.7 |
| PI 36:0 |  | 0.97 (0.69-1.34) | 8.73E-01 | 1.5 |  | 1.33 (0.91-1.94) | 2.19E-01 | 7.0 |
| PI 36:1 |  | 1.12 (0.81-1.55) | 5.83E-01 | 5.6 |  | 1.37 (0.92-2.05) | 1.91E-01 | 6.5 |
| PI 36:2 |  | 1.48 (1.00-2.18) | 9.28E-02 | 8.9 |  | 1.72 (1.13-2.62) | **2.94E-02** | 10.7 |
| PI 36:3 |  | 1.31 (0.89-1.91) | 2.55E-01 | 7.9 |  | 1.58 (0.98-2.56) | 1.10E-01 | 9.2 |
| PI 36:4 |  | 1.77 (1.21-2.58) | **9.07E-03** | 20.1 |  | 1.60 (1.06-2.42) | 5.33E-02 | 14.6 |
| PI 38:2 |  | 1.33 (0.96-1.85) | 1.50E-01 | 9.8 |  | 1.49 (0.99-2.25) | 1.03E-01 | 10.7 |
| PI 38:3 |  | 1.29 (0.87-1.90) | 2.98E-01 | 7.8 |  | 1.69 (1.01-2.83) | 8.32E-02 | 10.7 |
| PI 38:4 |  | 1.24 (0.86-1.79) | 3.47E-01 | 5.9 |  | 1.51 (0.95-2.39) | 1.36E-01 | 8.9 |
| PI 38:5 |  | 1.35 (0.92-1.97) | 1.96E-01 | 7.6 |  | 1.74 (1.10-2.76) | **3.98E-02** | 12.0 |
| PI 38:6 |  | 2.18 (1.48-3.20) | **5.63E-04** | 19.9 |  | 2.08 (1.39-3.10) | **2.12E-03** | 19.2 |
| PI 40:4 |  | 1.71 (1.17-2.50) | **1.31E-02** | 20.0 |  | 1.96 (1.18-3.23) | **2.39E-02** | 14.9 |
| PI 40:5 |  | 1.92 (1.33-2.77) | **2.24E-03** | 21.6 |  | 2.38 (1.52-3.72) | **1.10E-03** | 20.5 |
| PI 40:6 |  | 2.43 (1.64-3.61) | **1.64E-04** | 23.5 |  | 1.83 (1.26-2.67) | **6.19E-03** | 17.0 |
| PS 36:1 |  | 1.01 (0.81-1.25) | 9.58E-01 | 7.4 |  | 0.91 (0.70-1.20) | 6.32E-01 | -4.5 |
| PS 36:2 |  | 0.94 (0.71-1.24) | 7.19E-01 | 2.3 |  | 1.09 (0.81-1.48) | 6.68E-01 | 5.3 |
| PS 38:3 |  | 1.22 (0.88-1.69) | 3.31E-01 | 11.1 |  | 1.14 (0.84-1.56) | 5.12E-01 | 5.7 |
| PS 38:4 |  | 1.15 (0.88-1.49) | 3.96E-01 | 11.7 |  | 1.17 (0.85-1.60) | 4.60E-01 | 7.3 |
| PS 38:5 |  | 1.19 (0.87-1.61) | 3.70E-01 | 6.9 |  | 1.04 (0.73-1.48) | 8.89E-01 | 1.5 |
| PS 40:5 |  | 1.33 (0.99-1.79) | 1.01E-01 | 18.9 |  | 1.06 (0.78-1.43) | 8.05E-01 | 5.3 |
| PS 40:6 |  | 1.71 (1.19-2.45) | **1.01E-02** | 22.7 |  | 1.18 (0.90-1.55) | 3.21E-01 | 12.1 |
| PG 34:1 |  | 1.43 (1.03-2.00) | 6.27E-02 | 17.3 |  | 1.40 (0.96-2.03) | 1.38E-01 | 13.5 |
| PG 34:2 |  | 1.40 (0.92-2.13) | 1.84E-01 | 18.7 |  | 2.26 (1.43-3.58) | **2.53E-03** | 18.9 |
| PG 36:1 |  | 1.87 (1.30-2.70) | **3.05E-03** | 44.0 |  | 1.98 (1.34-2.93) | **3.04E-03** | 36.8 |
| PG 36:2 |  | 2.10 (1.43-3.08) | **9.20E-04** | 36.9 |  | 1.96 (1.32-2.90) | **3.60E-03** | 30.2 |
| COH |  | 1.07 (0.80-1.44) | 7.16E-01 | 2.8 |  | 1.72 (1.17-2.53) | **1.70E-02** | 21.1 |
| CE 14:0 |  | 2.03 (1.44-2.87) | **4.73E-04** | 51.4 |  | 2.15 (1.41-3.29) | **2.18E-03** | 34.4 |
| CE 15:0 |  | 1.42 (1.02-1.97) | 7.44E-02 | 19.3 |  | 1.40 (0.92-2.12) | 1.89E-01 | 12.6 |
| CE 16:0 |  | 1.59 (1.15-2.21) | **1.26E-02** | 15.6 |  | 1.44 (0.95-2.19) | 1.42E-01 | 8.3 |
| CE 16:1 |  | 2.88 (1.89-4.39) | **5.94E-05** | 73.0 |  | 2.08 (1.40-3.08) | **1.88E-03** | 39.8 |
| CE 16:2 |  | 2.87 (1.89-4.36) | **5.88E-05** | 59.3 |  | 2.30 (1.50-3.53) | **1.10E-03** | 35.9 |
| CE 17:0 |  | 1.04 (0.77-1.40) | 8.59E-01 | 4.4 |  | 0.93 (0.60-1.43) | 8.09E-01 | 0.7 |
| CE 17:1 |  | 1.61 (1.16-2.25) | **1.16E-02** | 24.7 |  | 1.19 (0.81-1.76) | 4.97E-01 | 8.0 |
| CE 18:0 |  | 1.18 (0.87-1.61) | 3.86E-01 | 7.1 |  | 1.38 (0.92-2.07) | 1.87E-01 | 9.1 |
| CE 18:1 |  | 1.88 (1.31-2.71) | **2.41E-03** | 19.1 |  | 1.28 (0.86-1.90) | 3.23E-01 | 7.9 |
| CE 18:2 |  | 2.19 (1.53-3.13) | **2.19E-04** | 29.8 |  | 1.67 (1.14-2.44) | **2.19E-02** | 16.3 |
| CE 18:3 |  | 2.24 (1.56-3.23) | **1.84E-04** | 45.1 |  | 1.74 (1.25-2.44) | **5.14E-03** | 29.5 |
| CE 20:1 |  | 0.91 (0.69-1.21) | 6.31E-01 | -6.9 |  | 0.79 (0.54-1.13) | 2.90E-01 | -7.8 |
| CE 20:2 |  | 1.31 (0.97-1.77) | 1.31E-01 | 19.1 |  | 1.16 (0.92-1.47) | 3.12E-01 | 9.9 |
| CE 20:3 |  | 2.80 (1.82-4.29) | **8.00E-05** | 38.9 |  | 1.86 (1.22-2.84) | **1.37E-02** | 19.2 |
| CE 20:4 |  | 2.15 (1.54-3.01) | **1.39E-04** | 35.4 |  | 1.55 (1.03-2.35) | 7.28E-02 | 14.8 |
| CE 20:5 |  | 2.17 (1.54-3.05) | **1.60E-04** | 51.6 |  | 1.46 (1.08-1.97) | **3.45E-02** | 28.6 |
| CE 22:0 |  | 1.73 (1.30-2.30) | **1.03E-03** | 48.1 |  | 2.19 (1.50-3.20) | **5.19E-04** | 41.7 |
| CE 22:1 |  | 1.29 (1.00-1.68) | 9.28E-02 | 17.4 |  | 1.23 (0.93-1.64) | 2.30E-01 | 14.7 |
| CE 22:4 |  | 1.91 (1.32-2.76) | **2.36E-03** | 25.1 |  | 1.42 (0.93-2.17) | 1.69E-01 | 10.4 |
| CE 22:5 |  | 2.29 (1.60-3.27) | **1.21E-04** | 36.3 |  | 1.72 (1.18-2.50) | **1.47E-02** | 19.5 |
| CE 22:6 |  | 2.18 (1.54-3.10) | **1.82E-04** | 43.1 |  | 1.42 (1.08-1.86) | **2.71E-02** | 23.4 |
| CE 24:0 |  | 1.58 (1.24-2.00) | **1.07E-03** | 60.9 |  | 1.71 (1.21-2.41) | **8.78E-03** | 34.0 |
| CE 24:1 |  | 1.79 (1.31-2.46) | **1.31E-03** | 30.5 |  | 1.76 (1.22-2.53) | **9.68E-03** | 22.8 |
| CE 24:4 |  | 1.58 (1.12-2.23) | **2.04E-02** | 14.2 |  | 1.26 (0.81-1.97) | 4.11E-01 | 7.0 |
| CE 24:5 |  | 2.20 (1.54-3.14) | **1.98E-04** | 38.8 |  | 1.73 (1.15-2.58) | **2.19E-02** | 16.7 |
| CE 24:6 |  | 1.91 (1.38-2.66) | **7.50E-04** | 51.5 |  | 1.62 (1.11-2.36) | **2.84E-02** | 21.5 |
| DG 14:0/16:0 |  | 2.06 (1.48-2.87) | **2.30E-04** | 94.6 |  | 2.02 (1.44-2.83) | **5.06E-04** | 84.1 |
| DG 14:0/18:1 |  | 2.11 (1.46-3.04) | **5.89E-04** | 63.9 |  | 2.60 (1.68-4.03) | **3.75E-04** | 58.7 |
| DG 14:0/18:2 |  | 1.76 (1.28-2.42) | **1.91E-03** | 58.7 |  | 2.20 (1.52-3.19) | **4.42E-04** | 59.6 |
| DG 16:0/16:0 |  | 2.71 (1.86-3.96) | **5.23E-05** | 110.0 |  | 2.17 (1.52-3.11) | **3.75E-04** | 86.4 |
| DG 16:0/18:0 |  | 2.60 (1.76-3.83) | **6.32E-05** | 80.7 |  | 2.55 (1.68-3.85) | **3.64E-04** | 65.0 |
| DG 16:0/18:1 |  | 2.95 (1.94-4.48) | **5.23E-05** | 70.4 |  | 2.68 (1.76-4.09) | **3.64E-04** | 56.3 |
| DG 16:0/18:2 |  | 2.57 (1.72-3.82) | **9.86E-05** | 64.0 |  | 2.40 (1.63-3.55) | **3.64E-04** | 50.1 |
| DG 16:0/20:3 |  | 2.16 (1.52-3.06) | **1.98E-04** | 63.3 |  | 2.03 (1.38-2.99) | **1.98E-03** | 42.3 |
| DG 16:0/20:4 |  | 2.24 (1.55-3.23) | **1.98E-04** | 70.2 |  | 2.03 (1.40-2.94) | **1.17E-03** | 53.5 |
| DG 16:0/22:5 |  | 2.46 (1.70-3.56) | **6.32E-05** | 62.4 |  | 2.19 (1.52-3.14) | **3.83E-04** | 57.3 |
| DG 16:0/22:6 |  | 3.05 (2.03-4.59) | **5.23E-05** | 90.7 |  | 2.29 (1.58-3.32) | **3.64E-04** | 107.1 |
| DG 16:1/18:0 |  | 2.62 (1.77-3.88) | **6.32E-05** | 86.6 |  | 2.80 (1.77-4.42) | **3.64E-04** | 67.7 |
| DG 16:1/18:1 |  | 2.79 (1.82-4.27) | **8.00E-05** | 64.9 |  | 2.98 (1.85-4.81) | **3.64E-04** | 54.1 |
| DG 18:0/18:1 |  | 2.95 (1.91-4.56) | **6.32E-05** | 72.7 |  | 2.75 (1.79-4.22) | **3.64E-04** | 56.2 |
| DG 18:0/18:2 |  | 2.20 (1.56-3.11) | **1.39E-04** | 61.3 |  | 2.28 (1.58-3.28) | **3.64E-04** | 50.5 |
| DG 18:0/20:4 |  | 1.96 (1.35-2.86) | **1.87E-03** | 25.7 |  | 2.00 (1.35-2.97) | **2.68E-03** | 23.1 |
| DG 18:1/18:1 |  | 2.32 (1.62-3.34) | **1.19E-04** | 52.6 |  | 2.73 (1.75-4.25) | **3.64E-04** | 43.1 |
| DG 18:1/18:2 |  | 2.18 (1.51-3.14) | **3.18E-04** | 47.2 |  | 2.37 (1.53-3.69) | **9.63E-04** | 38.9 |
| DG 18:1/18:3 |  | 1.83 (1.32-2.53) | **1.49E-03** | 42.5 |  | 2.00 (1.40-2.86) | **1.10E-03** | 41.6 |
| DG 18:1/20:3 |  | 1.65 (1.17-2.32) | **1.10E-02** | 31.1 |  | 1.76 (1.17-2.64) | **1.95E-02** | 25.5 |
| DG 18:1/20:4 |  | 2.01 (1.43-2.83) | **5.39E-04** | 46.5 |  | 1.83 (1.30-2.57) | **2.53E-03** | 38.9 |
| DG 18:2/18:2 |  | 1.73 (1.26-2.38) | **2.49E-03** | 52.5 |  | 1.88 (1.27-2.76) | **5.86E-03** | 40.2 |
| TG 14:0/16:0/18:1 |  | 1.81 (1.31-2.50) | **1.40E-03** | 64.3 |  | 2.13 (1.48-3.07) | **5.15E-04** | 62.1 |
| TG 14:0/16:0/18:2 |  | 1.84 (1.34-2.52) | **8.61E-04** | 73.1 |  | 2.25 (1.55-3.27) | **3.75E-04** | 69.3 |
| TG 14:0/16:1/18:1 |  | 1.56 (1.13-2.14) | **1.61E-02** | 45.8 |  | 2.26 (1.51-3.38) | **7.09E-04** | 53.1 |
| TG 14:0/16:1/18:2 |  | 1.42 (1.09-1.85) | **2.27E-02** | 43.1 |  | 1.96 (1.39-2.76) | **9.81E-04** | 57.7 |
| TG 14:0/17:0/18:1 |  | 1.61 (1.14-2.26) | **1.53E-02** | 47.7 |  | 1.82 (1.30-2.54) | **2.48E-03** | 48.3 |
| TG 14:0/18:0/18:1 |  | 1.94 (1.37-2.75) | **1.08E-03** | 72.0 |  | 2.22 (1.50-3.30) | **7.09E-04** | 65.8 |
| TG 14:0/18:2/18:2 |  | 1.48 (1.12-1.97) | **1.52E-02** | 40.4 |  | 1.82 (1.29-2.55) | **2.68E-03** | 52.3 |
| TG 14:1/16:0/18:1 |  | 1.65 (1.17-2.31) | **1.10E-02** | 56.9 |  | 2.22 (1.50-3.30) | **7.09E-04** | 59.8 |
| TG 14:1/16:1/18:0 |  | 1.96 (1.42-2.72) | **4.45E-04** | 86.6 |  | 2.28 (1.58-3.30) | **3.64E-04** | 74.5 |
| TG 14:1/18:0/18:2 |  | 1.45 (1.09-1.92) | **2.32E-02** | 33.9 |  | 2.36 (1.58-3.51) | **4.04E-04** | 43.3 |
| TG 14:1/18:1/18:1 |  | 1.62 (1.19-2.21) | **6.55E-03** | 37.7 |  | 2.65 (1.70-4.14) | **3.75E-04** | 45.9 |
| TG 15:0/16:0/18:1 |  | 1.84 (1.29-2.64) | **3.19E-03** | 58.7 |  | 1.91 (1.36-2.68) | **1.32E-03** | 54.4 |
| TG 15:0/18:1/18:1 |  | 1.37 (1.02-1.85) | 7.29E-02 | 25.8 |  | 1.78 (1.23-2.59) | **8.95E-03** | 29.5 |
| TG 16:0/16:0/16:0 |  | 2.13 (1.55-2.92) | **8.72E-05** | 125.0 |  | 1.93 (1.41-2.65) | **5.14E-04** | 89.3 |
| TG 16:0/16:0/18:0 |  | 1.95 (1.42-2.68) | **3.80E-04** | 108.8 |  | 1.88 (1.36-2.59) | **9.63E-04** | 75.1 |
| TG 16:0/16:0/18:1 |  | 2.55 (1.78-3.67) | **5.23E-05** | 84.7 |  | 2.33 (1.59-3.39) | **3.64E-04** | 64.8 |
| TG 16:0/16:0/18:2 |  | 2.53 (1.74-3.70) | **6.32E-05** | 77.9 |  | 2.07 (1.49-2.89) | **3.75E-04** | 64.5 |
| TG 16:0/16:1/17:0 |  | 1.92 (1.34-2.74) | **1.50E-03** | 67.1 |  | 1.95 (1.38-2.75) | **1.05E-03** | 58.2 |
| TG 16:0/16:1/18:1 |  | 2.11 (1.47-3.03) | **4.45E-04** | 54.1 |  | 2.85 (1.82-4.45) | **3.64E-04** | 50.3 |
| TG 16:0/17:0/18:0 |  | 2.05 (1.45-2.92) | **4.92E-04** | 68.7 |  | 1.93 (1.36-2.74) | **1.49E-03** | 48.5 |
| TG 16:0/17:0/18:1 |  | 1.95 (1.36-2.79) | **1.32E-03** | 61.3 |  | 1.86 (1.32-2.61) | **2.03E-03** | 46.0 |
| TG 16:0/17:0/18:2 |  | 1.99 (1.36-2.90) | **1.58E-03** | 51.3 |  | 2.22 (1.51-3.28) | **5.78E-04** | 43.9 |
| TG 16:0/18:0/18:1 |  | 2.42 (1.65-3.55) | **1.21E-04** | 87.0 |  | 2.39 (1.58-3.63) | **4.96E-04** | 60.1 |
| TG 16:0/18:1/18:1 |  | 2.39 (1.63-3.52) | **1.45E-04** | 43.3 |  | 2.74 (1.76-4.27) | **3.64E-04** | 38.2 |
| TG 16:0/18:1/18:2 |  | 2.09 (1.47-2.97) | **3.87E-04** | 40.8 |  | 2.35 (1.57-3.52) | **4.42E-04** | 39.2 |
| TG 16:0/18:2/18:2 |  | 1.96 (1.40-2.74) | **6.48E-04** | 47.2 |  | 2.07 (1.43-2.99) | **9.28E-04** | 46.2 |
| TG 16:1/16:1/16:1 |  | 1.64 (1.21-2.22) | **5.01E-03** | 56.0 |  | 2.18 (1.54-3.10) | **3.64E-04** | 65.5 |
| TG 16:1/16:1/18:0 |  | 1.95 (1.39-2.72) | **6.85E-04** | 68.9 |  | 2.47 (1.63-3.72) | **3.75E-04** | 64.5 |
| TG 16:1/16:1/18:1 |  | 2.23 (1.52-3.27) | **3.89E-04** | 57.0 |  | 2.73 (1.78-4.20) | **3.64E-04** | 56.6 |
| TG 16:1/17:0/18:1 |  | 1.60 (1.14-2.25) | **1.61E-02** | 34.7 |  | 2.09 (1.40-3.13) | **2.01E-03** | 34.0 |
| TG 16:1/18:1/18:1 |  | 1.76 (1.26-2.45) | **3.27E-03** | 34.7 |  | 2.65 (1.70-4.12) | **3.75E-04** | 39.0 |
| TG 16:1/18:1/18:2 |  | 1.79 (1.29-2.48) | **2.16E-03** | 37.1 |  | 2.32 (1.56-3.44) | **4.37E-04** | 44.6 |
| TG 17:0/18:1/18:1 |  | 1.57 (1.16-2.11) | **8.71E-03** | 33.8 |  | 1.81 (1.25-2.62) | **6.21E-03** | 28.6 |
| TG 18:0/18:0/18:1 |  | 1.78 (1.33-2.39) | **6.85E-04** | 102.3 |  | 1.78 (1.30-2.43) | **1.98E-03** | 66.9 |
| TG 18:0/18:1/18:1 |  | 2.25 (1.58-3.22) | **1.39E-04** | 61.2 |  | 2.30 (1.56-3.40) | **4.37E-04** | 45.7 |
| TG 18:0/18:2/18:2 |  | 1.91 (1.38-2.65) | **6.47E-04** | 41.5 |  | 2.23 (1.51-3.30) | **6.51E-04** | 40.3 |
| TG 18:1/18:1/18:1 |  | 1.88 (1.34-2.63) | **1.27E-03** | 33.4 |  | 2.29 (1.50-3.48) | **9.62E-04** | 34.2 |
| TG 18:1/18:1/18:2 |  | 1.65 (1.21-2.26) | **5.22E-03** | 26.6 |  | 1.85 (1.27-2.70) | **5.44E-03** | 30.8 |
| TG 18:1/18:1/20:4 |  | 1.72 (1.24-2.39) | **3.97E-03** | 24.0 |  | 2.04 (1.37-3.03) | **2.28E-03** | 28.7 |
| TG 18:1/18:1/22:6 |  | 2.37 (1.61-3.49) | **1.80E-04** | 35.1 |  | 2.39 (1.61-3.53) | **3.64E-04** | 49.7 |
| TG 18:1/18:2/18:2 |  | 1.62 (1.21-2.17) | **3.72E-03** | 32.0 |  | 1.84 (1.27-2.67) | **5.65E-03** | 37.3 |
| TG 18:2/18:2/18:2 |  | 1.40 (1.11-1.77) | **1.15E-02** | 40.2 |  | 1.53 (1.12-2.09) | **2.19E-02** | 39.5 |
| TG 18:2/18:2/20:4 |  | 1.49 (1.10-2.02) | **2.27E-02** | 23.5 |  | 1.64 (1.18-2.28) | **1.17E-02** | 34.5 |

^a^ Logistic regression of T2D (n = 116) against NGT (n = 168) on lipid species adjusted for age, sex, waist circumference and SBP.

^b^ Logistic regression of prediabetes (n = 64) against NGT (n = 168) on lipid species adjusted for age, sex, waist circumference and SBP.

^c^ Odds ratio (95% confidence intervals) based on an interquartile range increase in predictor lipid species measurement.

^d^ Benjamini-Hochberg corrected p-value. Bold type indicates corrected p < 0.05.

^e^ % difference in means between groups.

**Table S4. Logistic regression of lipid classes in the SAFHS cohort.**

|  |  | **Diabetes vs. NGT^a^** | | |  | **Prediabetes vs. NGT^b^** | | |
| --- | --- | --- | --- | --- | --- | --- | --- | --- |
| **Lipid Class** |  | **Odds Ratio^c^** | **p-value^d^** | **% difference^e^** |  | **Odds Ratio^c^** | **p-value^d^** | **% difference^e^** |
| Dihydroceramide |  | 1.85 (1.42-2.40) | **1.81E-05** | 31.1 |  | 1.32 (1.04-1.67) | 5.24E-02 | 18.7 |
| Ceramide |  | 1.72 (1.30-2.28) | **4.06E-04** | 18.2 |  | 1.38 (1.07-1.77) | **3.08E-02** | 11.3 |
| Monohexosylceramide |  | 1.33 (1.00-1.76) | 7.45E-02 | 12.0 |  | 1.00 (0.76-1.31) | 9.95E-01 | 2.1 |
| Dihexosylceramide |  | 0.85 (0.62-1.16) | 3.29E-01 | -1.1 |  | 0.81 (0.61-1.06) | 2.13E-01 | -3.1 |
| Trihexosylceramide |  | 0.72 (0.54-0.96) | **4.46E-02** | 2.3 |  | 0.77 (0.60-0.99) | 7.92E-02 | -0.4 |
| GM3 ganglioside |  | 1.18 (0.88-1.58) | 3.27E-01 | 8.0 |  | 0.86 (0.66-1.13) | 3.67E-01 | -0.7 |
| Sphingomyelin |  | 0.83 (0.60-1.16) | 3.27E-01 | 3.1 |  | 0.89 (0.67-1.18) | 4.85E-01 | 3.2 |
| Phosphatidylcholine |  | 1.76 (1.27-2.45) | **1.79E-03** | 7.7 |  | 1.69 (1.27-2.25) | **9.70E-04** | 6.7 |
| Alkylphosphatidylcholine |  | 0.75 (0.53-1.08) | 1.76E-01 | -6.0 |  | 0.83 (0.63-1.11) | 3.08E-01 | -4.1 |
| Alkenylphosphatidylcholine |  | 0.59 (0.42-0.83) | **5.23E-03** | -9.2 |  | 0.64 (0.49-0.85) | **6.25E-03** | -7.3 |
| Lysophosphatidylcholine |  | 1.03 (0.78-1.38) | 8.18E-01 | 0.2 |  | 0.84 (0.64-1.11) | 3.08E-01 | -5.5 |
| Lysoalkylphosphatidylcholine |  | 0.68 (0.48-0.97) | 6.16E-02 | -8.1 |  | 0.73 (0.54-0.97) | 6.71E-02 | -9.2 |
| Phosphatidylethanolamine |  | 2.88 (2.07-4.01) | **4.15E-09** | 47.5 |  | 1.72 (1.36-2.18) | **4.98E-05** | 27.3 |
| Phosphatidylinositol |  | 1.90 (1.42-2.55) | **5.76E-05** | 25.1 |  | 1.66 (1.28-2.16) | **5.51E-04** | 16.7 |
| Phosphatidylserine |  | 0.93 (0.70-1.23) | 6.24E-01 | -9.4 |  | 1.20 (0.97-1.48) | 1.53E-01 | 8.3 |
| Phosphatidylglycerol |  | 2.85 (2.10-3.88) | **5.14E-10** | 64.4 |  | 1.71 (1.37-2.13) | **4.98E-05** | 34.2 |
| Free cholesterol |  | 1.18 (0.89-1.56) | 3.18E-01 | 15.3 |  | 1.16 (0.90-1.48) | 3.47E-01 | 10.3 |
| Cholesterol ester |  | 2.59 (1.96-3.42) | **5.14E-10** | 33.3 |  | 1.67 (1.32-2.10) | **1.04E-04** | 18.2 |
| Diacylglycerol |  | 1.84 (1.48-2.28) | **1.86E-07** | 61.7 |  | 1.47 (1.22-1.77) | **1.92E-04** | 38.5 |
| Triacylglycerol |  | 2.17 (1.69-2.78) | **1.04E-08** | 56.9 |  | 1.64 (1.33-2.04) | **4.98E-05** | 37.8 |

^a^ Logistic regression of T2D (n = 69) against NGT (n = 796) on lipid classes adjusted for age, sex, waist circumference and SBP.

^b^ Logistic regression of prediabetes (n = 122) against NGT (n = 796) on lipid classes adjusted for age, sex, waist circumference and SBP.

^c^ Odds ratio (95% confidence intervals) based on an interquartile range increase in predictor lipid class measurement.

^d^ Benjamini-Hochberg corrected p-value. Bold type indicates corrected p < 0.05.

^e^ % difference in means between groups.

**Table S5. Logistic regression of lipids against diabetes and prediabetes in the SAFHS cohort.**

|  |  | **Diabetes vs. NGT^a^** | | |  | **Prediabetes vs. NGT^b^** | | |
| --- | --- | --- | --- | --- | --- | --- | --- | --- |
| **Lipid Species** |  | **Odds Ratio^c^** | **p-value^d^** | **% difference^e^** |  | **Odds Ratio^c^** | **p-value^d^** | **% difference^e^** |
| dhCer 16:0 |  | 1.21 (1.01-1.45) | 5.86E-02 | 21.6 |  | 1.06 (0.89-1.25) | 6.18E-01 | 13.5 |
| dhCer 18:0 |  | 1.75 (1.38-2.23) | **2.12E-05** | 38.4 |  | 1.24 (1.00-1.55) | 8.27E-02 | 22.7 |
| dhCer 20:0 |  | 1.85 (1.38-2.47) | **1.16E-04** | 29.5 |  | 1.53 (1.19-1.96) | **3.08E-03** | 23.0 |
| dhCer 22:0 |  | 1.80 (1.39-2.34) | **4.00E-05** | 30.3 |  | 1.28 (1.02-1.62) | 6.62E-02 | 18.0 |
| dhCer 24:0 |  | 1.64 (1.29-2.08) | **1.70E-04** | 27.7 |  | 1.22 (0.98-1.52) | 1.34E-01 | 15.1 |
| dhCer 24:1 |  | 1.68 (1.33-2.12) | **5.10E-05** | 37.9 |  | 1.32 (1.06-1.64) | **2.63E-02** | 24.2 |
| Cer 16:0 |  | 2.08 (1.55-2.78) | **4.70E-06** | 23.8 |  | 1.34 (1.04-1.72) | **4.43E-02** | 12.3 |
| Cer 18:0 |  | 1.89 (1.44-2.48) | **1.83E-05** | 33.7 |  | 1.45 (1.14-1.83) | **6.83E-03** | 22.7 |
| Cer 20:0 |  | 1.55 (1.17-2.06) | **4.65E-03** | 24.1 |  | 1.51 (1.18-1.94) | **4.51E-03** | 18.7 |
| Cer 22:0 |  | 1.56 (1.24-1.97) | **4.61E-04** | 20.1 |  | 1.31 (1.06-1.62) | **2.57E-02** | 12.9 |
| Cer 24:0 |  | 1.52 (1.14-2.01) | **7.61E-03** | 12.9 |  | 1.26 (0.99-1.62) | 1.10E-01 | 7.9 |
| Cer 24:1 |  | 1.64 (1.22-2.20) | **2.37E-03** | 25.0 |  | 1.40 (1.08-1.80) | **2.33E-02** | 15.6 |
| MHC 16:0 |  | 1.23 (0.91-1.66) | 2.30E-01 | 8.7 |  | 1.10 (0.85-1.44) | 5.53E-01 | 5.0 |
| MHC 18:0 |  | 1.22 (0.96-1.54) | 1.43E-01 | 15.2 |  | 0.87 (0.68-1.12) | 3.83E-01 | 2.6 |
| MHC 20:0 |  | 1.43 (1.12-1.81) | **7.10E-03** | 22.5 |  | 1.00 (0.78-1.26) | 9.77E-01 | 5.5 |
| MHC 22:0 |  | 1.30 (0.99-1.70) | 9.14E-02 | 10.6 |  | 1.01 (0.78-1.30) | 9.77E-01 | 1.7 |
| MHC 24:0 |  | 1.51 (1.14-1.99) | **7.10E-03** | 15.7 |  | 1.09 (0.84-1.41) | 6.12E-01 | 3.4 |
| MHC 24:1 |  | 1.06 (0.80-1.41) | 7.48E-01 | 8.5 |  | 0.87 (0.66-1.14) | 4.09E-01 | -0.6 |
| DHC 16:0 |  | 0.75 (0.53-1.06) | 1.44E-01 | -3.7 |  | 0.77 (0.58-1.02) | 1.21E-01 | -4.2 |
| DHC 18:0 |  | 1.40 (1.07-1.84) | **2.64E-02** | 17.2 |  | 0.91 (0.71-1.16) | 5.34E-01 | 3.2 |
| DHC 20:0 |  | 1.26 (0.94-1.68) | 1.66E-01 | 10.2 |  | 1.15 (0.91-1.45) | 3.46E-01 | 7.3 |
| DHC 22:0 |  | 1.30 (0.99-1.71) | 8.76E-02 | 7.4 |  | 1.17 (0.94-1.47) | 2.40E-01 | 5.3 |
| DHC 24:0 |  | 1.48 (1.13-1.94) | **8.05E-03** | 10.4 |  | 1.11 (0.88-1.42) | 4.81E-01 | 2.8 |
| DHC 24:1 |  | 0.83 (0.60-1.13) | 2.99E-01 | 0.5 |  | 0.75 (0.57-0.99) | 7.45E-02 | -4.8 |
| THC 16:0 |  | 0.68 (0.50-0.91) | **1.96E-02** | 0.6 |  | 0.79 (0.61-1.01) | 9.51E-02 | -0.2 |
| THC 18:0 |  | 0.81 (0.62-1.05) | 1.56E-01 | 6.7 |  | 0.85 (0.67-1.09) | 2.87E-01 | 2.9 |
| THC 20:0 |  | 0.86 (0.65-1.14) | 3.49E-01 | 6.2 |  | 0.84 (0.66-1.07) | 2.31E-01 | 0.6 |
| THC 22:0 |  | 0.77 (0.57-1.03) | 1.23E-01 | -0.2 |  | 0.82 (0.63-1.07) | 2.12E-01 | -2.2 |
| THC 24:0 |  | 0.90 (0.68-1.20) | 5.46E-01 | 4.1 |  | 0.83 (0.65-1.05) | 1.95E-01 | -2.3 |
| THC 24:1 |  | 0.75 (0.57-1.00) | 7.42E-02 | 6.1 |  | 0.75 (0.58-0.97) | 5.64E-02 | -0.1 |
| GM3 16:0 |  | 0.89 (0.66-1.20) | 5.12E-01 | 2.8 |  | 0.85 (0.66-1.11) | 3.14E-01 | -0.5 |
| GM3 18:0 |  | 0.80 (0.57-1.12) | 2.48E-01 | 0.5 |  | 0.76 (0.57-1.02) | 1.12E-01 | -3.3 |
| GM3 20:0 |  | 1.43 (1.10-1.86) | **1.46E-02** | 16.6 |  | 1.06 (0.83-1.36) | 7.16E-01 | 5.5 |
| GM3 22:0 |  | 1.69 (1.28-2.22) | **5.00E-04** | 17.5 |  | 1.06 (0.83-1.35) | 7.28E-01 | 3.2 |
| GM3 24:0 |  | 1.08 (0.80-1.47) | 6.56E-01 | 0.4 |  | 0.83 (0.63-1.09) | 2.66E-01 | -7.2 |
| GM3 24:1 |  | 1.10 (0.83-1.45) | 5.58E-01 | 12.6 |  | 0.84 (0.65-1.09) | 2.74E-01 | -0.5 |
| SM 32:1 |  | 1.41 (1.05-1.90) | **2.20E-02** | 14.1 |  | 1.34 (1.03-1.72) | 5.19E-02 | 12.9 |
| SM 33:1 |  | 0.88 (0.65-1.19) | 4.13E-01 | 4.8 |  | 1.00 (0.77-1.30) | 9.89E-01 | 5.9 |
| SM 34:1 |  | 0.76 (0.54-1.06) | 1.10E-01 | 3.3 |  | 0.84 (0.63-1.11) | 3.00E-01 | 2.4 |
| SM 34:2 |  | 0.94 (0.67-1.31) | 7.08E-01 | 9.0 |  | 0.91 (0.68-1.22) | 6.18E-01 | 7.7 |
| SM 36:1 |  | 1.17 (0.86-1.61) | 3.21E-01 | 9.0 |  | 1.01 (0.78-1.32) | 9.54E-01 | 6.4 |
| SM 36:2 |  | 0.72 (0.51-1.03) | 7.37E-02 | 0.9 |  | 0.80 (0.60-1.07) | 2.07E-01 | 3.5 |
| SM 37:2 |  | 0.40 (0.27-0.58) | **2.37E-06** | -11.3 |  | 0.68 (0.50-0.90) | **2.03E-02** | -1.9 |
| SM 38:1 |  | 0.76 (0.54-1.07) | 1.21E-01 | -3.5 |  | 0.89 (0.67-1.18) | 5.24E-01 | -0.5 |
| SM 38:2 |  | 0.48 (0.33-0.70) | **1.66E-04** | -7.3 |  | 0.73 (0.54-0.98) | 6.56E-02 | -1.1 |
| SM 39:1 |  | 0.80 (0.56-1.15) | 2.33E-01 | -2.7 |  | 0.94 (0.70-1.27) | 7.66E-01 | 1.8 |
| SM 41:1 |  | 1.20 (0.88-1.63) | 2.62E-01 | 6.2 |  | 1.04 (0.79-1.37) | 8.41E-01 | 4.0 |
| SM 42:1 |  | 1.14 (0.84-1.55) | 3.95E-01 | 3.4 |  | 1.08 (0.82-1.43) | 6.69E-01 | 2.7 |
| PC 28:0 |  | 1.21 (1.09-1.34) | **8.76E-04** | 40.5 |  | 1.12 (1.01-1.24) | 5.93E-02 | 20.9 |
| PC 29:0 |  | 1.15 (0.85-1.56) | 4.23E-01 | 4.8 |  | 1.49 (1.17-1.91) | **4.96E-03** | 13.0 |
| PC 31:0 |  | 1.52 (1.15-2.01) | **6.68E-03** | 15.1 |  | 1.36 (1.05-1.75) | **3.70E-02** | 12.3 |
| PC 31:1 |  | 0.76 (0.55-1.04) | 1.29E-01 | 0.8 |  | 0.94 (0.73-1.22) | 7.26E-01 | 3.6 |
| PC 32:0 |  | 1.84 (1.41-2.40) | **2.61E-05** | 15.1 |  | 1.11 (0.86-1.44) | 5.11E-01 | 4.5 |
| PC 32:1 |  | 2.40 (1.80-3.19) | **2.19E-08** | 41.5 |  | 1.67 (1.29-2.16) | **5.06E-04** | 22.0 |
| PC 32:2 |  | 1.56 (1.09-2.23) | **2.57E-02** | 9.8 |  | 1.55 (1.15-2.10) | **1.23E-02** | 11.0 |
| PC 33:0 |  | 0.84 (0.60-1.17) | 3.62E-01 | -0.6 |  | 0.88 (0.67-1.16) | 4.58E-01 | -0.6 |
| PC 33:1 |  | 1.12 (0.81-1.55) | 5.36E-01 | 5.6 |  | 1.14 (0.87-1.49) | 4.40E-01 | 5.9 |
| PC 33:2 |  | 0.88 (0.64-1.20) | 4.69E-01 | -2.1 |  | 1.16 (0.89-1.52) | 3.57E-01 | 3.6 |
| PC 34:0 |  | 1.30 (0.96-1.75) | 1.28E-01 | 7.8 |  | 1.11 (0.84-1.45) | 5.60E-01 | 4.0 |
| PC 34:1 |  | 1.78 (1.30-2.43) | **7.31E-04** | 10.2 |  | 1.40 (1.07-1.84) | **3.07E-02** | 6.0 |
| PC 34:2 |  | 1.36 (1.01-1.83) | 7.22E-02 | 4.0 |  | 1.28 (0.98-1.66) | 1.13E-01 | 2.7 |
| PC 34:3 |  | 1.72 (1.25-2.38) | **2.26E-03** | 11.1 |  | 1.42 (1.08-1.85) | **2.50E-02** | 8.4 |
| PC 34:5 |  | 1.53 (1.21-1.92) | **8.26E-04** | 36.4 |  | 1.34 (1.09-1.64) | **1.39E-02** | 27.5 |
| PC 35:0 |  | 0.79 (0.57-1.09) | 1.99E-01 | -3.3 |  | 0.90 (0.69-1.17) | 5.26E-01 | -0.5 |
| PC 35:1 |  | 0.88 (0.63-1.22) | 4.97E-01 | 0.0 |  | 0.91 (0.69-1.20) | 5.86E-01 | 0.1 |
| PC 35:2 |  | 0.55 (0.39-0.79) | **2.26E-03** | -10.0 |  | 0.64 (0.48-0.85) | **7.35E-03** | -8.0 |
| PC 35:3 |  | 0.79 (0.57-1.09) | 2.01E-01 | -2.6 |  | 1.22 (0.94-1.57) | 1.99E-01 | 5.2 |
| PC 35:4 |  | 1.18 (0.87-1.60) | 3.49E-01 | 4.4 |  | 1.34 (1.05-1.71) | **4.08E-02** | 9.9 |
| PC 36:1 |  | 1.74 (1.29-2.35) | **6.66E-04** | 13.0 |  | 1.56 (1.20-2.04) | **3.93E-03** | 9.9 |
| PC 36:2 |  | 1.19 (0.88-1.60) | 3.20E-01 | 3.8 |  | 1.31 (1.01-1.70) | 7.97E-02 | 4.0 |
| PC 36:3 |  | 1.12 (0.81-1.54) | 5.52E-01 | 1.9 |  | 1.60 (1.25-2.06) | **1.07E-03** | 5.8 |
| PC 36:4 |  | 0.91 (0.66-1.24) | 5.94E-01 | -6.2 |  | 1.01 (0.77-1.33) | 9.61E-01 | -3.2 |
| PC 36:4b |  | 1.60 (1.18-2.15) | **4.71E-03** | 10.1 |  | 1.37 (1.07-1.75) | **2.85E-02** | 7.8 |
| PC 36:5 |  | 1.51 (1.18-1.93) | **2.63E-03** | 28.1 |  | 1.27 (1.03-1.55) | **4.49E-02** | 17.5 |
| PC 37:4 |  | 0.68 (0.49-0.94) | **3.38E-02** | -9.0 |  | 0.88 (0.67-1.15) | 4.47E-01 | -4.1 |
| PC 37:5 |  | 1.06 (0.80-1.41) | 7.31E-01 | 3.6 |  | 1.17 (0.92-1.49) | 2.75E-01 | 8.9 |
| PC 37:6 |  | 0.83 (0.61-1.14) | 3.23E-01 | -1.9 |  | 1.08 (0.84-1.39) | 6.18E-01 | 4.1 |
| PC 38:2 |  | 0.91 (0.66-1.24) | 5.86E-01 | 3.2 |  | 1.41 (1.09-1.82) | **1.99E-02** | 10.5 |
| PC 38:3 |  | 1.67 (1.21-2.30) | **3.54E-03** | 20.3 |  | 2.27 (1.69-3.04) | **3.21E-06** | 25.6 |
| PC 38:4 |  | 1.38 (1.04-1.84) | **4.20E-02** | 10.7 |  | 1.34 (1.05-1.70) | **3.57E-02** | 9.6 |
| PC 38:5 |  | 0.95 (0.68-1.31) | 7.88E-01 | 0.4 |  | 1.11 (0.85-1.45) | 5.34E-01 | 3.0 |
| PC 38:6a |  | 1.37 (1.02-1.86) | 6.24E-02 | 3.6 |  | 1.34 (1.03-1.74) | 5.57E-02 | 4.9 |
| PC 38:6b |  | 1.01 (0.74-1.36) | 9.83E-01 | 4.1 |  | 1.06 (0.82-1.36) | 7.28E-01 | 3.0 |
| PC 40:5 |  | 1.72 (1.27-2.32) | **1.15E-03** | 18.8 |  | 1.61 (1.23-2.11) | **1.88E-03** | 16.1 |
| PC 40:6 |  | 1.36 (1.03-1.79) | **4.84E-02** | 18.5 |  | 1.28 (1.01-1.64) | 7.90E-02 | 12.8 |
| PC 40:7 |  | 0.59 (0.41-0.85) | **9.06E-03** | -7.4 |  | 0.85 (0.65-1.13) | 3.65E-01 | -2.2 |
| PC(O-30:0) |  | 0.77 (0.54-1.08) | 1.77E-01 | -7.6 |  | 0.97 (0.74-1.26) | 8.41E-01 | -2.7 |
| PC(O-32:0) |  | 0.68 (0.49-0.95) | **4.28E-02** | -5.7 |  | 0.81 (0.62-1.07) | 2.05E-01 | -3.6 |
| PC(O-32:1) |  | 0.49 (0.34-0.71) | **4.63E-04** | -16.5 |  | 0.64 (0.49-0.85) | **6.83E-03** | -10.3 |
| PC(O-34:0) |  | 0.74 (0.52-1.03) | 1.14E-01 | -3.9 |  | 1.06 (0.80-1.39) | 7.63E-01 | 0.4 |
| PC(O-34:1) |  | 0.47 (0.33-0.67) | **1.14E-04** | -9.3 |  | 0.65 (0.49-0.85) | **6.86E-03** | -6.3 |
| PC(O-34:2) |  | 0.50 (0.33-0.75) | **1.69E-03** | -16.9 |  | 0.81 (0.62-1.07) | 2.05E-01 | -8.3 |
| PC(O-36:0) |  | 1.00 (0.78-1.30) | 9.83E-01 | 0.4 |  | 1.15 (0.93-1.43) | 2.81E-01 | 4.2 |
| PC(O-36:1) |  | 0.81 (0.60-1.09) | 2.28E-01 | -2.2 |  | 0.80 (0.62-1.04) | 1.52E-01 | -4.2 |
| PC(O-36:2) |  | 0.58 (0.41-0.83) | **6.13E-03** | -13.1 |  | 0.64 (0.48-0.86) | **8.78E-03** | -10.9 |
| PC(O-36:3) |  | 0.62 (0.44-0.90) | **1.90E-02** | -12.0 |  | 1.02 (0.78-1.33) | 9.22E-01 | -3.1 |
| PC(O-36:4) |  | 0.98 (0.71-1.35) | 9.20E-01 | -4.2 |  | 0.94 (0.72-1.23) | 7.28E-01 | -3.1 |
| PC(O-36:5) |  | 1.07 (0.78-1.46) | 7.17E-01 | 0.7 |  | 0.89 (0.68-1.16) | 4.92E-01 | -2.2 |
| PC(O-38:4) |  | 1.05 (0.79-1.41) | 7.78E-01 | -0.9 |  | 0.91 (0.70-1.18) | 5.72E-01 | -3.1 |
| PC(O-38:5) |  | 0.78 (0.56-1.08) | 1.81E-01 | -6.4 |  | 0.82 (0.62-1.08) | 2.41E-01 | -4.9 |
| PC(O-40:7) |  | 0.95 (0.72-1.27) | 7.92E-01 | 1.5 |  | 0.81 (0.63-1.05) | 1.85E-01 | -2.6 |
| PC(P-32:0) |  | 0.54 (0.39-0.76) | **8.53E-04** | -5.9 |  | 0.66 (0.50-0.86) | **6.11E-03** | -4.6 |
| PC(P-34:1) |  | 0.34 (0.23-0.50) | **2.51E-07** | -13.1 |  | 0.58 (0.44-0.76) | **5.06E-04** | -7.4 |
| PC(P-36:2) |  | 0.54 (0.37-0.80) | **4.57E-03** | -15.5 |  | 0.61 (0.45-0.83) | **6.10E-03** | -12.3 |
| PC(P-36:5) |  | 1.11 (0.89-1.39) | 4.27E-01 | 4.1 |  | 0.98 (0.79-1.20) | 8.60E-01 | -0.1 |
| PC(P-38:5) |  | 0.91 (0.67-1.25) | 6.24E-01 | -3.2 |  | 0.77 (0.59-1.01) | 1.06E-01 | -4.2 |
| LPC 14:0 |  | 1.55 (1.19-2.01) | **2.57E-03** | 15.5 |  | 1.26 (0.98-1.61) | 1.16E-01 | 8.1 |
| LPC 15:0 |  | 0.57 (0.40-0.81) | **3.96E-03** | -9.8 |  | 0.71 (0.53-0.95) | **4.08E-02** | -7.1 |
| LPC 16:0 |  | 1.19 (0.93-1.53) | 2.22E-01 | 8.0 |  | 1.01 (0.79-1.27) | 9.77E-01 | -0.5 |
| LPC 16:1 |  | 1.17 (0.95-1.45) | 1.84E-01 | 8.0 |  | 0.89 (0.70-1.13) | 4.58E-01 | -1.4 |
| LPC 18:0 |  | 0.85 (0.61-1.19) | 4.12E-01 | 1.4 |  | 0.83 (0.62-1.09) | 2.65E-01 | -1.4 |
| LPC 18:1 |  | 0.70 (0.48-1.02) | 1.02E-01 | -8.8 |  | 0.66 (0.48-0.92) | **3.16E-02** | -12.1 |
| LPC 18:2 |  | 0.60 (0.40-0.91) | **2.83E-02** | -15.8 |  | 0.54 (0.38-0.76) | **1.74E-03** | -17.3 |
| LPC 20:0 |  | 0.66 (0.48-0.92) | **2.38E-02** | -8.9 |  | 0.73 (0.56-0.95) | **4.38E-02** | -9.6 |
| LPC 20:1 |  | 0.45 (0.30-0.67) | **2.32E-04** | -11.5 |  | 0.63 (0.47-0.85) | **7.69E-03** | -9.7 |
| LPC 20:2 |  | 0.68 (0.48-0.97) | 5.26E-02 | -10.0 |  | 0.90 (0.70-1.16) | 5.10E-01 | -6.6 |
| LPC 20:3 |  | 1.31 (0.97-1.75) | 1.12E-01 | 3.5 |  | 1.33 (1.03-1.73) | 5.57E-02 | 4.0 |
| LPC 20:4 |  | 1.32 (0.96-1.82) | 1.28E-01 | 0.2 |  | 0.81 (0.60-1.08) | 2.13E-01 | -8.3 |
| LPC 20:5 |  | 1.16 (0.93-1.43) | 2.45E-01 | 6.3 |  | 0.96 (0.76-1.21) | 7.79E-01 | -4.5 |
| LPC 22:0 |  | 0.60 (0.43-0.85) | **8.61E-03** | -10.0 |  | 0.71 (0.54-0.94) | **3.58E-02** | -8.7 |
| LPC 22:6 |  | 1.20 (0.91-1.57) | 2.55E-01 | 4.6 |  | 0.93 (0.72-1.20) | 6.50E-01 | -6.2 |
| LPC 24:0 |  | 0.79 (0.57-1.10) | 2.24E-01 | -1.7 |  | 0.74 (0.55-0.99) | 7.40E-02 | -5.4 |
| LPC(O-22:0) |  | 0.63 (0.45-0.88) | **1.38E-02** | -8.9 |  | 0.74 (0.56-0.96) | 5.04E-02 | -8.8 |
| LPC(O-24:1) |  | 0.77 (0.55-1.06) | 1.56E-01 | -7.6 |  | 0.86 (0.66-1.12) | 3.61E-01 | -7.2 |
| LPC(O-24:2) |  | 0.49 (0.31-0.77) | **4.65E-03** | -21.1 |  | 0.68 (0.50-0.92) | **2.94E-02** | -16.9 |
| PE 32:1 |  | 1.80 (1.51-2.15) | **2.59E-09** | 121.0 |  | 1.43 (1.21-1.68) | **1.57E-04** | 52.0 |
| PE 34:1 |  | 2.21 (1.73-2.84) | **6.31E-09** | 66.9 |  | 1.51 (1.24-1.84) | **3.40E-04** | 30.5 |
| PE 34:2 |  | 2.35 (1.79-3.09) | **1.39E-08** | 57.7 |  | 1.48 (1.20-1.82) | **1.18E-03** | 29.1 |
| PE 36:1 |  | 2.65 (1.97-3.56) | **3.68E-09** | 61.4 |  | 1.84 (1.45-2.33) | **1.61E-05** | 33.7 |
| PE 36:2 |  | 2.43 (1.80-3.28) | **5.21E-08** | 51.4 |  | 1.63 (1.29-2.05) | **2.33E-04** | 28.1 |
| PE 36:3 |  | 1.81 (1.40-2.34) | **2.49E-05** | 33.0 |  | 1.38 (1.11-1.70) | **9.26E-03** | 17.6 |
| PE 36:4 |  | 2.58 (1.93-3.43) | **2.88E-09** | 50.1 |  | 1.59 (1.29-1.97) | **1.50E-04** | 28.5 |
| PE 36:5 |  | 1.90 (1.55-2.34) | **1.42E-08** | 66.0 |  | 1.38 (1.15-1.64) | **1.66E-03** | 31.7 |
| PE 38:3 |  | 2.75 (1.99-3.81) | **1.36E-08** | 48.5 |  | 1.97 (1.55-2.51) | **3.15E-06** | 35.3 |
| PE 38:4 |  | 2.73 (1.97-3.77) | **1.66E-08** | 39.2 |  | 1.79 (1.41-2.29) | **4.01E-05** | 25.8 |
| PE 38:5 |  | 2.04 (1.55-2.69) | **2.00E-06** | 31.2 |  | 1.46 (1.18-1.81) | **1.94E-03** | 19.2 |
| PE 38:6 |  | 1.88 (1.50-2.36) | **3.90E-07** | 51.7 |  | 1.38 (1.14-1.66) | **3.20E-03** | 28.2 |
| PE 40:6 |  | 2.12 (1.67-2.68) | **6.50E-09** | 65.5 |  | 1.50 (1.24-1.82) | **2.63E-04** | 37.9 |
| PE 40:7 |  | 1.36 (1.12-1.65) | **4.65E-03** | 31.7 |  | 1.20 (1.01-1.42) | 7.59E-02 | 19.1 |
| PI 32:0 |  | 1.77 (1.48-2.11) | **5.71E-09** | 74.4 |  | 1.57 (1.35-1.83) | **2.33E-06** | 61.6 |
| PI 32:1 |  | 1.71 (1.40-2.08) | **8.00E-07** | 97.2 |  | 1.61 (1.34-1.92) | **1.05E-05** | 63.3 |
| PI 34:0 |  | 1.65 (1.38-1.98) | **3.06E-07** | 44.2 |  | 1.44 (1.25-1.67) | **2.65E-05** | 33.9 |
| PI 34:1 |  | 1.77 (1.40-2.24) | **8.56E-06** | 49.5 |  | 1.63 (1.32-2.02) | **6.00E-05** | 30.0 |
| PI 36:0 |  | 1.71 (1.33-2.20) | **8.78E-05** | 48.0 |  | 1.55 (1.24-1.93) | **5.42E-04** | 28.1 |
| PI 36:1 |  | 1.51 (1.18-1.93) | **2.30E-03** | 26.4 |  | 1.36 (1.08-1.72) | **2.11E-02** | 13.0 |
| PI 36:2 |  | 1.49 (1.13-1.97) | **8.61E-03** | 16.3 |  | 1.41 (1.11-1.79) | **1.22E-02** | 11.0 |
| PI 36:3 |  | 1.19 (0.93-1.53) | 2.34E-01 | 15.0 |  | 1.36 (1.09-1.71) | **1.74E-02** | 14.3 |
| PI 36:4 |  | 1.89 (1.47-2.43) | **3.77E-06** | 45.6 |  | 1.74 (1.38-2.19) | **4.01E-05** | 34.5 |
| PI 38:2 |  | 1.27 (1.00-1.60) | 8.10E-02 | 21.1 |  | 1.25 (1.02-1.53) | 5.57E-02 | 13.3 |
| PI 38:3 |  | 1.59 (1.22-2.07) | **1.31E-03** | 31.4 |  | 1.51 (1.21-1.89) | **1.27E-03** | 23.5 |
| PI 38:4 |  | 1.84 (1.36-2.48) | **2.32E-04** | 23.2 |  | 1.36 (1.05-1.76) | **4.08E-02** | 13.3 |
| PI 38:5 |  | 1.00 (0.78-1.30) | 9.83E-01 | 8.4 |  | 1.18 (0.95-1.47) | 1.99E-01 | 12.9 |
| PI 38:6 |  | 1.46 (1.14-1.86) | **4.77E-03** | 27.3 |  | 1.46 (1.16-1.83) | **4.10E-03** | 17.5 |
| PI 40:4 |  | 1.93 (1.46-2.55) | **1.81E-05** | 31.9 |  | 1.76 (1.37-2.25) | **7.12E-05** | 23.1 |
| PI 40:5 |  | 1.58 (1.24-2.02) | **5.72E-04** | 27.0 |  | 1.29 (1.03-1.61) | **4.92E-02** | 16.4 |
| PI 40:6 |  | 1.41 (1.10-1.82) | **1.35E-02** | 21.8 |  | 1.47 (1.17-1.85) | **3.46E-03** | 15.2 |
| PS 36:1 |  | 0.98 (0.75-1.29) | 9.30E-01 | -5.1 |  | 1.23 (1.01-1.50) | 6.51E-02 | 12.9 |
| PS 36:2 |  | 0.86 (0.62-1.20) | 4.46E-01 | -14.6 |  | 1.25 (1.00-1.56) | 8.12E-02 | 9.1 |
| PS 38:3 |  | 1.10 (0.87-1.40) | 4.69E-01 | -1.0 |  | 1.18 (0.97-1.43) | 1.49E-01 | 8.6 |
| PS 38:4 |  | 0.87 (0.66-1.16) | 4.12E-01 | -12.2 |  | 1.13 (0.92-1.38) | 3.22E-01 | 4.6 |
| PS 38:5 |  | 0.82 (0.59-1.13) | 2.82E-01 | -18.5 |  | 1.18 (0.95-1.46) | 2.05E-01 | 4.5 |
| PS 40:5 |  | 0.95 (0.74-1.22) | 7.42E-01 | -11.6 |  | 1.15 (0.96-1.38) | 2.07E-01 | 10.2 |
| PS 40:6 |  | 1.06 (0.82-1.37) | 7.13E-01 | 0.0 |  | 1.10 (0.88-1.37) | 5.11E-01 | 2.0 |
| PG 34:1 |  | 2.25 (1.72-2.93) | **2.74E-08** | 52.4 |  | 1.40 (1.12-1.73) | **7.53E-03** | 23.1 |
| PG 34:2 |  | 2.34 (1.71-3.19) | **5.39E-07** | 61.6 |  | 1.43 (1.09-1.86) | **2.21E-02** | 28.9 |
| PG 36:1 |  | 2.64 (2.03-3.45) | **7.67E-11** | 89.2 |  | 1.83 (1.48-2.26) | **3.15E-06** | 49.2 |
| PG 36:2 |  | 2.57 (1.86-3.57) | **1.01E-07** | 54.6 |  | 1.63 (1.30-2.05) | **1.85E-04** | 32.3 |
| COH |  | 1.18 (0.89-1.56) | 3.16E-01 | 15.3 |  | 1.16 (0.90-1.48) | 3.50E-01 | 10.3 |
| CE 14:0 |  | 2.06 (1.62-2.62) | **3.17E-08** | 65.9 |  | 1.70 (1.36-2.11) | **4.01E-05** | 39.1 |
| CE 15:0 |  | 1.60 (1.27-2.02) | **1.78E-04** | 29.8 |  | 1.40 (1.11-1.76) | **1.21E-02** | 18.1 |
| CE 16:0 |  | 2.12 (1.62-2.77) | **2.97E-07** | 19.9 |  | 1.48 (1.16-1.90) | **5.91E-03** | 11.0 |
| CE 16:1 |  | 1.92 (1.55-2.39) | **2.74E-08** | 83.1 |  | 1.56 (1.27-1.91) | **1.50E-04** | 42.3 |
| CE 16:2 |  | 2.29 (1.77-2.96) | **5.71E-09** | 58.7 |  | 1.78 (1.42-2.24) | **1.61E-05** | 39.5 |
| CE 17:0 |  | 1.14 (0.87-1.48) | 4.04E-01 | 6.5 |  | 1.01 (0.79-1.29) | 9.54E-01 | 2.2 |
| CE 17:1 |  | 2.20 (1.62-2.98) | **2.05E-06** | 19.3 |  | 1.25 (0.97-1.61) | 1.42E-01 | 7.5 |
| CE 18:0 |  | 1.11 (0.82-1.50) | 5.49E-01 | 7.2 |  | 1.23 (0.95-1.58) | 1.74E-01 | 9.6 |
| CE 18:1 |  | 1.74 (1.32-2.29) | **2.32E-04** | 18.9 |  | 1.53 (1.19-1.98) | **3.63E-03** | 12.3 |
| CE 18:2 |  | 2.27 (1.76-2.92) | **5.71E-09** | 29.8 |  | 1.53 (1.24-1.89) | **4.10E-04** | 15.1 |
| CE 18:3 |  | 2.11 (1.66-2.69) | **1.39E-08** | 56.7 |  | 1.63 (1.32-2.02) | **7.12E-05** | 35.3 |
| CE 20:1 |  | 0.69 (0.49-0.97) | 5.45E-02 | -7.1 |  | 0.78 (0.59-1.02) | 1.16E-01 | -6.3 |
| CE 20:2 |  | 1.27 (0.96-1.68) | 1.42E-01 | 12.4 |  | 1.35 (1.06-1.71) | **3.20E-02** | 11.1 |
| CE 20:3 |  | 2.03 (1.53-2.68) | **3.62E-06** | 37.7 |  | 1.98 (1.55-2.54) | **3.47E-06** | 32.2 |
| CE 20:4 |  | 2.03 (1.59-2.58) | **7.25E-08** | 42.9 |  | 1.44 (1.17-1.77) | **1.86E-03** | 23.6 |
| CE 20:5 |  | 1.49 (1.25-1.78) | **4.27E-05** | 64.6 |  | 1.21 (1.03-1.43) | **4.08E-02** | 33.3 |
| CE 22:0 |  | 1.38 (1.14-1.67) | **2.45E-03** | 33.1 |  | 1.21 (1.01-1.45) | 6.64E-02 | 20.3 |
| CE 22:1 |  | 1.09 (0.88-1.36) | 4.69E-01 | 13.6 |  | 1.08 (0.89-1.31) | 5.40E-01 | 7.5 |
| CE 22:4 |  | 1.83 (1.36-2.46) | **2.03E-04** | 26.2 |  | 1.41 (1.07-1.85) | **2.94E-02** | 15.2 |
| CE 22:5 |  | 2.16 (1.62-2.88) | **9.20E-07** | 39.6 |  | 1.60 (1.24-2.05) | **1.04E-03** | 24.4 |
| CE 22:6 |  | 1.71 (1.36-2.16) | **2.17E-05** | 44.3 |  | 1.26 (1.02-1.55) | 5.84E-02 | 20.8 |
| CE 24:0 |  | 1.54 (1.28-1.86) | **2.07E-05** | 44.1 |  | 1.31 (1.09-1.57) | **9.32E-03** | 24.7 |
| CE 24:1 |  | 1.42 (1.14-1.76) | **3.79E-03** | 31.9 |  | 1.17 (0.94-1.46) | 2.46E-01 | 13.6 |
| CE 24:4 |  | 1.00 (0.76-1.31) | 9.87E-01 | -1.5 |  | 1.22 (0.97-1.53) | 1.44E-01 | 2.6 |
| CE 24:5 |  | 1.60 (1.26-2.03) | **3.35E-04** | 20.8 |  | 1.44 (1.16-1.80) | **3.63E-03** | 11.3 |
| CE 24:6 |  | 1.65 (1.30-2.09) | **1.29E-04** | 34.0 |  | 1.51 (1.21-1.87) | **9.97E-04** | 22.2 |
| DG 14:0/16:0 |  | 1.69 (1.43-1.99) | **6.50E-09** | 122.6 |  | 1.37 (1.18-1.58) | **1.51E-04** | 66.7 |
| DG 14:0/18:1 |  | 1.78 (1.46-2.16) | **6.70E-08** | 86.2 |  | 1.50 (1.26-1.78) | **5.44E-05** | 56.7 |
| DG 14:0/18:2 |  | 1.56 (1.30-1.86) | **6.61E-06** | 62.6 |  | 1.39 (1.19-1.63) | **2.42E-04** | 49.3 |
| DG 16:0/16:0 |  | 1.86 (1.56-2.20) | **9.33E-11** | 139.7 |  | 1.46 (1.26-1.70) | **2.46E-05** | 72.1 |
| DG 16:0/18:0 |  | 1.89 (1.59-2.26) | **8.16E-11** | 103.8 |  | 1.50 (1.28-1.76) | **1.93E-05** | 55.0 |
| DG 16:0/18:1 |  | 2.01 (1.64-2.46) | **7.95E-10** | 91.2 |  | 1.57 (1.31-1.89) | **3.43E-05** | 51.2 |
| DG 16:0/18:2 |  | 1.93 (1.55-2.41) | **4.35E-08** | 64.5 |  | 1.49 (1.24-1.80) | **1.85E-04** | 40.7 |
| DG 16:0/20:3 |  | 2.33 (1.85-2.95) | **8.49E-11** | 109.0 |  | 1.70 (1.40-2.06) | **3.24E-06** | 63.9 |
| DG 16:0/20:4 |  | 2.00 (1.67-2.41) | **5.19E-11** | 119.0 |  | 1.39 (1.18-1.64) | **4.12E-04** | 55.7 |
| DG 16:0/22:5 |  | 2.01 (1.62-2.48) | **4.16E-09** | 92.2 |  | 1.53 (1.28-1.83) | **5.24E-05** | 48.4 |
| DG 16:0/22:6 |  | 1.71 (1.42-2.04) | **5.78E-08** | 122.5 |  | 1.41 (1.20-1.65) | **1.71E-04** | 60.0 |
| DG 16:1/18:0 |  | 1.68 (1.42-1.98) | **1.39E-08** | 128.6 |  | 1.44 (1.23-1.69) | **5.31E-05** | 68.5 |
| DG 16:1/18:1 |  | 1.70 (1.40-2.05) | **3.01E-07** | 86.1 |  | 1.46 (1.22-1.75) | **2.42E-04** | 50.8 |
| DG 18:0/18:1 |  | 1.61 (1.37-1.89) | **6.54E-08** | 78.0 |  | 1.36 (1.17-1.59) | **3.40E-04** | 45.2 |
| DG 18:0/18:2 |  | 1.58 (1.33-1.89) | **1.42E-06** | 56.4 |  | 1.37 (1.17-1.61) | **5.42E-04** | 38.5 |
| DG 18:0/20:4 |  | 2.07 (1.67-2.57) | **1.85E-09** | 60.6 |  | 1.43 (1.19-1.72) | **7.52E-04** | 32.3 |
| DG 18:1/18:1 |  | 1.63 (1.32-2.00) | **1.83E-05** | 57.2 |  | 1.41 (1.18-1.69) | **9.06E-04** | 37.3 |
| DG 18:1/18:2 |  | 1.45 (1.15-1.82) | **3.34E-03** | 37.5 |  | 1.34 (1.11-1.62) | **7.23E-03** | 27.8 |
| DG 18:1/18:3 |  | 1.30 (1.07-1.58) | **1.55E-02** | 35.7 |  | 1.24 (1.04-1.47) | **3.20E-02** | 25.3 |
| DG 18:1/20:3 |  | 1.61 (1.31-1.97) | **1.85E-05** | 57.6 |  | 1.44 (1.21-1.72) | **2.42E-04** | 40.1 |
| DG 18:1/20:4 |  | 1.78 (1.46-2.17) | **1.26E-07** | 71.3 |  | 1.32 (1.11-1.57) | **6.20E-03** | 36.7 |
| DG 18:2/18:2 |  | 1.14 (0.93-1.39) | 2.82E-01 | 20.4 |  | 1.19 (1.00-1.42) | 8.96E-02 | 18.6 |
| TG 14:0/16:0/18:1 |  | 2.12 (1.69-2.65) | **2.59E-09** | 96.6 |  | 1.66 (1.36-2.04) | **2.65E-05** | 56.8 |
| TG 14:0/16:0/18:2 |  | 1.96 (1.59-2.41) | **5.86E-09** | 108.3 |  | 1.57 (1.30-1.90) | **4.01E-05** | 61.2 |
| TG 14:0/16:1/18:1 |  | 1.63 (1.35-1.97) | **2.05E-06** | 70.2 |  | 1.42 (1.19-1.69) | **5.06E-04** | 47.2 |
| TG 14:0/16:1/18:2 |  | 1.38 (1.16-1.63) | **5.95E-04** | 42.1 |  | 1.32 (1.13-1.54) | **1.56E-03** | 45.7 |
| TG 14:0/17:0/18:1 |  | 1.73 (1.42-2.11) | **3.96E-07** | 62.3 |  | 1.47 (1.21-1.77) | **4.19E-04** | 41.2 |
| TG 14:0/18:0/18:1 |  | 1.83 (1.52-2.21) | **3.76E-09** | 88.9 |  | 1.52 (1.27-1.81) | **4.20E-05** | 50.7 |
| TG 14:0/18:2/18:2 |  | 1.09 (0.87-1.38) | 4.97E-01 | 13.5 |  | 1.28 (1.08-1.52) | **1.37E-02** | 27.9 |
| TG 14:1/16:0/18:1 |  | 1.80 (1.48-2.18) | **2.44E-08** | 99.2 |  | 1.49 (1.24-1.80) | **1.85E-04** | 53.1 |
| TG 14:1/16:1/18:0 |  | 1.70 (1.44-2.01) | **6.50E-09** | 146.1 |  | 1.43 (1.21-1.68) | **1.60E-04** | 69.7 |
| TG 14:1/18:0/18:2 |  | 1.59 (1.32-1.93) | **7.98E-06** | 54.7 |  | 1.44 (1.21-1.71) | **2.42E-04** | 42.8 |
| TG 14:1/18:1/18:1 |  | 1.63 (1.29-2.06) | **1.25E-04** | 43.9 |  | 1.51 (1.23-1.85) | **4.43E-04** | 36.5 |
| TG 15:0/16:0/18:1 |  | 1.95 (1.58-2.41) | **6.50E-09** | 77.2 |  | 1.56 (1.29-1.89) | **5.31E-05** | 50.7 |
| TG 15:0/18:1/18:1 |  | 1.28 (1.03-1.60) | **4.64E-02** | 28.1 |  | 1.32 (1.08-1.61) | **1.59E-02** | 25.8 |
| TG 16:0/16:0/16:0 |  | 1.86 (1.57-2.19) | **5.19E-11** | 177.0 |  | 1.46 (1.26-1.69) | **1.73E-05** | 85.6 |
| TG 16:0/16:0/18:0 |  | 1.77 (1.52-2.06) | **5.19E-11** | 147.0 |  | 1.40 (1.22-1.61) | **4.01E-05** | 64.6 |
| TG 16:0/16:0/18:1 |  | 2.61 (2.02-3.38) | **5.19E-11** | 116.9 |  | 1.80 (1.47-2.22) | **3.15E-06** | 61.7 |
| TG 16:0/16:0/18:2 |  | 2.89 (2.17-3.85) | **5.19E-11** | 90.9 |  | 1.82 (1.47-2.25) | **3.15E-06** | 54.6 |
| TG 16:0/16:1/17:0 |  | 2.07 (1.68-2.57) | **7.95E-10** | 94.6 |  | 1.59 (1.31-1.93) | **4.64E-05** | 54.1 |
| TG 16:0/16:1/18:1 |  | 2.09 (1.65-2.66) | **1.78E-08** | 78.6 |  | 1.71 (1.37-2.14) | **4.01E-05** | 48.2 |
| TG 16:0/17:0/18:0 |  | 1.52 (1.33-1.75) | **1.78E-08** | 115.3 |  | 1.29 (1.13-1.48) | **1.12E-03** | 52.5 |
| TG 16:0/17:0/18:1 |  | 1.92 (1.57-2.35) | **5.86E-09** | 82.0 |  | 1.51 (1.25-1.83) | **1.58E-04** | 45.7 |
| TG 16:0/17:0/18:2 |  | 2.03 (1.59-2.59) | **9.88E-08** | 65.3 |  | 1.60 (1.29-1.99) | **1.50E-04** | 42.4 |
| TG 16:0/18:0/18:1 |  | 1.90 (1.60-2.26) | **5.47E-11** | 105.0 |  | 1.49 (1.27-1.75) | **2.65E-05** | 53.4 |
| TG 16:0/18:1/18:1 |  | 2.17 (1.67-2.83) | **6.72E-08** | 56.1 |  | 1.66 (1.33-2.08) | **8.70E-05** | 37.5 |
| TG 16:0/18:1/18:2 |  | 1.68 (1.31-2.16) | **1.35E-04** | 34.6 |  | 1.48 (1.21-1.82) | **7.88E-04** | 30.0 |
| TG 16:0/18:2/18:2 |  | 1.28 (1.03-1.60) | **4.42E-02** | 24.1 |  | 1.30 (1.07-1.57) | **1.71E-02** | 24.5 |
| TG 16:1/16:1/16:1 |  | 1.67 (1.37-2.03) | **1.74E-06** | 95.0 |  | 1.48 (1.23-1.77) | **1.85E-04** | 56.2 |
| TG 16:1/16:1/18:0 |  | 1.89 (1.56-2.28) | **1.95E-09** | 87.0 |  | 1.53 (1.28-1.82) | **4.01E-05** | 48.9 |
| TG 16:1/16:1/18:1 |  | 2.03 (1.60-2.57) | **4.01E-08** | 73.4 |  | 1.67 (1.36-2.06) | **2.65E-05** | 51.0 |
| TG 16:1/17:0/18:1 |  | 1.61 (1.29-2.01) | **1.14E-04** | 46.1 |  | 1.43 (1.16-1.76) | **2.57E-03** | 32.6 |
| TG 16:1/18:1/18:1 |  | 1.56 (1.23-1.97) | **5.92E-04** | 43.8 |  | 1.42 (1.14-1.76) | **5.64E-03** | 31.4 |
| TG 16:1/18:1/18:2 |  | 1.34 (1.04-1.73) | **4.32E-02** | 26.6 |  | 1.35 (1.10-1.66) | **1.28E-02** | 26.4 |
| TG 17:0/18:1/18:1 |  | 1.48 (1.22-1.81) | **3.26E-04** | 43.2 |  | 1.34 (1.11-1.61) | **6.11E-03** | 30.2 |
| TG 18:0/18:0/18:1 |  | 1.24 (1.13-1.35) | **2.19E-05** | 102.0 |  | 1.14 (1.04-1.24) | **1.47E-02** | 44.7 |
| TG 18:0/18:1/18:1 |  | 1.45 (1.26-1.67) | **9.65E-07** | 65.3 |  | 1.28 (1.12-1.47) | **1.56E-03** | 38.6 |
| TG 18:0/18:2/18:2 |  | 1.08 (0.93-1.24) | 3.85E-01 | 23.2 |  | 1.09 (0.95-1.24) | 3.14E-01 | 19.7 |
| TG 18:1/18:1/18:1 |  | 1.30 (1.07-1.58) | **1.62E-02** | 31.7 |  | 1.25 (1.06-1.48) | **1.98E-02** | 25.7 |
| TG 18:1/18:1/18:2 |  | 1.14 (0.89-1.46) | 3.50E-01 | 15.5 |  | 1.18 (0.97-1.43) | 1.44E-01 | 17.1 |
| TG 18:1/18:1/20:4 |  | 1.72 (1.34-2.20) | **8.48E-05** | 35.5 |  | 1.29 (1.05-1.58) | **3.17E-02** | 22.1 |
| TG 18:1/18:1/22:6 |  | 1.92 (1.48-2.50) | **5.23E-06** | 40.4 |  | 1.32 (1.08-1.61) | **1.71E-02** | 23.8 |
| TG 18:1/18:2/18:2 |  | 0.97 (0.76-1.24) | 8.47E-01 | 6.8 |  | 1.10 (0.91-1.31) | 4.29E-01 | 12.1 |
| TG 18:2/18:2/18:2 |  | 0.88 (0.70-1.11) | 3.53E-01 | -4.3 |  | 1.02 (0.86-1.21) | 8.41E-01 | 4.6 |
| TG 18:2/18:2/20:4 |  | 1.14 (0.97-1.34) | 1.52E-01 | 20.5 |  | 1.00 (0.85-1.19) | 9.77E-01 | 5.7 |

^a^ Logistic regression of T2D (n = 69) against NGT (n = 796) on lipid species adjusted for age, sex, waist circumference and SBP.

^b^ Logistic regression of prediabetes (n = 122) against NGT (n = 796) on lipid species adjusted for age, sex, waist circumference and SBP.

^c^ Odds ratio (95% confidence intervals) based on an interquartile range increase in predictor lipid species measurement.

^d^ Benjamini-Hochberg corrected p-value. Bold type indicates corrected p < 0.05.

^e^ % difference in means between groups.

**Table S6. Linear regression of lipid species in the AusDiab and SAFHS cohorts.**

| **111111111** |  | **FPG**^a^ **(AusDiab)** | |  | **2h-PLG**^b^ **(AusDiab)** | |  | **FPG**^a^ **(SAFHS)** | |  | **2h-PLG**^b^ **(SAFHS)** | |
| --- | --- | --- | --- | --- | --- | --- | --- | --- | --- | --- | --- | --- |
| **Lipid Species** |  | **Beta Coeff.**^c^ | **p-value**^d^ |  | **Beta Coeff.**^c^ | **p-value**^d^ |  | **Beta Coeff.**^c^ | **p-value**^d^ |  | **Beta Coeff.**^c^ | **p-value**^d^ |
| dhCer 16:0 |  | 0.14 (0.00 - 0.28) | 1.51E-01 |  | 0.36 (-0.09 - 0.82) | 1.79E-01 |  | 0.28 (0.14 - 0.41) | **2.58E-04** |  | 0.50 (0.22 - 0.77) | **8.84E-04** |
| dhCer 18:0 |  | 0.21 (0.08 - 0.34) | **1.32E-02** |  | 0.80 (0.39 - 1.21) | **1.33E-03** |  | 0.58 (0.42 - 0.73) | **4.23E-11** |  | 1.16 (0.85 - 1.48) | **7.28E-12** |
| dhCer 20:0 |  | 0.11 (-0.03 - 0.25) | 2.49E-01 |  | 0.35 (-0.09 - 0.80) | 1.86E-01 |  | 0.55 (0.37 - 0.73) | **2.00E-08** |  | 1.17 (0.81 - 1.52) | **1.20E-09** |
| dhCer 22:0 |  | 0.22 (0.09 - 0.34) | **8.44E-03** |  | 0.47 (0.07 - 0.87) | 5.15E-02 |  | 0.58 (0.41 - 0.75) | **3.18E-10** |  | 1.21 (0.87 - 1.55) | **2.71E-11** |
| dhCer 24:0 |  | 0.15 (0.04 - 0.27) | **3.80E-02** |  | 0.26 (-0.09 - 0.61) | 2.16E-01 |  | 0.47 (0.30 - 0.64) | **2.57E-07** |  | 0.99 (0.66 - 1.33) | **2.94E-08** |
| dhCer 24:1 |  | 0.16 (0.03 - 0.29) | 7.19E-02 |  | 0.50 (0.09 - 0.91) | **4.19E-02** |  | 0.41 (0.25 - 0.57) | **2.66E-06** |  | 0.97 (0.65 - 1.30) | **1.43E-08** |
| Cer 16:0 |  | 0.06 (-0.10 - 0.22) | 5.88E-01 |  | 0.25 (-0.26 - 0.75) | 4.38E-01 |  | 6890 | **3.51E-07** |  | 1.38 (1.01 - 1.76) | **9.86E-12** |
| Cer 18:0 |  | 0.17 (0.02 - 0.33) | 9.31E-02 |  | 1.02 (0.54 - 1.50) | **5.74E-04** |  | 0.55 (0.37 - 0.73) | **2.01E-08** |  | 1.32 (0.96 - 1.67) | **5.75E-12** |
| Cer 20:0 |  | 0.12 (-0.03 - 0.27) | 2.23E-01 |  | 0.73 (0.25 - 1.20) | **1.11E-02** |  | 0.38 (0.20 - 0.56) | **1.52E-04** |  | 1.00 (0.64 - 1.36) | **2.38E-07** |
| Cer 22:0 |  | 0.11 (-0.04 - 0.25) | 2.63E-01 |  | 0.61 (0.16 - 1.07) | **2.64E-02** |  | 0.57 (0.40 - 0.74) | **5.83E-10** |  | 1.32 (0.99 - 1.66) | **1.44E-13** |
| Cer 24:0 |  | 0.04 (-0.12 - 0.20) | 7.06E-01 |  | 0.01 (-0.49 - 0.51) | 9.75E-01 |  | 0.47 (0.30 - 0.65) | **7.33E-07** |  | 1.13 (0.78 - 1.48) | **1.44E-09** |
| Cer 24:1 |  | 0.02 (-0.13 - 0.18) | 8.10E-01 |  | 0.51 (0.04 - 0.98) | 7.25E-02 |  | 0.38 (0.20 - 0.57) | **2.05E-04** |  | 1.08 (0.71 - 1.45) | **4.89E-08** |
| MHC 16:0 |  | -0.18 (-0.33 - -0.03) | 7.27E-02 |  | -0.33 (-0.80 - 0.15) | 2.59E-01 |  | -0.14 (-0.32 - 0.04) | 1.95E-01 |  | -0.15 (-0.51 - 0.22) | 5.05E-01 |
| MHC 18:0 |  | -0.08 (-0.23 - 0.07) | 4.51E-01 |  | -0.10 (-0.57 - 0.37) | 7.49E-01 |  | 0.23 (0.06 - 0.40) | **1.71E-02** |  | 0.37 (0.03 - 0.71) | 5.08E-02 |
| MHC 20:0 |  | -0.08 (-0.23 - 0.08) | 4.86E-01 |  | 0.11 (-0.37 - 0.60) | 7.20E-01 |  | 0.05 (-0.12 - 0.22) | 6.14E-01 |  | 0.19 (-0.15 - 0.53) | 3.37E-01 |
| MHC 22:0 |  | -0.15 (-0.31 - 0.00) | 1.47E-01 |  | -0.15 (-0.64 - 0.33) | 6.19E-01 |  | 0.02 (-0.15 - 0.19) | 8.37E-01 |  | 0.05 (-0.29 - 0.39) | 7.98E-01 |
| MHC 24:0 |  | -0.15 (-0.30 - 0.01) | 1.67E-01 |  | -0.17 (-0.66 - 0.32) | 5.86E-01 |  | 0.00 (-0.18 - 0.18) | 9.93E-01 |  | 0.04 (-0.32 - 0.40) | 8.43E-01 |
| MHC 24:1 |  | -0.16 (-0.29 - -0.02) | 8.49E-02 |  | -0.18 (-0.60 - 0.25) | 5.13E-01 |  | -0.31 (-0.49 - -0.14) | **1.45E-03** |  | -0.54 (-0.90 - -0.19) | **4.79E-03** |
| DHC 16:0 |  | -0.23 (-0.38 - -0.08) | **1.71E-02** |  | -0.57 (-1.03 - -0.11) | **4.03E-02** |  | -0.34 (-0.52 - -0.16) | **8.97E-04** |  | -0.70 (-1.06 - -0.33) | **5.24E-04** |
| DHC 18:0 |  | -0.17 (-0.31 - -0.02) | 8.81E-02 |  | -0.53 (-0.99 - -0.08) | 5.28E-02 |  | 0.30 (0.13 - 0.47) | **1.51E-03** |  | 0.50 (0.16 - 0.83) | **7.56E-03** |
| DHC 20:0 |  | -0.13 (-0.29 - 0.02) | 2.06E-01 |  | -0.36 (-0.86 - 0.13) | 2.24E-01 |  | 0.20 (0.03 - 0.37) | **3.79E-02** |  | 0.35 (0.01 - 0.69) | 6.64E-02 |
| DHC 22:0 |  | -0.06 (-0.20 - 0.09) | 5.85E-01 |  | -0.33 (-0.79 - 0.12) | 2.20E-01 |  | 0.15 (-0.01 - 0.31) | 1.10E-01 |  | 0.27 (-0.06 - 0.59) | 1.41E-01 |
| DHC 24:0 |  | -0.05 (-0.19 - 0.09) | 6.26E-01 |  | -0.10 (-0.55 - 0.35) | 7.39E-01 |  | 0.18 (0.01 - 0.35) | 6.65E-02 |  | 0.32 (-0.02 - 0.67) | 9.49E-02 |
| DHC 24:1 |  | -0.20 (-0.34 - -0.06) | **3.23E-02** |  | -0.56 (-1.00 - -0.12) | **3.60E-02** |  | -0.28 (-0.45 - -0.10) | **4.74E-03** |  | -0.64 (-0.99 - -0.29) | **7.26E-04** |
| THC 16:0 |  | -0.12 (-0.28 - 0.05) | 2.92E-01 |  | -0.20 (-0.72 - 0.32) | 5.37E-01 |  | -0.24 (-0.41 - -0.06) | **1.53E-02** |  | -0.50 (-0.85 - -0.15) | **8.31E-03** |
| THC 18:0 |  | -0.06 (-0.22 - 0.10) | 5.88E-01 |  | -0.06 (-0.56 - 0.44) | 8.50E-01 |  | 0.00 (-0.17 - 0.16) | 9.95E-01 |  | -0.04 (-0.37 - 0.29) | 8.41E-01 |
| THC 20:0 |  | -0.07 (-0.23 - 0.08) | 5.08E-01 |  | -0.25 (-0.74 - 0.25) | 4.27E-01 |  | 0.02 (-0.16 - 0.19) | 8.97E-01 |  | 0.08 (-0.27 - 0.42) | 7.19E-01 |
| THC 22:0 |  | -0.08 (-0.23 - 0.08) | 4.83E-01 |  | -0.34 (-0.82 - 0.14) | 2.41E-01 |  | 0.03 (-0.15 - 0.21) | 8.30E-01 |  | -0.06 (-0.42 - 0.30) | 7.81E-01 |
| THC 24:0 |  | -0.01 (-0.18 - 0.15) | 9.11E-01 |  | -0.04 (-0.55 - 0.47) | 9.12E-01 |  | -0.09 (-0.27 - 0.08) | 3.79E-01 |  | -0.27 (-0.62 - 0.09) | 1.83E-01 |
| THC 24:1 |  | -0.10 (-0.27 - 0.06) | 3.58E-01 |  | 0.02 (-0.51 - 0.55) | 9.58E-01 |  | -0.25 (-0.43 - -0.08) | **1.07E-02** |  | -0.60 (-0.95 - -0.24) | **1.96E-03** |
| GM3 16:0 |  | -0.16 (-0.32 - 0.01) | 1.70E-01 |  | -0.45 (-0.98 - 0.08) | 1.60E-01 |  | -0.09 (-0.27 - 0.09) | 4.07E-01 |  | -0.09 (-0.45 - 0.27) | 6.66E-01 |
| GM3 18:0 |  | -0.13 (-0.28 - 0.01) | 1.72E-01 |  | -0.48 (-0.93 - -0.02) | 7.96E-02 |  | -0.02 (-0.21 - 0.17) | 8.77E-01 |  | -0.07 (-0.45 - 0.31) | 7.57E-01 |
| GM3 20:0 |  | -0.04 (-0.20 - 0.13) | 7.45E-01 |  | -0.01 (-0.52 - 0.51) | 9.75E-01 |  | 0.30 (0.12 - 0.48) | **2.84E-03** |  | 0.70 (0.34 - 1.06) | **3.21E-04** |
| GM3 22:0 |  | 0.08 (-0.07 - 0.24) | 4.51E-01 |  | 0.22 (-0.27 - 0.70) | 4.85E-01 |  | 0.60 (0.42 - 0.78) | **2.12E-09** |  | 1.29 (0.93 - 1.66) | **4.55E-11** |
| GM3 24:0 |  | -0.02 (-0.19 - 0.14) | 8.55E-01 |  | -0.35 (-0.88 - 0.17) | 2.60E-01 |  | 0.27 (0.08 - 0.45) | **1.18E-02** |  | 0.37 (-0.01 - 0.75) | 7.67E-02 |
| GM3 24:1 |  | -0.01 (-0.17 - 0.14) | 9.09E-01 |  | -0.06 (-0.55 - 0.43) | 8.51E-01 |  | 0.10 (-0.08 - 0.28) | 3.39E-01 |  | 0.22 (-0.14 - 0.57) | 2.97E-01 |
| SM 32:1 |  | -0.02 (-0.15 - 0.11) | 8.12E-01 |  | -0.34 (-0.76 - 0.07) | 1.71E-01 |  | 0.11 (-0.07 - 0.29) | 3.26E-01 |  | 0.38 (0.02 - 0.75) | 5.92E-02 |
| SM 33:1 |  | -0.14 (-0.29 - 0.00) | 1.34E-01 |  | -0.51 (-0.96 - -0.07) | 5.70E-02 |  | -0.01 (-0.18 - 0.17) | 9.56E-01 |  | 0.03 (-0.33 - 0.38) | 9.02E-01 |
| SM 34:1 |  | -0.17 (-0.32 - -0.01) | 1.07E-01 |  | -0.18 (-0.67 - 0.31) | 5.71E-01 |  | -0.11 (-0.30 - 0.08) | 3.43E-01 |  | -0.22 (-0.61 - 0.16) | 3.18E-01 |
| SM 34:2 |  | -0.15 (-0.33 - 0.02) | 1.99E-01 |  | -0.61 (-1.15 - -0.06) | 6.28E-02 |  | -0.16 (-0.38 - 0.05) | 1.89E-01 |  | -0.20 (-0.62 - 0.23) | 4.31E-01 |
| SM 36:1 |  | 0.04 (-0.13 - 0.21) | 7.34E-01 |  | 0.22 (-0.30 - 0.74) | 5.02E-01 |  | 0.15 (-0.04 - 0.34) | 1.74E-01 |  | 0.30 (-0.07 - 0.68) | 1.50E-01 |
| SM 36:2 |  | -0.03 (-0.20 - 0.14) | 7.99E-01 |  | -0.21 (-0.74 - 0.32) | 5.33E-01 |  | -0.09 (-0.28 - 0.11) | 4.47E-01 |  | -0.28 (-0.66 - 0.11) | 2.14E-01 |
| SM 37:2 |  | -0.15 (-0.32 - 0.01) | 1.67E-01 |  | -1.07 (-1.57 - -0.57) | **5.38E-04** |  | -0.27 (-0.46 - -0.08) | **1.21E-02** |  | -0.66 (-1.04 - -0.29) | **1.30E-03** |
| SM 38:1 |  | -0.07 (-0.21 - 0.07) | 4.86E-01 |  | -0.36 (-0.81 - 0.10) | 1.88E-01 |  | -0.09 (-0.28 - 0.09) | 4.03E-01 |  | -0.11 (-0.49 - 0.26) | 6.19E-01 |
| SM 38:2 |  | 0.07 (-0.10 - 0.23) | 5.85E-01 |  | 0.11 (-0.42 - 0.63) | 7.58E-01 |  | -0.57 (-0.77 - -0.38) | **6.30E-08** |  | -1.23 (-1.62 - -0.84) | **3.19E-09** |
| SM 39:1 |  | -0.17 (-0.32 - -0.03) | 7.70E-02 |  | -1.03 (-1.48 - -0.59) | **2.26E-04** |  | 0.06 (-0.13 - 0.26) | 6.14E-01 |  | 0.12 (-0.27 - 0.51) | 6.17E-01 |
| SM 41:1 |  | -0.08 (-0.25 - 0.10) | 5.29E-01 |  | -0.29 (-0.82 - 0.24) | 3.83E-01 |  | 0.15 (-0.03 - 0.33) | 1.63E-01 |  | 0.39 (0.03 - 0.74) | 5.13E-02 |
| SM 42:1 |  | 0.03 (-0.12 - 0.18) | 7.70E-01 |  | 0.05 (-0.42 - 0.52) | 8.60E-01 |  | -0.01 (-0.19 - 0.17) | 9.44E-01 |  | 0.10 (-0.27 - 0.46) | 6.63E-01 |
| PC 28:0 |  | 0.02 (-0.05 - 0.09) | 7.06E-01 |  | -0.09 (-0.32 - 0.13) | 5.25E-01 |  | 0.01 (-0.09 - 0.11) | 8.91E-01 |  | 0.12 (-0.08 - 0.31) | 2.89E-01 |
| PC 29:0 |  | -0.17 (-0.32 - -0.02) | 8.57E-02 |  | -0.61 (-1.07 - -0.15) | **2.84E-02** |  | -0.13 (-0.31 - 0.05) | 2.08E-01 |  | 0.01 (-0.35 - 0.36) | 9.82E-01 |
| PC 31:0 |  | -0.07 (-0.21 - 0.07) | 4.99E-01 |  | -0.44 (-0.88 - -0.01) | 9.12E-02 |  | 0.11 (-0.06 - 0.28) | 2.76E-01 |  | 0.50 (0.16 - 0.85) | **7.89E-03** |
| PC 31:1 |  | -0.12 (-0.27 - 0.03) | 2.18E-01 |  | -0.66 (-1.13 - -0.20) | **1.92E-02** |  | -0.03 (-0.20 - 0.14) | 8.21E-01 |  | -0.06 (-0.41 - 0.28) | 7.60E-01 |
| PC 32:0 |  | 0.01 (-0.13 - 0.15) | 9.11E-01 |  | -0.08 (-0.52 - 0.35) | 7.69E-01 |  | 0.22 (0.04 - 0.40) | **3.46E-02** |  | 0.64 (0.28 - 1.00) | **1.09E-03** |
| PC 32:1 |  | 0.07 (-0.08 - 0.22) | 4.86E-01 |  | 0.43 (-0.04 - 0.89) | 1.32E-01 |  | 0.16 (-0.02 - 0.34) | 1.28E-01 |  | 0.69 (0.33 - 1.05) | **4.06E-04** |
| PC 32:2 |  | -0.05 (-0.20 - 0.10) | 6.40E-01 |  | -0.40 (-0.88 - 0.09) | 1.73E-01 |  | 0.16 (-0.04 - 0.35) | 1.81E-01 |  | 0.60 (0.20 - 0.99) | **5.61E-03** |
| PC 33:0 |  | -0.14 (-0.28 - 0.01) | 1.60E-01 |  | -0.73 (-1.17 - -0.28) | **7.66E-03** |  | 0.06 (-0.12 - 0.23) | 6.06E-01 |  | 0.19 (-0.16 - 0.54) | 3.59E-01 |
| PC 33:1 |  | -0.15 (-0.29 - 0.00) | 1.42E-01 |  | -0.42 (-0.89 - 0.04) | 1.32E-01 |  | -0.05 (-0.23 - 0.14) | 6.92E-01 |  | 0.02 (-0.35 - 0.38) | 9.40E-01 |
| PC 33:2 |  | -0.15 (-0.32 - 0.01) | 1.67E-01 |  | -0.95 (-1.46 - -0.44) | **1.92E-03** |  | 0.16 (-0.02 - 0.33) | 1.29E-01 |  | 0.49 (0.14 - 0.84) | **1.09E-02** |
| PC 34:0 |  | -0.03 (-0.17 - 0.10) | 7.25E-01 |  | -0.55 (-0.97 - -0.12) | **3.35E-02** |  | 0.15 (-0.04 - 0.33) | 1.74E-01 |  | 0.43 (0.06 - 0.79) | **3.58E-02** |
| PC 34:1 |  | 0.01 (-0.13 - 0.14) | 9.46E-01 |  | -0.01 (-0.43 - 0.42) | 9.75E-01 |  | 0.19 (0.00 - 0.39) | 8.43E-02 |  | 0.62 (0.24 - 1.01) | **3.17E-03** |
| PC 34:2 |  | 0.03 (-0.12 - 0.17) | 7.99E-01 |  | -0.61 (-1.06 - -0.16) | **2.64E-02** |  | 0.29 (0.11 - 0.47) | **3.82E-03** |  | 0.79 (0.44 - 1.15) | **3.76E-05** |
| PC 34:3 |  | -0.03 (-0.20 - 0.13) | 7.82E-01 |  | -0.26 (-0.77 - 0.25) | 4.18E-01 |  | 0.15 (-0.03 - 0.34) | 1.65E-01 |  | 0.58 (0.21 - 0.95) | **4.06E-03** |
| PC 34:5 |  | 0.04 (-0.07 - 0.16) | 5.97E-01 |  | -0.09 (-0.46 - 0.28) | 7.14E-01 |  | 0.14 (-0.01 - 0.30) | 1.20E-01 |  | 0.53 (0.21 - 0.84) | **2.19E-03** |
| PC 35:0 |  | -0.07 (-0.17 - 0.02) | 2.42E-01 |  | -0.19 (-0.49 - 0.10) | 2.72E-01 |  | -0.05 (-0.23 - 0.14) | 6.85E-01 |  | -0.22 (-0.59 - 0.15) | 3.00E-01 |
| PC 35:1 |  | -0.15 (-0.31 - 0.00) | 1.32E-01 |  | -0.76 (-1.23 - -0.29) | **8.03E-03** |  | 0.17 (-0.01 - 0.35) | 1.13E-01 |  | 0.24 (-0.13 - 0.60) | 2.62E-01 |
| PC 35:2 |  | -0.18 (-0.33 - -0.03) | 7.84E-02 |  | -1.14 (-1.60 - -0.67) | **1.32E-04** |  | 0.17 (-0.01 - 0.35) | 1.14E-01 |  | 0.15 (-0.22 - 0.52) | 5.05E-01 |
| PC 35:3 |  | -0.18 (-0.33 - -0.02) | 9.31E-02 |  | -0.78 (-1.27 - -0.29) | **8.25E-03** |  | -0.02 (-0.19 - 0.16) | 8.89E-01 |  | 0.07 (-0.28 - 0.41) | 7.57E-01 |
| PC 35:4 |  | -0.10 (-0.22 - 0.02) | 2.21E-01 |  | -0.33 (-0.71 - 0.05) | 1.44E-01 |  | 0.17 (0.00 - 0.34) | 9.48E-02 |  | 0.51 (0.17 - 0.85) | **5.93E-03** |
| PC 36:1 |  | -0.05 (-0.21 - 0.10) | 6.31E-01 |  | -0.65 (-1.13 - -0.17) | **2.64E-02** |  | -0.02 (-0.21 - 0.16) | 8.63E-01 |  | 0.13 (-0.24 - 0.51) | 5.64E-01 |
| PC 36:2 |  | 0.04 (-0.11 - 0.20) | 7.06E-01 |  | -0.52 (-1.00 - -0.03) | 7.50E-02 |  | 0.03 (-0.14 - 0.21) | 7.75E-01 |  | 0.26 (-0.10 - 0.62) | 1.99E-01 |
| PC 36:3 |  | -0.05 (-0.20 - 0.10) | 6.17E-01 |  | -0.42 (-0.89 - 0.05) | 1.37E-01 |  | 0.04 (-0.14 - 0.21) | 7.45E-01 |  | 0.27 (-0.08 - 0.62) | 1.73E-01 |
| PC 36:4b |  | 0.05 (-0.10 - 0.21) | 6.18E-01 |  | -0.06 (-0.55 - 0.43) | 8.50E-01 |  | 0.26 (0.08 - 0.44) | **8.44E-03** |  | 0.68 (0.33 - 1.03) | **3.50E-04** |
| PC 36:5 |  | 0.04 (-0.08 - 0.16) | 6.26E-01 |  | 0.05 (-0.32 - 0.42) | 8.50E-01 |  | 0.24 (0.08 - 0.41) | **9.50E-03** |  | 0.64 (0.31 - 0.98) | **4.22E-04** |
| PC 37:4 |  | -0.15 (-0.30 - 0.00) | 1.38E-01 |  | -0.89 (-1.36 - -0.43) | **1.68E-03** |  | 0.10 (-0.07 - 0.27) | 3.22E-01 |  | 0.08 (-0.26 - 0.42) | 7.08E-01 |
| PC 37:5 |  | -0.09 (-0.22 - 0.05) | 3.32E-01 |  | -0.33 (-0.75 - 0.08) | 1.83E-01 |  | 0.08 (-0.09 - 0.25) | 4.25E-01 |  | 0.05 (-0.29 - 0.39) | 8.10E-01 |
| PC 37:6 |  | -0.04 (-0.16 - 0.09) | 7.06E-01 |  | -0.21 (-0.60 - 0.19) | 4.10E-01 |  | 0.03 (-0.14 - 0.20) | 7.88E-01 |  | 0.18 (-0.15 - 0.52) | 3.42E-01 |
| PC 38:2 |  | -0.02 (-0.17 - 0.13) | 8.68E-01 |  | -0.43 (-0.89 - 0.03) | 1.23E-01 |  | -0.34 (-0.52 - -0.16) | **6.00E-04** |  | -0.53 (-0.88 - -0.17) | **6.98E-03** |
| PC 38:3 |  | -0.05 (-0.19 - 0.09) | 5.88E-01 |  | -0.49 (-0.93 - -0.05) | 6.19E-02 |  | -0.19 (-0.38 - 0.01) | 1.03E-01 |  | 0.12 (-0.26 - 0.51) | 6.09E-01 |
| PC 38:4 |  | 0.11 (-0.05 - 0.26) | 3.02E-01 |  | -0.06 (-0.54 - 0.43) | 8.60E-01 |  | 0.04 (-0.14 - 0.21) | 7.44E-01 |  | 0.19 (-0.16 - 0.54) | 3.63E-01 |
| PC 38:5 |  | -0.03 (-0.16 - 0.10) | 7.45E-01 |  | -0.26 (-0.67 - 0.16) | 3.12E-01 |  | 0.00 (-0.18 - 0.19) | 9.78E-01 |  | 0.00 (-0.36 - 0.36) | 1.00E+00 |
| PC 38:6a |  | -0.07 (-0.20 - 0.07) | 4.74E-01 |  | -0.56 (-0.98 - -0.14) | **2.85E-02** |  | 0.15 (-0.03 - 0.34) | 1.52E-01 |  | 0.37 (0.01 - 0.74) | 6.64E-02 |
| PC 38:6b |  | 0.07 (-0.07 - 0.22) | 4.74E-01 |  | 0.26 (-0.20 - 0.71) | 3.63E-01 |  | 0.08 (-0.09 - 0.25) | 4.25E-01 |  | 0.27 (-0.06 - 0.61) | 1.50E-01 |
| PC 40:5 |  | 0.05 (-0.10 - 0.20) | 6.61E-01 |  | 0.20 (-0.27 - 0.66) | 5.07E-01 |  | -0.12 (-0.31 - 0.07) | 2.84E-01 |  | 0.07 (-0.30 - 0.45) | 7.57E-01 |
| PC 40:6 |  | 0.11 (-0.04 - 0.25) | 2.63E-01 |  | 0.45 (0.00 - 0.90) | 9.83E-02 |  | -0.10 (-0.28 - 0.08) | 3.42E-01 |  | 0.07 (-0.28 - 0.43) | 7.35E-01 |
| PC 40:7 |  | -0.08 (-0.24 - 0.07) | 4.51E-01 |  | -0.16 (-0.65 - 0.34) | 6.19E-01 |  | -0.16 (-0.34 - 0.02) | 1.28E-01 |  | -0.46 (-0.82 - -0.09) | **2.24E-02** |
| PC(O-30:0) |  | -0.12 (-0.26 - 0.03) | 2.23E-01 |  | -0.62 (-1.07 - -0.17) | **2.37E-02** |  | -0.36 (-0.53 - -0.20) | **6.71E-05** |  | -0.55 (-0.89 - -0.22) | **2.37E-03** |
| PC(O-32:0) |  | -0.11 (-0.25 - 0.03) | 2.42E-01 |  | -0.18 (-0.63 - 0.26) | 5.18E-01 |  | -0.40 (-0.58 - -0.22) | **4.34E-05** |  | -0.67 (-1.03 - -0.32) | **5.43E-04** |
| PC(O-32:1) |  | -0.05 (-0.20 - 0.10) | 6.17E-01 |  | -0.03 (-0.51 - 0.44) | 9.17E-01 |  | -0.59 (-0.77 - -0.41) | **1.55E-09** |  | -1.32 (-1.68 - -0.97) | **5.24E-12** |
| PC(O-34:0) |  | -0.13 (-0.27 - 0.02) | 1.88E-01 |  | -0.49 (-0.94 - -0.04) | 6.97E-02 |  | 0.03 (-0.16 - 0.21) | 8.32E-01 |  | 0.12 (-0.24 - 0.49) | 6.00E-01 |
| PC(O-34:1) |  | -0.10 (-0.26 - 0.07) | 4.01E-01 |  | -0.36 (-0.88 - 0.16) | 2.49E-01 |  | -0.36 (-0.54 - -0.19) | **1.70E-04** |  | -0.83 (-1.18 - -0.48) | **9.57E-06** |
| PC(O-34:2) |  | -0.07 (-0.23 - 0.09) | 5.36E-01 |  | -0.61 (-1.11 - -0.11) | **4.15E-02** |  | -0.29 (-0.46 - -0.13) | **1.41E-03** |  | -0.74 (-1.07 - -0.41) | **3.07E-05** |
| PC(O-36:0) |  | -0.09 (-0.24 - 0.06) | 3.53E-01 |  | -0.43 (-0.89 - 0.03) | 1.26E-01 |  | -0.01 (-0.16 - 0.14) | 8.97E-01 |  | 0.09 (-0.21 - 0.40) | 6.13E-01 |
| PC(O-36:1) |  | -0.15 (-0.29 - 0.00) | 1.32E-01 |  | -0.41 (-0.87 - 0.04) | 1.35E-01 |  | -0.04 (-0.20 - 0.13) | 7.16E-01 |  | -0.20 (-0.54 - 0.13) | 2.87E-01 |
| PC(O-36:2) |  | -0.11 (-0.26 - 0.04) | 2.62E-01 |  | -0.67 (-1.14 - -0.21) | **1.71E-02** |  | -0.13 (-0.31 - 0.04) | 2.05E-01 |  | -0.50 (-0.85 - -0.15) | **9.56E-03** |
| PC(O-36:3) |  | -0.06 (-0.20 - 0.08) | 5.36E-01 |  | -0.31 (-0.73 - 0.12) | 2.35E-01 |  | -0.23 (-0.41 - -0.05) | **2.25E-02** |  | -0.55 (-0.90 - -0.19) | **4.95E-03** |
| PC(O-36:4) |  | -0.05 (-0.20 - 0.09) | 5.85E-01 |  | -0.39 (-0.83 - 0.05) | 1.40E-01 |  | 0.08 (-0.10 - 0.26) | 4.73E-01 |  | -0.12 (-0.48 - 0.24) | 6.09E-01 |
| PC(O-36:5) |  | 0.01 (-0.11 - 0.13) | 8.94E-01 |  | 0.09 (-0.29 - 0.47) | 7.14E-01 |  | 0.09 (-0.09 - 0.26) | 4.26E-01 |  | -0.09 (-0.45 - 0.27) | 6.66E-01 |
| PC(O-38:4) |  | -0.12 (-0.26 - 0.02) | 2.13E-01 |  | -0.47 (-0.91 - -0.03) | 7.52E-02 |  | 0.27 (0.10 - 0.44) | **5.38E-03** |  | 0.32 (-0.03 - 0.66) | 9.93E-02 |
| PC(O-38:5) |  | -0.04 (-0.19 - 0.11) | 7.06E-01 |  | -0.28 (-0.75 - 0.18) | 3.22E-01 |  | 0.04 (-0.14 - 0.22) | 7.44E-01 |  | -0.23 (-0.59 - 0.14) | 2.85E-01 |
| PC(O-40:7) |  | 0.07 (-0.08 - 0.21) | 5.36E-01 |  | 0.22 (-0.25 - 0.69) | 4.51E-01 |  | 0.09 (-0.08 - 0.26) | 3.81E-01 |  | 0.02 (-0.32 - 0.36) | 9.21E-01 |
| PC(P-32:0) |  | -0.14 (-0.29 - 0.01) | 1.54E-01 |  | -0.62 (-1.08 - -0.15) | **2.85E-02** |  | -0.37 (-0.54 - -0.20) | **6.50E-05** |  | -1.00 (-1.34 - -0.67) | **2.54E-08** |
| PC(P-34:1) |  | -0.13 (-0.28 - 0.03) | 2.08E-01 |  | -0.58 (-1.05 - -0.10) | **4.33E-02** |  | -0.52 (-0.70 - -0.35) | **2.58E-08** |  | -1.39 (-1.73 - -1.05) | **3.06E-14** |
| PC(P-36:2) |  | -0.18 (-0.32 - -0.04) | **4.97E-02** |  | -0.89 (-1.32 - -0.45) | **7.87E-04** |  | -0.13 (-0.32 - 0.06) | 2.32E-01 |  | -0.59 (-0.96 - -0.21) | **4.07E-03** |
| PC(P-36:5) |  | 0.00 (-0.13 - 0.13) | 9.98E-01 |  | -0.15 (-0.55 - 0.24) | 5.37E-01 |  | 0.04 (-0.09 - 0.17) | 6.31E-01 |  | -0.07 (-0.34 - 0.20) | 6.66E-01 |
| PC(P-38:5) |  | -0.01 (-0.16 - 0.15) | 9.51E-01 |  | -0.17 (-0.65 - 0.31) | 5.79E-01 |  | 0.07 (-0.11 - 0.24) | 5.39E-01 |  | -0.18 (-0.53 - 0.17) | 3.84E-01 |
| LPC 14:0 |  | -0.07 (-0.21 - 0.06) | 4.49E-01 |  | -0.14 (-0.56 - 0.28) | 6.07E-01 |  | 0.09 (-0.08 - 0.25) | 3.79E-01 |  | 0.30 (-0.03 - 0.63) | 1.03E-01 |
| LPC 15:0 |  | -0.26 (-0.41 - -0.12) | **6.20E-03** |  | -0.75 (-1.21 - -0.29) | **7.37E-03** |  | -0.08 (-0.25 - 0.10) | 4.60E-01 |  | -0.29 (-0.64 - 0.05) | 1.31E-01 |
| LPC 16:0 |  | -0.12 (-0.25 - 0.01) | 1.67E-01 |  | -0.06 (-0.47 - 0.35) | 8.42E-01 |  | 0.07 (-0.09 - 0.23) | 4.70E-01 |  | 0.21 (-0.11 - 0.54) | 2.57E-01 |
| LPC 16:1 |  | -0.11 (-0.24 - 0.02) | 2.08E-01 |  | 0.08 (-0.33 - 0.49) | 7.69E-01 |  | -0.08 (-0.23 - 0.06) | 3.26E-01 |  | -0.24 (-0.53 - 0.04) | 1.32E-01 |
| LPC 18:0 |  | -0.12 (-0.26 - 0.02) | 1.99E-01 |  | -0.12 (-0.56 - 0.33) | 6.92E-01 |  | -0.16 (-0.34 - 0.03) | 1.64E-01 |  | -0.47 (-0.85 - -0.09) | **2.48E-02** |
| LPC 18:1 |  | -0.16 (-0.31 - 0.00) | 1.30E-01 |  | -0.62 (-1.10 - -0.14) | **3.08E-02** |  | -0.14 (-0.33 - 0.06) | 2.28E-01 |  | -0.63 (-1.02 - -0.25) | **2.59E-03** |
| LPC 18:2 |  | -0.09 (-0.26 - 0.07) | 4.10E-01 |  | -1.11 (-1.61 - -0.60) | **4.52E-04** |  | 0.00 (-0.18 - 0.18) | 9.94E-01 |  | -0.41 (-0.77 - -0.04) | **4.28E-02** |
| LPC 20:0 |  | -0.10 (-0.25 - 0.05) | 3.36E-01 |  | -0.52 (-0.99 - -0.05) | 6.49E-02 |  | -0.26 (-0.43 - -0.09) | **7.59E-03** |  | -0.65 (-0.99 - -0.30) | **5.79E-04** |
| LPC 20:1 |  | -0.16 (-0.31 - -0.02) | 9.95E-02 |  | -0.56 (-1.03 - -0.10) | **4.43E-02** |  | -0.19 (-0.36 - -0.02) | **4.70E-02** |  | -0.72 (-1.06 - -0.39) | **6.26E-05** |
| LPC 20:2 |  | -0.20 (-0.36 - -0.04) | 5.77E-02 |  | -0.48 (-0.97 - 0.02) | 1.08E-01 |  | -0.16 (-0.32 - 0.00) | 7.79E-02 |  | -0.45 (-0.77 - -0.14) | **8.20E-03** |
| LPC 20:3 |  | -0.07 (-0.21 - 0.08) | 5.04E-01 |  | -0.25 (-0.69 - 0.19) | 3.65E-01 |  | 0.02 (-0.16 - 0.20) | 8.75E-01 |  | 0.05 (-0.31 - 0.40) | 8.27E-01 |
| LPC 20:4 |  | -0.08 (-0.23 - 0.06) | 4.15E-01 |  | -0.41 (-0.86 - 0.04) | 1.31E-01 |  | 0.19 (0.00 - 0.38) | 8.43E-02 |  | 0.12 (-0.26 - 0.49) | 6.17E-01 |
| LPC 20:5 |  | 0.01 (-0.10 - 0.12) | 9.11E-01 |  | -0.09 (-0.42 - 0.25) | 6.92E-01 |  | 0.11 (-0.04 - 0.26) | 2.27E-01 |  | 0.09 (-0.21 - 0.39) | 6.38E-01 |
| LPC 22:0 |  | -0.08 (-0.21 - 0.05) | 3.77E-01 |  | -0.70 (-1.09 - -0.30) | **3.92E-03** |  | -0.09 (-0.26 - 0.08) | 3.76E-01 |  | -0.37 (-0.71 - -0.03) | 5.01E-02 |
| LPC 22:6 |  | 0.03 (-0.11 - 0.16) | 7.78E-01 |  | 0.16 (-0.26 - 0.59) | 5.46E-01 |  | 0.13 (-0.03 - 0.30) | 1.76E-01 |  | 0.28 (-0.05 - 0.62) | 1.30E-01 |
| LPC 24:0 |  | -0.02 (-0.15 - 0.11) | 7.99E-01 |  | -0.49 (-0.89 - -0.09) | **4.34E-02** |  | -0.20 (-0.39 - -0.02) | 5.37E-02 |  | -0.48 (-0.84 - -0.11) | **1.85E-02** |
| LPC(O-22:0) |  | -0.24 (-0.38 - -0.10) | **8.43E-03** |  | -0.90 (-1.34 - -0.47) | **6.37E-04** |  | -0.23 (-0.42 - -0.05) | **2.35E-02** |  | -0.63 (-0.99 - -0.26) | **1.57E-03** |
| LPC(O-24:1) |  | -0.27 (-0.41 - -0.12) | **4.92E-03** |  | -0.87 (-1.32 - -0.42) | **1.68E-03** |  | -0.36 (-0.52 - -0.19) | **1.05E-04** |  | -0.67 (-1.00 - -0.34) | **2.03E-04** |
| LPC(O-24:2) |  | -0.27 (-0.42 - -0.11) | **9.52E-03** |  | -0.81 (-1.31 - -0.32) | **6.99E-03** |  | -0.50 (-0.68 - -0.32) | **2.57E-07** |  | -1.12 (-1.47 - -0.76) | **4.17E-09** |
| PE 32:1 |  | 0.07 (-0.04 - 0.18) | 3.34E-01 |  | 0.44 (0.11 - 0.78) | **2.93E-02** |  | 0.15 (0.03 - 0.27) | **2.76E-02** |  | 0.64 (0.40 - 0.87) | **5.67E-07** |
| PE 34:1 |  | 0.18 (0.04 - 0.31) | **4.67E-02** |  | 0.64 (0.22 - 1.05) | **1.11E-02** |  | 0.44 (0.28 - 0.59) | **2.66E-07** |  | 1.24 (0.93 - 1.54) | **7.59E-14** |
| PE 34:2 |  | 0.09 (-0.05 - 0.24) | 3.18E-01 |  | 0.50 (0.06 - 0.94) | 6.06E-02 |  | 0.44 (0.27 - 0.61) | **2.66E-06** |  | 1.33 (1.00 - 1.67) | **1.44E-13** |
| PE 36:1 |  | 0.32 (0.18 - 0.46) | **6.98E-04** |  | 0.76 (0.32 - 1.21) | **4.61E-03** |  | 0.60 (0.43 - 0.77) | **1.34E-10** |  | 1.51 (1.18 - 1.85) | **5.85E-17** |
| PE 36:2 |  | 0.28 (0.13 - 0.43) | **4.67E-03** |  | 0.70 (0.23 - 1.16) | **1.39E-02** |  | 0.56 (0.38 - 0.73) | **3.96E-09** |  | 1.50 (1.16 - 1.84) | **3.65E-16** |
| PE 36:3 |  | 0.15 (0.01 - 0.30) | 1.26E-01 |  | 0.38 (-0.08 - 0.84) | 1.68E-01 |  | 0.42 (0.26 - 0.58) | **1.39E-06** |  | 1.09 (0.77 - 1.41) | **1.63E-10** |
| PE 36:4 |  | 0.13 (-0.01 - 0.27) | 1.54E-01 |  | 0.51 (0.08 - 0.94) | **4.81E-02** |  | 0.52 (0.35 - 0.68) | **1.20E-08** |  | 1.40 (1.08 - 1.73) | **2.21E-15** |
| PE 36:5 |  | 0.10 (-0.04 - 0.25) | 2.93E-01 |  | 0.47 (0.01 - 0.93) | 9.14E-02 |  | 0.35 (0.19 - 0.51) | **7.55E-05** |  | 1.02 (0.71 - 1.34) | **1.56E-09** |
| PE 38:3 |  | 0.19 (0.04 - 0.34) | 6.30E-02 |  | 0.74 (0.26 - 1.21) | **1.07E-02** |  | 0.48 (0.29 - 0.66) | **3.47E-06** |  | 1.49 (1.12 - 1.86) | **5.31E-14** |
| PE 38:4 |  | 0.22 (0.06 - 0.39) | **3.58E-02** |  | 0.64 (0.13 - 1.14) | **3.71E-02** |  | 0.60 (0.42 - 0.78) | **1.16E-09** |  | 1.61 (1.25 - 1.96) | **9.35E-17** |
| PE 38:5 |  | 0.11 (-0.03 - 0.25) | 2.18E-01 |  | 0.39 (-0.05 - 0.83) | 1.40E-01 |  | 0.56 (0.40 - 0.73) | **6.52E-10** |  | 1.40 (1.07 - 1.73) | **3.31E-15** |
| PE 38:6 |  | 0.21 (0.07 - 0.36) | **2.76E-02** |  | 0.74 (0.29 - 1.19) | **7.49E-03** |  | 0.51 (0.36 - 0.67) | **2.09E-09** |  | 1.35 (1.04 - 1.66) | **6.85E-16** |
| PE 40:6 |  | 0.27 (0.13 - 0.41) | **4.51E-03** |  | 0.92 (0.49 - 1.35) | **5.82E-04** |  | 0.55 (0.38 - 0.71) | **2.43E-09** |  | 1.47 (1.14 - 1.80) | **1.89E-16** |
| PE 40:7 |  | 0.12 (-0.02 - 0.26) | 1.99E-01 |  | 0.53 (0.10 - 0.97) | **4.16E-02** |  | 0.42 (0.28 - 0.56) | **4.79E-08** |  | 0.98 (0.70 - 1.26) | **7.37E-11** |
| PI 32:0 |  | 0.02 (-0.08 - 0.13) | 7.45E-01 |  | 0.28 (-0.06 - 0.62) | 1.73E-01 |  | 0.21 (0.08 - 0.34) | **5.26E-03** |  | 0.79 (0.53 - 1.06) | **2.08E-08** |
| PI 32:1 |  | 0.05 (-0.06 - 0.16) | 5.36E-01 |  | 0.51 (0.16 - 0.86) | **1.68E-02** |  | 0.05 (-0.08 - 0.19) | 5.33E-01 |  | 0.46 (0.19 - 0.73) | **1.60E-03** |
| PI 34:0 |  | 0.03 (-0.10 - 0.16) | 7.40E-01 |  | 0.38 (-0.01 - 0.78) | 1.03E-01 |  | 0.21 (0.09 - 0.34) | **1.68E-03** |  | 0.64 (0.40 - 0.89) | **1.01E-06** |
| PI 34:1 |  | 0.05 (-0.07 - 0.18) | 5.36E-01 |  | 0.45 (0.06 - 0.84) | 5.30E-02 |  | 0.12 (-0.04 - 0.27) | 1.98E-01 |  | 0.56 (0.25 - 0.86) | **7.83E-04** |
| PI 36:0 |  | 0.01 (-0.13 - 0.15) | 9.36E-01 |  | -0.27 (-0.70 - 0.17) | 3.12E-01 |  | 0.17 (0.01 - 0.33) | 7.49E-02 |  | 0.65 (0.32 - 0.98) | **2.71E-04** |
| PI 36:1 |  | 0.06 (-0.08 - 0.20) | 5.53E-01 |  | -0.18 (-0.62 - 0.25) | 5.11E-01 |  | 0.14 (-0.01 - 0.29) | 1.20E-01 |  | 0.37 (0.06 - 0.68) | **2.91E-02** |
| PI 36:2 |  | 0.01 (-0.16 - 0.18) | 9.11E-01 |  | 0.11 (-0.43 - 0.64) | 7.58E-01 |  | 0.13 (-0.04 - 0.30) | 2.02E-01 |  | 0.44 (0.10 - 0.78) | **1.99E-02** |
| PI 36:3 |  | -0.05 (-0.21 - 0.12) | 6.91E-01 |  | -0.04 (-0.55 - 0.48) | 9.18E-01 |  | -0.16 (-0.33 - 0.00) | 8.43E-02 |  | -0.17 (-0.50 - 0.16) | 3.71E-01 |
| PI 36:4 |  | 0.04 (-0.11 - 0.19) | 7.13E-01 |  | 0.49 (0.04 - 0.95) | 7.32E-02 |  | -0.03 (-0.20 - 0.15) | 8.31E-01 |  | 0.36 (0.00 - 0.72) | 6.80E-02 |
| PI 38:2 |  | -0.01 (-0.16 - 0.14) | 9.11E-01 |  | 0.17 (-0.29 - 0.64) | 5.63E-01 |  | 0.15 (0.00 - 0.30) | 8.37E-02 |  | 0.47 (0.17 - 0.76) | **4.30E-03** |
| PI 38:3 |  | -0.04 (-0.22 - 0.13) | 7.25E-01 |  | 0.07 (-0.48 - 0.62) | 8.51E-01 |  | 0.15 (-0.03 - 0.33) | 1.47E-01 |  | 0.62 (0.27 - 0.97) | **1.08E-03** |
| PI 38:4 |  | 0.03 (-0.13 - 0.19) | 7.82E-01 |  | 0.11 (-0.40 - 0.62) | 7.43E-01 |  | 0.30 (0.11 - 0.49) | **5.03E-03** |  | 0.75 (0.37 - 1.13) | **2.71E-04** |
| PI 38:5 |  | -0.11 (-0.27 - 0.06) | 3.36E-01 |  | -0.02 (-0.55 - 0.50) | 9.54E-01 |  | -0.12 (-0.28 - 0.04) | 2.13E-01 |  | -0.21 (-0.54 - 0.11) | 2.56E-01 |
| PI 38:6 |  | 0.08 (-0.06 - 0.22) | 3.84E-01 |  | 0.60 (0.17 - 1.03) | **2.10E-02** |  | -0.10 (-0.27 - 0.07) | 3.29E-01 |  | 0.14 (-0.20 - 0.48) | 4.90E-01 |
| PI 40:4 |  | 0.10 (-0.05 - 0.26) | 3.31E-01 |  | 0.51 (0.03 - 1.00) | 7.83E-02 |  | 0.28 (0.11 - 0.46) | **3.67E-03** |  | 0.83 (0.48 - 1.18) | **1.01E-05** |
| PI 40:5 |  | 0.12 (-0.04 - 0.27) | 2.49E-01 |  | 0.44 (-0.05 - 0.92) | 1.35E-01 |  | 0.21 (0.03 - 0.38) | **3.82E-02** |  | 0.61 (0.26 - 0.96) | **1.37E-03** |
| PI 40:6 |  | 0.18 (0.04 - 0.33) | 6.43E-02 |  | 0.77 (0.31 - 1.22) | **6.06E-03** |  | 0.09 (-0.07 - 0.26) | 3.39E-01 |  | 0.46 (0.13 - 0.79) | **1.06E-02** |
| PS 36:1 |  | 0.02 (-0.08 - 0.12) | 7.45E-01 |  | 0.04 (-0.28 - 0.36) | 8.50E-01 |  | 0.00 (-0.15 - 0.14) | 9.76E-01 |  | 0.09 (-0.21 - 0.38) | 6.20E-01 |
| PS 36:2 |  | 0.02 (-0.10 - 0.14) | 7.99E-01 |  | -0.02 (-0.40 - 0.37) | 9.54E-01 |  | -0.03 (-0.19 - 0.13) | 7.66E-01 |  | 0.04 (-0.28 - 0.36) | 8.41E-01 |
| PS 38:3 |  | -0.01 (-0.14 - 0.12) | 9.28E-01 |  | 0.23 (-0.19 - 0.64) | 3.83E-01 |  | 0.03 (-0.11 - 0.17) | 7.31E-01 |  | 0.11 (-0.17 - 0.38) | 5.09E-01 |
| PS 38:4 |  | 0.03 (-0.08 - 0.14) | 7.06E-01 |  | 0.24 (-0.10 - 0.58) | 2.41E-01 |  | 0.02 (-0.13 - 0.16) | 8.64E-01 |  | 0.09 (-0.20 - 0.37) | 6.20E-01 |
| PS 38:5 |  | -0.07 (-0.20 - 0.06) | 4.21E-01 |  | 0.22 (-0.19 - 0.62) | 3.84E-01 |  | -0.01 (-0.16 - 0.15) | 9.43E-01 |  | 0.03 (-0.27 - 0.34) | 8.47E-01 |
| PS 40:5 |  | 0.09 (-0.03 - 0.22) | 2.49E-01 |  | 0.44 (0.05 - 0.83) | 5.96E-02 |  | -0.02 (-0.15 - 0.11) | 8.37E-01 |  | 0.02 (-0.25 - 0.29) | 9.02E-01 |
| PS 40:6 |  | 0.09 (-0.03 - 0.20) | 2.49E-01 |  | 0.50 (0.14 - 0.86) | **2.25E-02** |  | 0.06 (-0.09 - 0.21) | 5.30E-01 |  | 0.24 (-0.06 - 0.54) | 1.55E-01 |
| PG 34:1 |  | 0.06 (-0.08 - 0.20) | 5.36E-01 |  | 0.28 (-0.16 - 0.71) | 2.98E-01 |  | 0.30 (0.14 - 0.47) | **1.00E-03** |  | 0.90 (0.57 - 1.22) | **3.48E-07** |
| PG 34:2 |  | -0.01 (-0.16 - 0.14) | 9.36E-01 |  | 0.14 (-0.33 - 0.60) | 6.46E-01 |  | 0.17 (0.01 - 0.32) | 5.93E-02 |  | 0.57 (0.26 - 0.88) | **7.57E-04** |
| PG 36:1 |  | 0.26 (0.12 - 0.39) | **4.51E-03** |  | 0.65 (0.23 - 1.08) | **1.16E-02** |  | 0.43 (0.27 - 0.59) | **5.51E-07** |  | 1.24 (0.93 - 1.55) | **1.24E-13** |
| PG 36:2 |  | 0.23 (0.09 - 0.37) | **1.14E-02** |  | 0.64 (0.21 - 1.08) | **1.39E-02** |  | 0.43 (0.26 - 0.60) | **6.35E-06** |  | 1.16 (0.82 - 1.51) | **2.77E-10** |
| COH |  | -0.05 (-0.20 - 0.09) | **4.97E-02** |  | -0.22 (-0.66 - 0.23) | **1.91E-02** |  | 0.13 (-0.05 - 0.31) | **3.14E-04** |  | 0.32 (-0.04 - 0.68) | **3.37E-08** |
| CE 14:0 |  | 0.17 (0.04 - 0.31) | 9.28E-01 |  | 0.59 (0.17 - 1.00) | 6.63E-01 |  | 0.31 (0.15 - 0.46) | **1.02E-02** |  | 0.91 (0.60 - 1.22) | **7.37E-04** |
| CE 15:0 |  | 0.01 (-0.14 - 0.16) | 5.01E-02 |  | 0.13 (-0.34 - 0.61) | 1.06E-01 |  | 0.23 (0.07 - 0.39) | **2.05E-04** |  | 0.59 (0.27 - 0.91) | **4.82E-07** |
| CE 16:0 |  | 0.18 (0.04 - 0.32) | **4.50E-02** |  | 0.43 (-0.01 - 0.87) | **8.01E-04** |  | 0.35 (0.18 - 0.52) | **3.53E-02** |  | 0.91 (0.58 - 1.25) | **4.57E-05** |
| CE 16:1 |  | 0.18 (0.05 - 0.31) | **2.73E-02** |  | 0.85 (0.43 - 1.26) | **5.82E-04** |  | 0.19 (0.03 - 0.34) | **1.46E-03** |  | 0.68 (0.37 - 0.99) | **9.76E-08** |
| CE 16:2 |  | 0.20 (0.06 - 0.33) | 5.08E-01 |  | 0.88 (0.47 - 1.30) | 1.91E-01 |  | 0.31 (0.13 - 0.48) | 1.74E-01 |  | 0.98 (0.64 - 1.33) | 3.84E-01 |
| CE 17:0 |  | -0.06 (-0.20 - 0.07) | 7.40E-01 |  | -0.34 (-0.77 - 0.09) | 2.70E-01 |  | 0.13 (-0.03 - 0.29) | **1.93E-04** |  | 0.17 (-0.16 - 0.49) | **5.19E-06** |
| CE 17:1 |  | 0.03 (-0.11 - 0.18) | 9.36E-01 |  | 0.30 (-0.15 - 0.75) | 9.43E-01 |  | 0.36 (0.19 - 0.54) | 4.46E-01 |  | 0.87 (0.51 - 1.22) | 1.87E-01 |
| CE 18:0 |  | 0.01 (-0.13 - 0.15) | 1.08E-01 |  | -0.02 (-0.46 - 0.41) | **3.87E-02** |  | 0.08 (-0.10 - 0.26) | **2.65E-02** |  | 0.27 (-0.09 - 0.63) | **3.20E-03** |
| CE 18:1 |  | 0.16 (0.01 - 0.31) | **6.33E-03** |  | 0.58 (0.11 - 1.04) | **1.92E-03** |  | 0.22 (0.05 - 0.40) | **3.16E-10** |  | 0.57 (0.22 - 0.93) | **4.76E-13** |
| CE 18:2 |  | 0.23 (0.10 - 0.37) | **1.73E-02** |  | 0.78 (0.36 - 1.19) | **4.94E-03** |  | 0.52 (0.37 - 0.67) | **1.24E-02** |  | 1.17 (0.87 - 1.47) | **3.48E-07** |
| CE 18:3 |  | 0.22 (0.08 - 0.36) | 6.26E-01 |  | 0.74 (0.31 - 1.18) | 5.89E-01 |  | 0.24 (0.07 - 0.41) | 2.98E-01 |  | 0.91 (0.58 - 1.25) | **4.08E-02** |
| CE 20:1 |  | -0.04 (-0.17 - 0.08) | 3.58E-01 |  | -0.13 (-0.52 - 0.25) | 4.49E-01 |  | -0.10 (-0.27 - 0.06) | 3.53E-01 |  | -0.37 (-0.70 - -0.04) | 8.08E-01 |
| CE 20:2 |  | 0.05 (-0.03 - 0.13) | **1.20E-02** |  | 0.12 (-0.13 - 0.38) | **1.62E-03** |  | -0.09 (-0.26 - 0.08) | 1.76E-01 |  | -0.05 (-0.39 - 0.29) | **6.01E-04** |
| CE 20:3 |  | 0.25 (0.10 - 0.40) | **5.29E-03** |  | 0.91 (0.44 - 1.39) | **1.92E-03** |  | 0.15 (-0.04 - 0.33) | **3.13E-07** |  | 0.69 (0.32 - 1.06) | **3.69E-09** |
| CE 20:4 |  | 0.25 (0.11 - 0.38) | **1.79E-02** |  | 0.79 (0.36 - 1.21) | **1.28E-02** |  | 0.44 (0.28 - 0.60) | **4.61E-04** |  | 1.00 (0.68 - 1.31) | **1.13E-05** |
| CE 20:5 |  | 0.19 (0.07 - 0.32) | 5.77E-02 |  | 0.59 (0.20 - 0.98) | 9.50E-02 |  | 0.28 (0.14 - 0.42) | **1.53E-02** |  | 0.68 (0.39 - 0.97) | **5.58E-05** |
| CE 22:0 |  | 0.14 (0.03 - 0.25) | 8.02E-01 |  | 0.34 (0.00 - 0.68) | 2.16E-01 |  | 0.20 (0.05 - 0.34) | 3.29E-01 |  | 0.62 (0.34 - 0.91) | **3.73E-02** |
| CE 22:1 |  | 0.02 (-0.08 - 0.12) | 8.57E-02 |  | 0.24 (-0.08 - 0.55) | **6.26E-03** |  | 0.08 (-0.06 - 0.22) | 2.11E-01 |  | 0.33 (0.04 - 0.61) | 1.16E-01 |
| CE 22:4 |  | 0.18 (0.02 - 0.33) | **8.76E-03** |  | 0.80 (0.32 - 1.27) | **1.80E-03** |  | 0.14 (-0.05 - 0.33) | **2.71E-03** |  | 0.34 (-0.05 - 0.72) | **4.53E-05** |
| CE 22:5 |  | 0.22 (0.09 - 0.36) | **8.44E-03** |  | 0.78 (0.37 - 1.19) | **1.92E-03** |  | 0.31 (0.12 - 0.49) | **1.24E-05** |  | 0.81 (0.44 - 1.18) | **2.47E-06** |
| CE 22:6 |  | 0.21 (0.09 - 0.34) | **3.46E-03** |  | 0.72 (0.33 - 1.11) | 5.09E-02 |  | 0.39 (0.23 - 0.55) | **1.63E-04** |  | 0.82 (0.49 - 1.14) | **1.04E-08** |
| CE 24:0 |  | 0.19 (0.09 - 0.28) | 1.11E-01 |  | 0.35 (0.05 - 0.64) | **1.27E-02** |  | 0.31 (0.16 - 0.45) | 5.33E-01 |  | 0.89 (0.60 - 1.18) | **3.28E-02** |
| CE 24:1 |  | 0.14 (0.01 - 0.27) | **1.81E-02** |  | 0.62 (0.21 - 1.03) | **3.14E-03** |  | 0.06 (-0.10 - 0.22) | 5.27E-02 |  | 0.37 (0.06 - 0.68) | 1.16E-01 |
| CE 24:4 |  | 0.22 (0.08 - 0.36) | **9.85E-04** |  | 0.79 (0.35 - 1.24) | **3.90E-04** |  | -0.17 (-0.33 - -0.02) | 3.99E-01 |  | -0.28 (-0.59 - 0.04) | 5.46E-02 |
| CE 24:5 |  | 0.23 (0.12 - 0.33) | **1.41E-02** |  | 0.71 (0.39 - 1.03) | **3.53E-03** |  | 0.08 (-0.08 - 0.24) | 3.76E-01 |  | 0.34 (0.02 - 0.67) | **3.74E-02** |
| CE 24:6 |  | 0.18 (0.07 - 0.29) | 5.88E-01 |  | 0.62 (0.27 - 0.97) | 4.40E-01 |  | 0.08 (-0.07 - 0.24) | 2.13E-01 |  | 0.36 (0.05 - 0.67) | 1.12E-01 |
| DG 14:0/16:0 |  | 0.13 (0.02 - 0.24) | 7.96E-02 |  | 0.65 (0.31 - 0.99) | **1.74E-03** |  | 0.25 (0.13 - 0.36) | **1.07E-04** |  | 0.81 (0.58 - 1.04) | **3.42E-11** |
| DG 14:0/18:1 |  | 0.12 (-0.01 - 0.25) | 1.70E-01 |  | 0.68 (0.27 - 1.09) | **6.43E-03** |  | 0.29 (0.15 - 0.43) | **1.41E-04** |  | 0.93 (0.66 - 1.21) | **3.12E-10** |
| DG 14:0/18:2 |  | 0.11 (-0.01 - 0.23) | 1.88E-01 |  | 0.49 (0.12 - 0.87) | **2.85E-02** |  | 0.24 (0.11 - 0.38) | **8.56E-04** |  | 0.81 (0.55 - 1.08) | **6.35E-09** |
| DG 16:0/16:0 |  | 0.14 (0.04 - 0.24) | **3.80E-02** |  | 0.78 (0.47 - 1.09) | **8.31E-05** |  | 0.45 (0.33 - 0.57) | **7.47E-12** |  | 1.26 (1.03 - 1.49) | **1.96E-23** |
| DG 16:0/18:0 |  | 0.20 (0.09 - 0.31) | **5.15E-03** |  | 0.81 (0.48 - 1.15) | **1.32E-04** |  | 0.48 (0.35 - 0.60) | **7.47E-12** |  | 1.22 (0.97 - 1.47) | **1.14E-19** |
| DG 16:0/18:1 |  | 0.15 (0.03 - 0.27) | 7.19E-02 |  | 0.96 (0.59 - 1.33) | **8.31E-05** |  | 0.58 (0.44 - 0.73) | **6.72E-13** |  | 1.52 (1.23 - 1.81) | **2.51E-22** |
| DG 16:0/18:2 |  | 0.15 (0.03 - 0.28) | 7.27E-02 |  | 0.90 (0.52 - 1.29) | **2.26E-04** |  | 0.58 (0.43 - 0.73) | **4.03E-12** |  | 1.52 (1.23 - 1.82) | **1.47E-21** |
| DG 16:0/20:3 |  | 0.11 (-0.01 - 0.24) | 1.68E-01 |  | 0.73 (0.34 - 1.11) | **1.80E-03** |  | 0.55 (0.40 - 0.69) | **1.31E-11** |  | 1.54 (1.26 - 1.83) | **3.23E-23** |
| DG 16:0/20:4 |  | 0.18 (0.06 - 0.29) | **1.96E-02** |  | 0.76 (0.40 - 1.12) | **5.85E-04** |  | 0.54 (0.41 - 0.67) | **1.67E-14** |  | 1.37 (1.12 - 1.62) | **8.74E-24** |
| DG 16:0/22:5 |  | 0.10 (-0.02 - 0.22) | 1.97E-01 |  | 0.77 (0.41 - 1.14) | **5.85E-04** |  | 0.66 (0.52 - 0.80) | **4.62E-17** |  | 1.64 (1.37 - 1.92) | **4.01E-27** |
| DG 16:0/22:6 |  | 0.07 (-0.01 - 0.16) | 2.08E-01 |  | 0.56 (0.29 - 0.82) | **5.85E-04** |  | 0.56 (0.44 - 0.68) | **8.22E-16** |  | 1.37 (1.13 - 1.62) | **1.80E-24** |
| DG 16:1/18:0 |  | 0.19 (0.07 - 0.32) | **1.95E-02** |  | 0.98 (0.60 - 1.37) | **8.31E-05** |  | 0.25 (0.13 - 0.37) | **2.12E-04** |  | 0.83 (0.59 - 1.07) | **2.11E-10** |
| DG 16:1/18:1 |  | 0.13 (-0.02 - 0.27) | 1.88E-01 |  | 1.05 (0.61 - 1.48) | **1.65E-04** |  | 0.30 (0.15 - 0.45) | **3.17E-04** |  | 0.94 (0.64 - 1.23) | **4.45E-09** |
| DG 18:0/18:1 |  | 0.25 (0.13 - 0.37) | **1.88E-03** |  | 0.94 (0.58 - 1.31) | **8.31E-05** |  | 0.45 (0.33 - 0.58) | **7.62E-11** |  | 1.09 (0.84 - 1.34) | **6.63E-16** |
| DG 18:0/18:2 |  | 0.24 (0.12 - 0.37) | **4.51E-03** |  | 0.86 (0.48 - 1.25) | **3.90E-04** |  | 0.50 (0.37 - 0.63) | **4.38E-12** |  | 1.20 (0.94 - 1.46) | **9.45E-18** |
| DG 18:0/20:4 |  | 0.20 (0.06 - 0.34) | **2.57E-02** |  | 0.65 (0.22 - 1.08) | **1.33E-02** |  | 0.62 (0.47 - 0.76) | **1.47E-14** |  | 1.45 (1.17 - 1.73) | **4.64E-21** |
| DG 18:1/18:1 |  | 0.16 (0.04 - 0.29) | 5.01E-02 |  | 0.88 (0.50 - 1.26) | **2.61E-04** |  | 0.50 (0.36 - 0.65) | **2.82E-10** |  | 1.19 (0.90 - 1.47) | **2.39E-14** |
| DG 18:1/18:2 |  | 0.14 (0.01 - 0.27) | 9.93E-02 |  | 0.76 (0.35 - 1.16) | **1.92E-03** |  | 0.53 (0.37 - 0.68) | **3.95E-10** |  | 1.26 (0.96 - 1.57) | **1.79E-14** |
| DG 18:1/18:3 |  | 0.14 (0.02 - 0.26) | 8.81E-02 |  | 0.57 (0.19 - 0.95) | **1.32E-02** |  | 0.33 (0.20 - 0.47) | **8.62E-06** |  | 0.90 (0.63 - 1.17) | **4.98E-10** |
| DG 18:1/20:3 |  | 0.08 (-0.05 - 0.21) | 3.40E-01 |  | 0.39 (-0.01 - 0.80) | 1.04E-01 |  | 0.43 (0.29 - 0.57) | **1.20E-08** |  | 1.10 (0.83 - 1.38) | **8.44E-14** |
| DG 18:1/20:4 |  | 0.19 (0.07 - 0.31) | **1.79E-02** |  | 0.71 (0.33 - 1.09) | **1.92E-03** |  | 0.57 (0.43 - 0.71) | **4.25E-13** |  | 1.35 (1.07 - 1.63) | **3.68E-19** |
| DG 18:2/18:2 |  | 0.11 (0.00 - 0.22) | 1.32E-01 |  | 0.48 (0.15 - 0.82) | **1.61E-02** |  | 0.35 (0.22 - 0.49) | **1.53E-06** |  | 0.87 (0.60 - 1.13) | **1.46E-09** |
| TG 14:0/16:0/18:1 |  | 0.16 (0.03 - 0.29) | 7.26E-02 |  | 0.52 (0.11 - 0.92) | **3.44E-02** |  | 0.28 (0.13 - 0.44) | **6.83E-04** |  | 0.97 (0.67 - 1.27) | **1.37E-09** |
| TG 14:0/16:0/18:2 |  | 0.13 (0.00 - 0.25) | 1.25E-01 |  | 0.56 (0.18 - 0.93) | **1.38E-02** |  | 0.19 (0.05 - 0.33) | **1.73E-02** |  | 0.80 (0.52 - 1.07) | **8.01E-08** |
| TG 14:0/16:1/18:1 |  | 0.11 (-0.02 - 0.24) | 2.08E-01 |  | 0.33 (-0.08 - 0.75) | 1.83E-01 |  | 0.08 (-0.06 - 0.21) | 3.42E-01 |  | 0.39 (0.13 - 0.66) | **6.45E-03** |
| TG 14:0/16:1/18:2 |  | 0.09 (-0.02 - 0.20) | 2.33E-01 |  | 0.24 (-0.12 - 0.60) | 2.68E-01 |  | 0.03 (-0.09 - 0.15) | 7.14E-01 |  | 0.29 (0.04 - 0.54) | **3.51E-02** |
| TG 14:0/17:0/18:1 |  | 0.09 (-0.05 - 0.22) | 3.29E-01 |  | 0.29 (-0.13 - 0.72) | 2.54E-01 |  | 0.33 (0.18 - 0.48) | **5.05E-05** |  | 0.94 (0.65 - 1.24) | **2.13E-09** |
| TG 14:0/18:0/18:1 |  | 0.23 (0.11 - 0.36) | **4.51E-03** |  | 0.53 (0.14 - 0.92) | **2.53E-02** |  | 0.30 (0.16 - 0.44) | **1.11E-04** |  | 0.84 (0.56 - 1.11) | **1.62E-08** |
| TG 14:0/18:2/18:2 |  | 0.11 (-0.02 - 0.23) | 2.03E-01 |  | 0.30 (-0.10 - 0.69) | 2.10E-01 |  | 0.09 (-0.05 - 0.22) | 2.81E-01 |  | 0.43 (0.16 - 0.70) | **3.86E-03** |
| TG 14:1/16:0/18:1 |  | 0.09 (-0.04 - 0.23) | 2.78E-01 |  | 0.39 (-0.02 - 0.80) | 1.18E-01 |  | 0.16 (0.02 - 0.30) | **4.02E-02** |  | 0.65 (0.37 - 0.92) | **1.23E-05** |
| TG 14:1/16:1/18:0 |  | 0.10 (-0.02 - 0.22) | 2.08E-01 |  | 0.68 (0.31 - 1.04) | **1.97E-03** |  | 0.09 (-0.02 - 0.20) | 1.76E-01 |  | 0.50 (0.28 - 0.73) | **3.86E-05** |
| TG 14:1/18:0/18:2 |  | 0.10 (-0.02 - 0.22) | 1.99E-01 |  | 0.25 (-0.13 - 0.63) | 2.78E-01 |  | 0.17 (0.03 - 0.31) | **3.76E-02** |  | 0.55 (0.27 - 0.83) | **3.31E-04** |
| TG 14:1/18:1/18:1 |  | 0.09 (-0.05 - 0.22) | 3.31E-01 |  | 0.38 (-0.04 - 0.80) | 1.35E-01 |  | 0.15 (-0.01 - 0.31) | 1.03E-01 |  | 0.64 (0.32 - 0.96) | **2.45E-04** |
| TG 15:0/16:0/18:1 |  | 0.12 (-0.02 - 0.26) | 1.88E-01 |  | 0.47 (0.05 - 0.90) | 6.55E-02 |  | 0.36 (0.21 - 0.51) | **8.64E-06** |  | 1.06 (0.77 - 1.36) | **1.34E-11** |
| TG 15:0/18:1/18:1 |  | 0.06 (-0.08 - 0.20) | 5.26E-01 |  | 0.17 (-0.26 - 0.61) | 5.27E-01 |  | 0.25 (0.10 - 0.41) | **2.75E-03** |  | 0.67 (0.37 - 0.97) | **4.57E-05** |
| TG 16:0/16:0/16:0 |  | 0.18 (0.09 - 0.27) | **4.51E-03** |  | 0.64 (0.35 - 0.93) | **3.90E-04** |  | 0.31 (0.19 - 0.43) | **1.56E-06** |  | 0.97 (0.74 - 1.20) | **6.85E-15** |
| TG 16:0/16:0/18:0 |  | 0.24 (0.14 - 0.34) | **1.05E-04** |  | 0.59 (0.28 - 0.90) | **1.62E-03** |  | 0.38 (0.26 - 0.50) | **5.00E-09** |  | 1.01 (0.78 - 1.25) | **2.21E-15** |
| TG 16:0/16:0/18:1 |  | 0.21 (0.10 - 0.33) | **4.92E-03** |  | 0.81 (0.46 - 1.17) | **2.61E-04** |  | 0.56 (0.41 - 0.72) | **4.96E-11** |  | 1.60 (1.30 - 1.90) | **2.34E-22** |
| TG 16:0/16:0/18:2 |  | 0.21 (0.09 - 0.33) | **6.85E-03** |  | 0.76 (0.39 - 1.13) | **6.63E-04** |  | 0.59 (0.43 - 0.75) | **1.43E-11** |  | 1.71 (1.40 - 2.01) | **6.77E-24** |
| TG 16:0/16:1/17:0 |  | 0.12 (-0.01 - 0.25) | 1.70E-01 |  | 0.50 (0.10 - 0.91) | **3.76E-02** |  | 0.37 (0.22 - 0.51) | **6.35E-06** |  | 1.09 (0.80 - 1.38) | **3.67E-12** |
| TG 16:0/16:1/18:1 |  | 0.14 (0.00 - 0.28) | 1.48E-01 |  | 0.73 (0.29 - 1.16) | **6.23E-03** |  | 0.23 (0.08 - 0.39) | **8.33E-03** |  | 0.88 (0.57 - 1.20) | **1.35E-07** |
| TG 16:0/17:0/18:0 |  | 0.20 (0.06 - 0.33) | **2.46E-02** |  | 0.66 (0.25 - 1.07) | **8.65E-03** |  | 0.33 (0.22 - 0.44) | **3.25E-08** |  | 0.77 (0.55 - 0.98) | **5.43E-11** |
| TG 16:0/17:0/18:1 |  | 0.19 (0.06 - 0.32) | **2.30E-02** |  | 0.55 (0.14 - 0.95) | **2.57E-02** |  | 0.49 (0.35 - 0.63) | **5.26E-10** |  | 1.21 (0.93 - 1.49) | **3.23E-15** |
| TG 16:0/17:0/18:2 |  | 0.13 (-0.01 - 0.27) | 1.59E-01 |  | 0.59 (0.16 - 1.01) | **2.37E-02** |  | 0.40 (0.24 - 0.56) | **3.87E-06** |  | 1.11 (0.79 - 1.42) | **5.46E-11** |
| TG 16:0/18:0/18:1 |  | 0.30 (0.19 - 0.42) | **4.51E-05** |  | 0.82 (0.46 - 1.19) | **3.64E-04** |  | 0.43 (0.30 - 0.56) | **7.53E-10** |  | 1.09 (0.83 - 1.34) | **2.21E-15** |
| TG 16:0/18:1/18:1 |  | 0.20 (0.06 - 0.34) | **2.79E-02** |  | 0.80 (0.37 - 1.23) | **1.92E-03** |  | 0.50 (0.33 - 0.66) | **3.63E-08** |  | 1.32 (1.00 - 1.65) | **5.31E-14** |
| TG 16:0/18:1/18:2 |  | 0.16 (0.02 - 0.29) | 7.84E-02 |  | 0.65 (0.23 - 1.07) | **1.04E-02** |  | 0.46 (0.30 - 0.63) | **2.57E-07** |  | 1.27 (0.95 - 1.59) | **2.98E-13** |
| TG 16:0/18:2/18:2 |  | 0.17 (0.04 - 0.30) | 5.77E-02 |  | 0.64 (0.23 - 1.06) | **1.04E-02** |  | 0.31 (0.16 - 0.47) | **1.88E-04** |  | 0.93 (0.63 - 1.23) | **8.82E-09** |
| TG 16:1/16:1/16:1 |  | 0.05 (-0.08 - 0.18) | 5.75E-01 |  | 0.44 (0.05 - 0.83) | 5.90E-02 |  | 0.05 (-0.08 - 0.18) | 5.64E-01 |  | 0.44 (0.18 - 0.70) | **2.13E-03** |
| TG 16:1/16:1/18:0 |  | 0.21 (0.09 - 0.33) | **7.41E-03** |  | 0.55 (0.17 - 0.94) | **1.69E-02** |  | 0.26 (0.12 - 0.40) | **6.82E-04** |  | 0.82 (0.54 - 1.09) | **2.24E-08** |
| TG 16:1/16:1/18:1 |  | 0.11 (-0.03 - 0.25) | 2.42E-01 |  | 0.74 (0.32 - 1.17) | **4.21E-03** |  | 0.16 (0.01 - 0.32) | 7.49E-02 |  | 0.78 (0.47 - 1.09) | **3.46E-06** |
| TG 16:1/17:0/18:1 |  | 0.08 (-0.06 - 0.22) | 3.82E-01 |  | 0.35 (-0.09 - 0.78) | 1.85E-01 |  | 0.32 (0.16 - 0.47) | **2.55E-04** |  | 0.86 (0.55 - 1.17) | **3.14E-07** |
| TG 16:1/18:1/18:1 |  | 0.11 (-0.03 - 0.25) | 2.22E-01 |  | 0.56 (0.14 - 0.99) | **2.85E-02** |  | 0.20 (0.04 - 0.36) | **3.33E-02** |  | 0.61 (0.28 - 0.94) | **7.13E-04** |
| TG 16:1/18:1/18:2 |  | 0.13 (0.00 - 0.26) | 1.53E-01 |  | 0.54 (0.12 - 0.95) | **3.23E-02** |  | 0.20 (0.04 - 0.36) | **3.00E-02** |  | 0.68 (0.36 - 1.00) | **1.05E-04** |
| TG 17:0/18:1/18:1 |  | 0.16 (0.04 - 0.29) | 5.27E-02 |  | 0.39 (-0.01 - 0.78) | 1.03E-01 |  | 0.36 (0.23 - 0.50) | **1.39E-06** |  | 0.86 (0.59 - 1.13) | **4.45E-09** |
| TG 18:0/18:0/18:1 |  | 0.26 (0.17 - 0.35) | **5.30E-06** |  | 0.54 (0.25 - 0.83) | **1.92E-03** |  | 0.17 (0.08 - 0.25) | **4.02E-04** |  | 0.40 (0.23 - 0.57) | **9.90E-06** |
| TG 18:0/18:1/18:1 |  | 0.30 (0.19 - 0.41) | **4.11E-05** |  | 0.69 (0.34 - 1.05) | **1.61E-03** |  | 0.32 (0.20 - 0.44) | **1.39E-06** |  | 0.75 (0.51 - 0.99) | **7.62E-09** |
| TG 18:0/18:2/18:2 |  | 0.23 (0.11 - 0.36) | **4.85E-03** |  | 0.55 (0.15 - 0.94) | **2.29E-02** |  | 0.07 (-0.04 - 0.18) | 2.59E-01 |  | 0.23 (0.01 - 0.45) | 6.49E-02 |
| TG 18:1/18:1/18:1 |  | 0.19 (0.06 - 0.32) | **2.25E-02** |  | 0.53 (0.13 - 0.94) | **2.93E-02** |  | 0.24 (0.11 - 0.37) | **1.06E-03** |  | 0.55 (0.29 - 0.82) | **1.16E-04** |
| TG 18:1/18:1/18:2 |  | 0.15 (0.01 - 0.28) | 1.18E-01 |  | 0.35 (-0.08 - 0.79) | 1.73E-01 |  | 0.24 (0.09 - 0.39) | **3.66E-03** |  | 0.57 (0.27 - 0.87) | **4.44E-04** |
| TG 18:1/18:1/20:4 |  | 0.17 (0.02 - 0.31) | 8.72E-02 |  | 0.48 (0.03 - 0.93) | 7.83E-02 |  | 0.48 (0.31 - 0.64) | **1.11E-07** |  | 1.12 (0.79 - 1.45) | **2.21E-10** |
| TG 18:1/18:1/22:6 |  | 0.14 (0.02 - 0.26) | 8.72E-02 |  | 0.58 (0.21 - 0.95) | **1.04E-02** |  | 0.51 (0.35 - 0.67) | **3.75E-09** |  | 1.17 (0.85 - 1.48) | **5.30E-12** |
| TG 18:1/18:2/18:2 |  | 0.13 (0.01 - 0.26) | 1.15E-01 |  | 0.42 (0.02 - 0.81) | 7.96E-02 |  | 0.17 (0.03 - 0.31) | **2.98E-02** |  | 0.46 (0.19 - 0.73) | **1.96E-03** |
| TG 18:2/18:2/18:2 |  | 0.10 (-0.01 - 0.20) | 1.70E-01 |  | 0.31 (-0.02 - 0.63) | 1.15E-01 |  | 0.09 (-0.03 - 0.21) | 1.81E-01 |  | 0.29 (0.05 - 0.53) | **2.91E-02** |
| TG 18:2/18:2/20:4 |  | 0.10 (-0.03 - 0.23) | 2.35E-01 |  | 0.31 (-0.10 - 0.71) | 2.12E-01 |  | 0.14 (0.05 - 0.22) | **5.13E-03** |  | 0.31 (0.13 - 0.48) | **1.30E-03** |

^a^ Linear regression of FPG on lipid species adjusted for age, sex, waist circumference and SBP.

^b^ Linear regression of 2h-PLG on lipid species adjusted for age, sex, waist circumference and SBP.

^c^ Beta coefficient (95% confidence intervals) based on an interquartile range increase in predictor lipid species measurement.

^d^ Benjamini-Hochberg corrected p-value. Bold type indicates corrected p < 0.05.
